# Digital insights: Analyzing the reproductive intentions and influencing factors among urban women in China through online platforms

**DOI:** 10.1371/journal.pone.0327570

**Published:** 2025-07-28

**Authors:** Jing Xiang, Xuan Sun

**Affiliations:** 1 Department of Gynecology, Nankai University Hospital, Nankai University, Tianjin, China; 2 Department of Public Administration, Zhou Enlai School of Government, Nankai University, Tianjin, China; McGill University Faculty of Arts, CANADA

## Abstract

**Objective:**

To explore how fertility-related sentiments expressed by urban Chinese women varied over time and were framed in online discourse between 2011 and 2024.

**Methods:**

This study employed natural language processing (NLP), TF-IDF-based semantic analysis, and multinomial logistic regression to analyze user-generated content from Douyin, Xiaohongshu, and Kuaishou. Sentiment categories (positive, neutral, negative) were classified, and predictive variables such as education, marital status, and urban tier were modeled to identify structural correlates of fertility attitudes.

**Results:**

Negative fertility intentions accounted for 71.0% of all discourse, rising sharply from 56.8% in 2021 to 79.8% in 2024. Neutral and positive expressions comprised 28.5% and 0.5%, respectively. Economic constraints—including housing costs (TF-IDF = 2.34)—and environmental concerns (TF-IDF = 2.00) predominated in negative sentiment, while child-rearing costs (TF-IDF = 4.50) were central to neutral positions. Regression analysis revealed marriage was associated with lower odds of negative intention (OR=0.229, 95% CI: 0.194–0.271), while postgraduate education (OR=2.819) and residence in first-tier cities (OR=4.05) were linked to higher odds. Socio-cultural pressures were the most influential predictors of negative sentiment (OR=11.11, 95% CI: 9.07–12.12).

**Conclusion:**

Fertility intentions among urban Chinese women increasingly represent conscious adaptations to complex structural realities rather than simple expressions of personal reluctance. This shift reflects a broader societal transition in which autonomy, well-being, and ethical responsibility gradually supersede traditional reproductive imperatives. Rather than indicating demographic crisis, these changing intentions mark a natural phase of social development, underscoring the need for institutional reforms and a cultural ethos that affirms reproductive freedom and supports parenthood as an informed, empowered choice.

## 1. Background

Global fertility rates have declined markedly in recent decades, prompting growing concern among researchers and policymakers across demographic, economic, and public health domains [1,2]. This trend is particularly acute in East Asia, where countries such as China, Japan, and South Korea face unprecedented demographic challenges related to ultra-low fertility [3–5]. In China, the relaxation of the one-child policy in 2016 failed to reverse the persistent fertility decline. The total fertility rate (TFR) fell to 1.3 in 2020 [6], and despite policy adjustments, it continued to decline, with the United Nations estimating a TFR of 1.00 in 2023 [7]. In the same year, China’s birth population fell to 9.02 million, with a crude birth rate of 6.39 per thousand [8]—among the lowest in recorded history—well below the replacement threshold of 2.1 [9]. This persistent decline signals an urgent need to investigate the socio-economic factors influencing reproductive decisions, particularly among young women, which are especially crucial for forecasting broader demographic patterns.

Emerging research suggests that social media plays a growing role in influencing reproductive attitudes, especially among younger women in urban contexts [10,11]. As interactive platforms increasingly mediate information exposure and value formation, they also become key arenas where fertility-related narratives and sentiments are constructed, contested, and shared in real time. In China, five dominant social platforms—Douyin, Kuaishou, Xiaohongshu, Bilibili, and Weibo—reached a collective 1.071 billion monthly active users (MAUs) by the end of 2024 [12]. Douyin’s dominance as China’s largest short-video platform (743 million Monthly Active Users, or MAU—a metric referring to the number of unique users who engage with a platform at least once within a 30-day period) is undergirded by a 69.2% female user majority [13,14], with 50% of active contributors originating from Tier-1/2 cities. Kuaishou, ranks as the second-largest video application in China, with MAU exceeding 457 million [15], female users represent 44.8% of the platform’s total users, with a significant portion falling within the 18–34 age range [16]. Similarly, Xiaohongshu, a leading platform for lifestyle and consumer content, has seen rapid growth, with 199 million MAU in 2023, 68% of whom are women, predominantly within the same age range as Kuaishou [17]. The significant reach of these platforms across diverse demographics presents a unique opportunity to examine the current broader fertility attitudes among china urban women, especially within the context of China’s rapidly changing demographic landscape. The exclusion of Weibo (485 million MAU,58% entertainment-focused ephemeral content) and Bilibili (363 million MAU, 62% male users) is methodologically necessitated by their demonstrable misalignment with sustained fertility discourse [7].

User-generated content (UGC) on social media platforms offers a substantial repository of spontaneous expressions, reflecting individual beliefs, emotional attitudes, and perceived social norms [18]. Prior research has demonstrated that online discourse influences reproductive decision-making both through direct norm-setting and through more diffuse mechanisms of social reinforcement [19,20]. Despite this relevance, digital fertility discourse remains insufficiently explored in population studies, particularly within the Chinese context. To address this gap, the present study draws on the cognitive-social model of fertility intentions [21] to examine large-scale user-generated content from Douyin, Kuaishou, and Xiaohongshu. These platforms serve as discursive arenas where urban Chinese women articulate a wide spectrum of reproductive attitudes grounded in everyday experience. The analysis provides a cross-sectional snapshot of fertility sentiment as expressed by urban Chinese women in digital narratives, offering insight into how their reproductive perspectives are voiced, contextualized, and situated within contemporary social life.

## 2. Methodology

### 2.1. Data filtering

User-generated content (UGC) related to fertility was retrospectively analyzed based on data collected between May 2021 and April 2024, coinciding with the implementation of China’s three-child policy and providing a relevant policy backdrop. Formal data extraction commenced in May 2024. Content was retrieved via official APIs using a keyword-based filtering strategy informed by predefined thematic categories. The initial keyword list—including terms such as ***“fertility intention,” “pregnancy,” “family formation,”*** and ***“work-life balance”***—was iteratively refined to incorporate emerging topics like ***“policy impact”*** and ***“working mothers.”*** This process yielded content from 23,154 unique user accounts.

**Fig 1:**
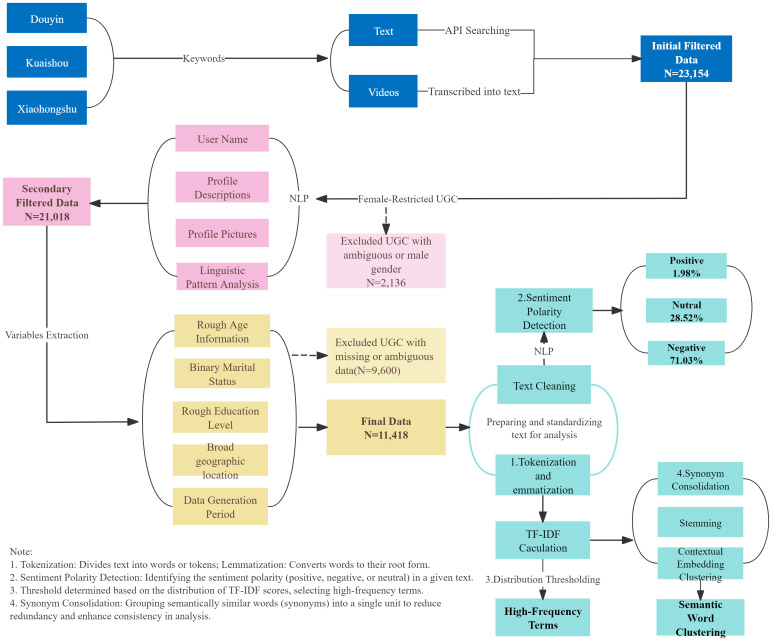
Multi-stage analytic workflow. **Note**:This workflow comprised initial keyword-based data screening, content transcription, semantic preprocessing, TF-IDF term extraction (threshold ≥1.5), and sentiment classification using IBM Watson NLU.

As shown in Fig 1, the analytic workflow followed a multi-stage process. Textual materials were directly processed, while video content was transcribed exclusively to extract thematically relevant information. Only publicly spoken content in Mandarin or widely understood dialects was included; personal introductions, identifiable anecdotes, and background conversations were explicitly excluded. Transcribed texts were immediately anonymized by removing any potentially sensitive or identifying language before thematic analysis.

All data were fully de-identified from the outset. No personally identifiable information (PII), including usernames, profile images, or IP addresses, was retained. Geographic data, where available, were aggregated to the municipal or provincial level, and demographic variables were derived solely from publicly disclosed, non-sensitive statements, in strict adherence to platform policies and internationally recognized research ethics standards.The final anonymized dataset is provided as Supporting Information (S1 Appendix).

No attempts were made to reconstruct individual behavioral profiles or infer latent psychological characteristics beyond the scope of publicly available content. This study fully complies with the Personal Information Protection Law (PIPL) of the People’s Republic of China and relevant platform privacy policies. Given the exclusive use of anonymized, aggregated public data for population-level analysis, the study presents no foreseeable ethical risks related to individual privacy or data security.

#### 2.1.1. Gender-based sample selection.

This study focuses on fertility-related discourse produced by female users, who uniquely experience the physiological, psychological, and socioeconomic burdens of childbearing. These lived experiences inform complex reproductive decisions, making women’s perspectives particularly relevant to fertility research [22]. Despite advancements in gender equality, women remain primary decision-makers in household fertility planning across diverse cultural contexts [23]. In digital spaces, female users also exhibit higher engagement in fertility discussions, while male participation remains limited, reflecting societal perceptions of reproduction as predominantly a female concern [24,25]. These patterns justify focusing the analysis on female-generated content to more accurately capture the complexities of fertility intentions.

Gender classification employed a multi-step inference protocol. User gender was first inferred from publicly available profile metadata, including usernames, self-descriptions, and avatars, and further validated through NLP-based analysis of linguistic cues and behavioral patterns. In the second-stage filtering, 2,136 users (9.2% of the initial 23,154-user corpus) were excluded due to unreliable gender identification or classification as male or gender-neutral, resulting in a dataset composed exclusively of female users for analysis. At no point were profile images, usernames, or IP addresses stored or linked to analytical datasets during this process.

#### 2.1.2. Variables extraction.

Age, education, marital status, and region were selected as key explanatory variables based on their consistently documented relevance to fertility intentions in recent demographic research [26–28]. These variables enable stratified analysis of fertility attitudes across socio-demographic and geographic dimensions.

Data were extracted from user-generated content (UGC) using a combination of direct identification or indirect inference via semantic analysis of publicly available posts. All data processing procedures fully complied with the Personal Information Protection Law (PIPL) of the People’s Republic of China and the privacy policies of the respective platforms. Personally identifiable information (PII) was neither collected nor retained at any stage. Variable extraction was conducted after complete anonymization and content-level aggregation, ensuring both ethical compliance and analytical reliability.

***Age:*** Age was inferred from self-reported temporal cues (e.g., “graduated in 2023”) or generational terms (e.g., “95后”) within publicly visible posts. These references were mapped to analytical age brackets, such as categorizing “95后” as 26–30 years old within the 20–30 group. This indirect, language-based inference avoids sensitive identifiers and is legally permissible under Article 13 of the PIPL when used for academic research on publicly disclosed data.

***Marital Status:*** Relationship status was derived from explicit relational language (e.g., “my husband,” “boyfriend”). For statistical modeling, this was binarized into married and single categories, ensuring analytical simplicity while acknowledging relationship diversity. Only contextually grounded, user-disclosed information was considered, avoiding any identity verification or private data linkage.

***Education Level:*** Educational attainment was assessed via self-reported status or inferred from occupation-related descriptions. Phrases such as “pursuing a master’s degree” or “working on an assembly line” were mapped to three ordinal categories: below bachelor’s, bachelor’s, and post-bachelor’s. All inferences were drawn from public statements and generalized to avoid individual profiling.

***Residence Location:*** Geographic location was triangulated using self-declared city names and platform-assigned, de-identified IP metadata. Discrepancies exceeding 50 km between the two sources triggered exclusion (n = 326, 1.4%). IP-derived data were used solely for immediate province-level verification and discarded without storage. Aggregation ensured that no regional identifiers could be traced back to individual users.

***Data Collection Year:*** Each UGC entry was timestamped at the time of capture. This metadata enabled the temporal mapping of shifts in discourse between 2021 and 2024 and is legally considered non-sensitive information.

During third-stage filtering, data from 9,600 users were excluded due to incomplete, ambiguous, or contextually unresolvable socio-demographic information. This ensured that the final analytical dataset contained only interpretable, non-identifiable demographic indicators suitable for robust statistical modeling. While inference based on indirect cues introduces some uncertainty, stringent safeguards against re-identification were implemented throughout. All findings should therefore be interpreted within these defined methodological and ethical constraints.

### 2.2. Sample weighting adjustment

To correct for potential biases in platform representation and ensure a more accurate sample, a weighting adjustment based on the Monthly Active Users (MAU) ratio was applied. According to QuestMobile 2024 data, the distribution of users across the three platforms was as follows: Douyin 51.5%, Kuaishou 31.7%, and Xiaohongshu 13.8%. Since the original sample was collected using a non-weighted approach, with Douyin having a disproportionately higher representation, the sample was adjusted based on the relative MAU proportions of each platform.

The adjustment process involved calculating the weight for each platform, ensuring that each platform’s representation in the final dataset was proportional to the actual platform user base. The weights were calculated as follows:


$$W_{platform}=\;\frac{{MAU}_{platform}}{\sum_{i=1}^{3}{MAU}_{platform}}$$

Where W_*platform*_ is the weight for each platform, and MAU_*platform*_ represents the monthly active users of each platform. This MAU-proportional weighting approach ensures representativeness relative to each platform’s population penetration rate.

### 2.3. Definition and analysis

#### 2.3.1. Theoretical model of fertility intentions.

In this study, fertility intentions are defined as “individuals’ expressed plans or desires to have children [29], shaped by both personal attitudes and broader structural influences”. This conceptualization integrates the cognitive–social model and the pathway framework [19,30], emphasizing the contextual fluidity of fertility intentions rather than viewing them as fixed behavioral outcomes. Building on this framework, the study analyzes social media discourse to explore how these intentions manifest in real-world narratives. As most users do not explicitly disclose parental status, parity identification is often infeasible unless voluntarily stated. Available data show that 2,154 users (18.9% of the sample) identified as having children, while the majority remain unspecified, resulting in a heterogeneous sample of mothers and non-mothers. Although this limits conventional demographic stratification, it aligns with the study’s attitudinal focus on how reproductive perspectives are shaped within broader life narratives, irrespective of actual childbearing status.

These attitudinal expressions provide meaningful insights when interpreted through the Theory of Planned Behavior (TPB) [31], which conceptualizes intention as a proximal predictor of behavior under favorable conditions. Together, these models offer a layered analytical framework: the cognitive–social model addresses attitude formation, the pathway framework explains attitudinal progression, and TPB clarifies the transition from intention to behavior. This integrated approach broadens the understanding of fertility intentions beyond traditional demographic categorizations.

#### 2.3.2. Sentiment and TF-IDF analysis.

The sentiment analysis framework employed in this study prioritized rigorous text preprocessing to mitigate noise and enhance the semantic fidelity of user-generated content (UGC) related to fertility attitudes. This preparatory phase was foundational to ensuring analytical validity and thematic coherence in downstream sentiment classification.

I. Text Preprocessing

HanLP was employed to segment raw Chinese UGC into linguistically meaningful units, providing the basis for semantic parsing. Stopwords were removed using the HIT Chinese Stopwords list, expanded with fertility-specific terms to suppress high-frequency functional words and platform-specific noise. Informal expressions, including slang and abbreviations, were normalized through a custom mapping system to preserve semantic integrity. Finally, contextual normalization using Baidu’s ERNIE embeddings aligned semantically equivalent phrases (e.g., “生育计划” and “组建家庭”) into unified vector representations, reducing lexical variation without loss of meaning.

II. Sentiment Classification

Sentiment classification was conducted using IBM Watson’s Natural Language Understanding (NLU) platform [32,33], which supports Chinese-language processing through lexical valence dictionaries, negation-sensitive parsing, and contextual embeddings. The system classified text into positive, neutral, and negative categories, accounting for polarity reversals (e.g., negation) and capturing both explicit and nuanced sentiment.

Validation involved benchmarking against the Chinese Sentiment Knowledge Base (CSKB) [34,35], where the model achieved 84.6% accuracy and an F1-score of 0.82 on a test set of 2,000 manually annotated posts. For 12% of the corpus with low-confidence results, sentiment was adjudicated by expert linguistic reviewers, yielding an inter-rater reliability of κ = 0.78. This hybrid validation minimized classification errors from sarcasm, ambiguity, or figurative speech.

The rigor of this process ensured interpretive clarity for downstream analysis of affective attitudes toward fertility within the UGC corpus.

III. TF-IDF

For thematic analysis of external fertility factors, we conducted automated TF-IDF (Term Frequency-Inverse Document Frequency) [36] analysis through NVivo’s algorithmic implementation. This computational method quantifies term significance using a dual weighting mechanism:

i. Term Frequency (TF): Normalized count of a term within individual documents. It is typically calculated as:

$${TF}_{\left(t,d\right)}=\frac{Number\;of\;times\;term\;t\;appears\;in\;document\;d}{Total\;number\;of\;terms\;in\;document\;d}\mathrm{\backslash ]}$$

ii. Inverse Document Frequency (IDF): A logarithmic penalty is applied to terms appearing across multiple documents:

$${IDF}_{\left(t\right)}=log(\frac{N}{{DF}_{\left(t\right)}+1})$$

Where N is the total number of documents in the collection, DF(t) is the number of documents that contain the term t. Laplace smoothing is used to adjust for potential bias caused by term frequency imbalances, with the denominator term adjusted to DF(t)+1 to prevent zero-division errors and reduce statistical instability from out-of-vocabulary and low-frequency terms, such as emerging internet slang in social media data. This approach aligns with standard practices in open-domain text analysis [37,38].

iii. TF-IDF Formula:

TF−IDF(t,d) = TF(t,d)×IDF(t)\]

**Fig 2:**
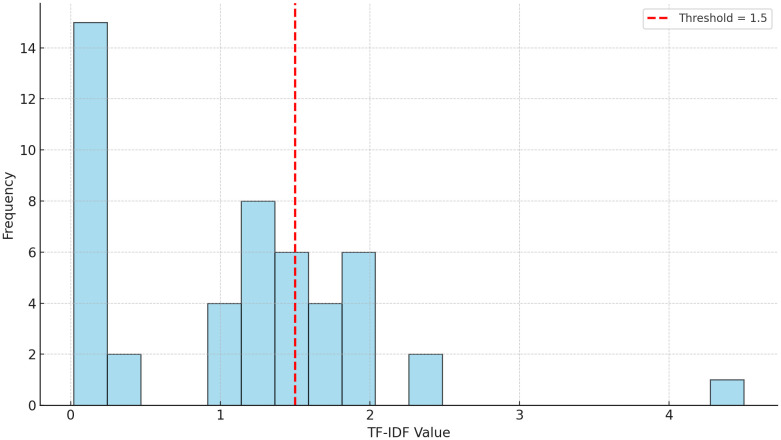
Histogram of TF-IDF values across fertility-related terms. Note: The red dashed line marks the dynamic threshold of 1.5 used to identify high-frequency terms for inclusion in semantic clustering. Terms below this threshold were excluded to enhance analytical precision and reduce noise from diffusely distributed vocabulary.

Table 1 illustrates the application of TF-IDF to fertility-related textual data, emphasizing domain-specific vocabulary and suppressing ubiquitous yet uninformative terms. This generates sparse feature vectors, capturing thematic nuances for further computational analysis, with a dynamically determined TF-IDF threshold of 1.5, derived from score distribution analysis (Fig 2). The dynamic threshold optimizes term retention while minimizing noise, ensuring robust analysis by balancing dimensionality reduction with semantic fidelity, and translating unstructured text into interpretable structured representations.

**Table 1:** Full-process TF-IDF calculation based on preprocessed fertility discourse.

Dataset	Extracted Terms	TF	IDF	TF-IDF Calculation
1.“我想要以后有孩子。(I want to have children in the future.)”	want, future, children	0.33,0.33,0.33	children = log($\frac{3}{2+1}$) = 0 want/future = log($\frac{3}{1+1}$)= 0.1761	children = 0.33*0 = 0 want/future = 0.33 *0.1761 = 0.0581
2.“我觉得抚养孩子太贵了。(I’m afraid that raising children is too expensive.)”	afraid, raise, children, expensive	0.25,0.25,0.25,0.25	afraid...expensive = log($\frac{3}{1+1}$)= 0.1761	afraid...expensive = 0.25*0.1761 = 0.044
3.“有家庭很重要, 但我还没准备好。(It’s important to have a family, but I’m not ready yet.)”	family, important, Not yet, ready	0.25,0.25,0.25,0.25	family...ready = log($\frac{3}{1+1}$)= 0.1761	family...ready = 0.25*0.1761 = 0.044

Semantic clustering was applied to reduce lexical redundancy. Terms were embedded using 300-dimensional fastText vectors and aligned using Chinese WordNet with a cosine similarity threshold ≥ 0.85, consolidating related expressions (e.g., “养育成本” and “经济压力”) under broader thematic constructs such as Economic Constraints. All thematic labels were finalized through expert validation.

A three-tiered validation ensured methodological rigor: internal stability testing (92.3% term overlap; Jaccard index = 0.85), algorithm–expert consensus assessment (Cohen’s κ = 0.71; 89% agreement), and additional diagnostics detailed in S2 Appendix.

### 2.4. Statistical analysis

Chi-square tests were used to examine associations between categorical variables (e.g., age, marital status) and fertility intentions, and to assess the distribution of high-frequency terms identified through TF-IDF across sentiment categories.Collinearity diagnostics were conducted before regression analysis, and only variables with minimal multicollinearity were included in the final multinomial logistic model. This ensured model stability and reliable identification of significant socio-demographic predictors of fertility intentions.

## 3. Results

### 3.1 Demographic differentiation of fertility intentions

Among the 11,418 female participants, fertility attitudes were overwhelmingly negative: 71.0% expressed reluctance toward childbearing, 28.5% remained neutral, and only 0.5% indicated positive intentions (Table 2). The limited positive group (n = 52) exhibited distinct demographic features—primarily married, aged 20–30, with 46.3% from Hunan Province and one-third explicitly linking reproductive willingness to the availability of social support.

**Table 2:** Social and geographical characteristics, and fertility perspectives of network users.

Variables	Fettility Intentions(N,100%)			Sum	Percentage	χ^2^	P
	Negative	Neutral	Positive				
**Marital Status**						1511.47	<0.001
Married	1834,47.84%	1974,51.493%	26,0.68%	3834	33.58%		
Single	6276,82.75%	1282,16.90%	26,0.34%	7584	66.42%		
**Age Interval**						3033.88	<0.001
Under 20	57,8.09%	645,91.49%	3,0.43%	705	6.17%		
20-30	2288,53.81%	1939,45.60%	25,0.59%	4252	37.24%		
Above 30	5765,89.23%	672,10.40%	24,0.37%	6461	56.59%		
**Educational Level**						1602.05	<0.001
Below Bachelor’s	347,25.3%	1011, 73.9%	10,0.7%	1368	12.0%		
Bachelor’s	4165,76.9%	1242,22.9%	8,0.1%	5415	47.4%		
Beyond Bachelor’s	3598,77.6%	1003,21.6%	34,0.7%	4635	40.5%		
**Residence**						2702.03	<0.001
Top Tier Cities^▷^	1719, 72.38%	640, 26.95%	16, 0.67%	2375	20.80%		
Hunan	3788, 84.8%	655, 14.66%	24, 0.54%	4467	39.12%		
Shandong	12, 1.8%	652, 97.75%	3, 0.45%	667	5.84%		
Tianjin	353, 34.88%	651, 64.33%	8, 0.79%	1012	8.86%		
Hubei	2238, 77.25%	658, 22.71%	1, 0.03%	2897	25.37%		
**Data Posted Years**						488.90	<0.001
2021	1108, 56.79%	843, 43.21%	–	1951	17.09%		
2022	1686, 66.96%	787, 31.25%	45, 1.79%	2518	22.05%		
2023	2037, 71.75%	800, 28.18%	2, 0.07%	2839	24.86%		
2024	3279, 79.78%	826, 20.1%	5, 0.12%	4110	36.00%		
**Sum**	8110, 71.03%	3256, 28.52%	52, 1.98%	11418	100.00%		

Note:^▷^Top Tier Cities refers to Beijing, Shanghai, Guangzhou, and Shenzhen.

**Fig 3:**
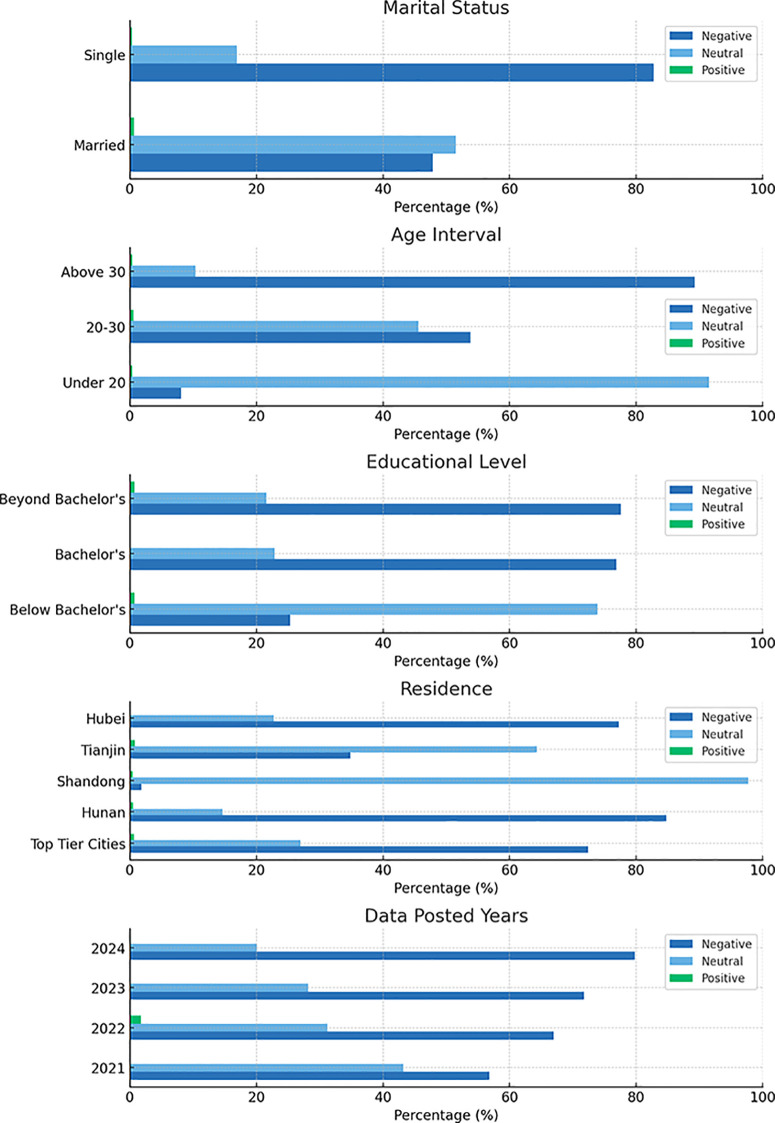
Distribution of Fertility Intentions by Demographic Subgroups. **Note**: The figure illustrates consistent trends of elevated negative sentiment among single, older, and highly educated women, as well as a temporal shift toward reproductive pessimism between 2021 and 2024.

Chi-square analyses revealed significant associations between fertility intentions and all major demographic variables (all p < 0.001), highlighting clear attitudinal stratification by marital status, age, education, and region. These relationships are visually summarized in Fig 3, which presents the distribution of fertility intentions across key demographic subgroups. Unmarried women displayed the highest level of negative sentiment (82.75%) compared to married women (47.84%, χ² = 1,511.47), suggesting that marital status may partially buffer reproductive pessimism. Age stratification revealed a neutral attitudinal majority among adolescents under 20 (91.49%), but negative sentiment escalated in older groups, reaching 89.23% among women over 30 (χ² = 3,033.88), potentially reflecting cumulative life-course pressures.

Educational attainment showed a progressive inverse relationship with reproductive positivity: negative sentiment increased from 25.3% among less-educated women to 76.9% among bachelor’s degree holders and 77.6% among postgraduates (χ² = 1,602.05). Spatial patterns followed similar gradients. First-tier cities, marked by intense socioeconomic pressures, exhibited the most negative skew (72.38%, χ² = 2,702.03), while provinces such as Shandong maintained predominantly neutral attitudes (97.75%), indicating regional divergence in reproductive perception.

Across the study period, negative attitudes intensified markedly—from 56.79% in 2021 to 79.78% in 2024—mirroring a concurrent decline in neutral sentiment (from 43.21% to 20.10%; χ² = 488.90). This temporal trend reflects a rapid shift toward reproductive pessimism.

### 3.2. Salient lexical patterns in fertility discourse

**Fig 4:**
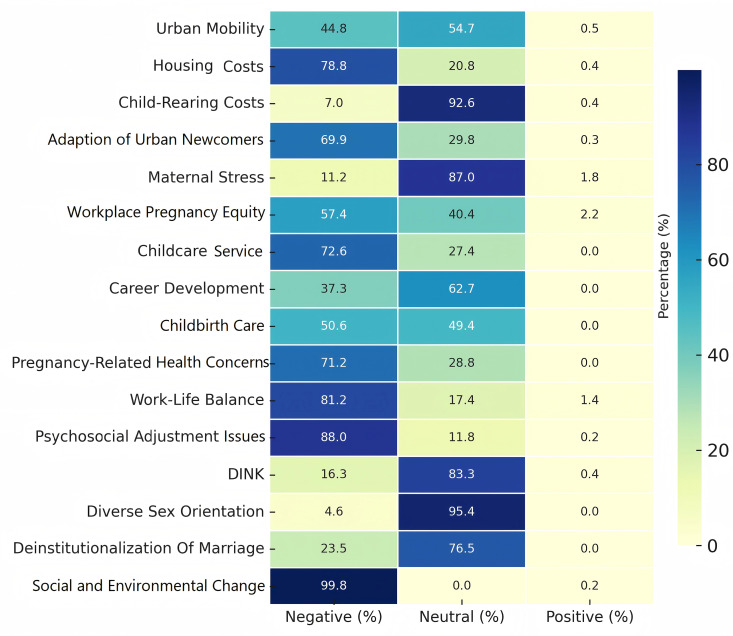
Percentage Distribution of Fertility Intentions Across External Factors. Note: Percentage Distribution of Fertility Intentions Across External Factors. This heatmap visualizes how various external factors, such as urban mobility, housing costs, and child-rearing costs, impact fertility intentions, with a clear delineation of negative, neutral, and positive responses based on these influences.

The textual landscape of fertility intentions was constructed through an integrative computational analysis, combining Chi-square validation with TF-IDF semantic weighting, showed in Table 3. Sixteen recurrent terms were extracted from the upper ranks of TF-IDF scoring, reflecting discourse patterns related to economic conditions, sociocultural norms, psychosocial pressures, and reproductive support structures. Among these, high-frequency entries were identified based on dynamic thresholding and visualized in an attitude distribution heatmap (Fig 4), where chromatic gradients captured sentiment-term co-occurrence intensities across the discourse corpus, independent of but complementary to TF-IDF metrics.

**Table 3:** Quantitative Analysis of External Factors Associated with Fertility Intentions.

Categories	Extracted Terms	χ²	P	TF-IDF (Negative)	TF-IDF (Neutral)	TF-IDF (Positive)
**Socioeconomic Status**	Urban Mobility	3.60	<0.001	1.23	1.49	0.02
	Housing cost*	11.84	<0.001	2.34	1.12	0.04
	Child-Rearing Expenses*	2.01	<0.05	0.35	4.50	0.03
	Adaptation of Urban Newcomers*	7.93	<0.05	1.78	1.35	0.05
**Social Support**	Maternal Stress*	1.96	0.05	1.02	1.65	0.06
	Workplace Pregnancy Equity*	4.01	<0.01	1.56	1.43	0.09
	Childcare Services*	3.80	<0.01	1.89	1.20	0.07
	Career Advancement and Personal Goals*	4.51	<0.01	2.35	1.98	0.06
**Social Psychological Factors**	Childbirth Care*	5.33	<0.05	1.75	1.20	0.12
	Pregnancy-Related Health Concerns*	7.12	<0.01	1.88	1.30	0.15
	Work-Life Balance	4.20	<0.01	1.45	1.10	0.08
	Psychosocial Adjustment Issues*	6.50	<0.05	2.00	1.35	0.18
**Socio-Cultural Factors**	DINK (Dual Income No Kids)*	2.87	<0.05	1.90	1.45	0.05
	Diverse Sexual Orientations (e.g., Lesbian)	3.10	<0.05	1.15	1.20	0.20
	Deinstitutionalization Of Marriage*	6.80	<0.05	1.65	1.10	0.25
	Social and Environmental Change*	7.75	<0.01	2.00	1.50	0.12

Table Note:

1. Extracted Terms: Terms were extracted using TF-IDF analysis from UGC on social media platforms. A dynamic threshold of 1.5 was applied to identify high-frequency terms, marked with an asterisk (*). These terms are considered highly relevant to fertility intentions. Terms below the threshold, while still meaningful, provide broader context to the fertility discussion. Definitions of all extracted external factors can be found in S3 Appendix.

2. χ2: Represents the statistical significance of the relationship between each term and fertility intentions; P: Shows the p-value for each term, with values <0.05 indicating significant relationships.

TF-IDF (Negative), TF-IDF (Neutral), TF-IDF (Positive): TF-IDF scores for each term in relation to negative, neutral, and positive fertility intentions, respectively·

Negative sentiment was most consistently anchored by high-weight terms such as housing cost (TF-IDF = 2.34), career advancement and personal goals (TF-IDF = 2.35), and psychosocial adjustment issues (TF-IDF = 2.00), reflecting a convergence of material insecurity and individual constraint. Childbirth care (TF-IDF = 1.75) and pregnancy-related health concerns (TF-IDF = 1.88) further highlighted health-related apprehensions, reinforcing a narrative of cumulative vulnerability.

In the neutral sentiment group, child-rearing expenses stood out with the highest TF-IDF score across the corpus (TF-IDF = 4.50), co-occurring with 92.6% of neutral posts. Despite its conventional interpretation as a fertility barrier, its positioning here suggests a more contingent form of hesitation—where financial strain informs postponement rather than rejection. Other moderately weighted terms such as maternal stress (TF-IDF = 1.65) and workplace pregnancy equity (TF-IDF = 1.43) underscore this theme of cost-benefit ambivalence without tipping into pessimism.

Some lexical items presented subtle internal contradictions. For instance, childcare services (TF-IDF = 1.20) appeared more prominently in neutral posts, yet in discourse it often accompanied complaints about systemic inadequacies. Similarly, social and environmental change (TF-IDF = 2.00 in negative, 1.50 in neutral, 0.12 in positive) showed dispersed sentiment distribution, reflecting complex user interpretations of risk and transformation.

Positive sentiment, though rare, was lexically associated with deinstitutionalization of marriage (TF-IDF = 0.25) and diverse sexual orientations (TF-IDF = 0.20), suggesting a distinct set of reproductive values detached from traditional family structures. While statistically marginal, these terms point to emerging minority perspectives embracing alternative fertility ideologies.

Terms with low weight or diffuse sentiment alignment, such as urban mobility (TF-IDF = 1.23), were excluded from further modeling. Overall, the evidence demonstrates that lexical signals of fertility intention cluster meaningfully around distinct psychological and structural domains, each shaping emotional stance in non-binary and sometimes ambivalent ways.

### 3.3. Multinomial logistic regression analysis

**Fig 5:**
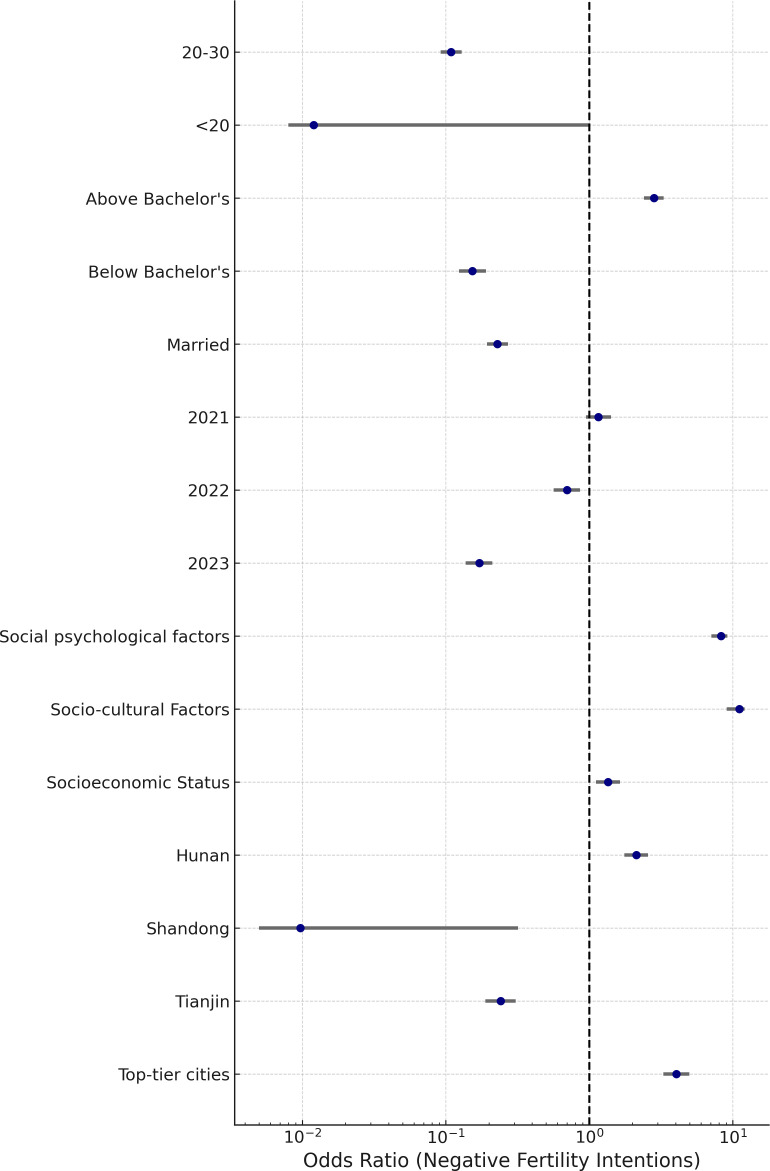
Adjusted Predictors of Negative Fertility Intentions.

**Fig 6:**
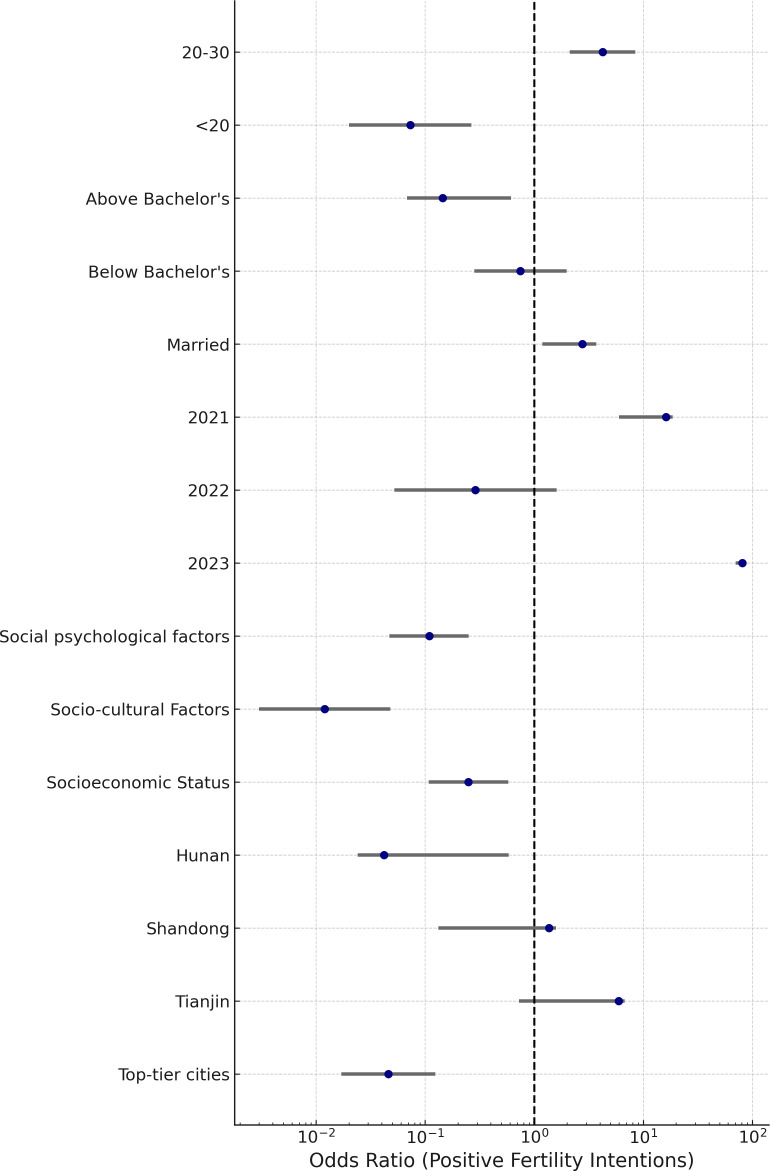
Adjusted Predictors of Positive Fertility Intentions. **Note**: Forest plot illustrating the results of the multinomial logistic regression model, with neutral fertility intention as the reference category. Each horizontal line represents a 95% confidence interval for a given predictor, and the central marker denotes the adjusted odds ratio (OR). ORs > 1 indicate an increased likelihood of expressing either negative or positive fertility sentiment relative to neutral intention, while ORs < 1 reflect a reduced likelihood. The model was adjusted for multicollinearity (VIF < 1.4), and only statistically significant predictors (p < 0.05) are displayed.

The multinomial logistic regression model, validated through collinearity diagnostics (Tolerance: 0.715–0.957; VIF: 1.045–1.398), quantified the distinct associations between structural, psychological, and temporal variables and fertility intentions, using neutral attitudes as the reference category (Table 4 and Table 5). The effect sizes and confidence intervals, presented in Figs 5 and 6, demonstrate the asymmetrical patterns underlying negative and positive fertility sentiments.

**Table 4:** Collinearity Diagnostics for Independent Variables.

Cofficient^a^								
Variable	B	Beta	t	P	95% CI	SE	Tolerance	VIF
(Constant)	0.393		16.838	0.000	0.347 - 0.438	0.023		
**Age Range**	0.317	0.421	45.987	0.000	0.303 - 0.330	0.007	0.736	1.359
**External Factors**	0.042	0.092	11.143	0.000	0.035 - 0.049	0.004	0.911	1.097
**Marital Status**	0.167	0.172	18.525	0.000	0.150 - 0.185	0.009	0.715	1.398
**City of Residence**	−0.026	−0.072	−8.449	0.000	−0.032 - 0.02	0.003	0.857	1.167
**Education Level**	0.054	0.081	9.604	0.000	0.043 - 0.065	0.006	0.874	1.145
**Data Collected Years**	0.039	0.1	12.494	0.000	0.033 - 0.045	0.003	0.957	1.045

^a^Dependent Variable: Fertility Intention.

**Table 5:** The Multinomial Logistic Regression Model.

Fertility intentions^a^		B	S.D	P	OR	95% CI	
						lower	upper
**Negative**	intercept	3.209	0.131	<0.001			
	**Marrital status**						
	Married	−1.472	0.085	<0.001	0.229	0.194	0.271
	Single(ref)						
	**Education**						
	Below Bachelor’s	−1.874	0.111	<0.001	0.153	0.124	0.191
	Above Bachelor’s	1.036	0.08	<0.001	2.819	2.408	3.3
	Bachelor’s(ref)						
	**Affect factors**						
	Socio-cultural Factors	2.407	0.131	<0.001	11.11	9.07	12.116
	Socioeconomic Status	0.302	0.099	0.002	1.352	1.114	1.641
	Social psychological factors	2.114	0.125	<0.001	8.28	7.095	9.154
	Social Support(ref)						
	**Residence**						
	Tianjin	−1.423	0.124	<0.001	0.241	0.189	0.307
	Top tier cities	1.399	0.107	<0.001	4.051	3.286	4.995
	Hunan	0.755	0.097	<0.001	2.127	1.759	2.572
	Shandong	−4.645	0.329	<0.001	0.0097	0.005	0.318
	Hubei(ref)						
	**Age Interval**						
	<20	−4.437	0.18	<0.001	0.012	0.008	1.017
	20-30	−2.217	0.086	<0.001	0.109	0.092	0.129
	>30(ref)						
	**Data Collected Years**						
	2021	0.147	0.103	0.153	1.159	0.947	1.418
	2022	−0.358	0.107	0.001	0.699	0.566	0.863
	2023	−1.766	0.108	<0.001	0.171	0.138	0.211
	2024(ref)						
**Positive**	intercept	−5.405	1.192	<0.001			
	**Marrital status**						
	Married	1.014	0.347	0.003	2.756	1.184	3.716
	Single(ref)						
	**Education**						
	Below Bachelor’s	0.291	0.498	0.558	0.747	0.282	1.982
	Above Bachelor’s	−1.934	0.415	<0.001	0.145	0.068	0.613
	Bachelor’s(ref)						
	**Affect factors**						
	Socio-cultural Factors	−4.401	0.699	<0.001	0.012	0.003	0.048
	Socioeconomic Status	−1.389	0.429	0.001	0.249	0.108	0.578
	Social psychological factors	−2.218	0.427	<0.001	0.109	0.047	0.251
	Social Support(ref)						
	**Residence**						
	Tianjin	1.782	1.074	0.097	5.94	0.724	6.742
	Top tier cities	−3.089	1.057	0.003	0.046	0.017	0.124
	Hunan	−3.165	1.034	0.002	0.042	0.024	0.582
	Shandong	0.311	1.193	0.794	1.365	0.132	1.151
	Hubei(ref)						
	**Age Interval**						
	<20	−2.62	0.66	<0.001	0.073	0.02	0.265
	20-30	1.447	0.345	<0.001	4.25	2.12	8.46
	>30(ref)						
	**Data Collected Years**						
	2021	2.783	0.506	<0.001	16.166	6.002	18.547
	2022	−1.242	0.874	0.155	0.289	0.052	1.602
	2023	4.396	2.18	0.004	81.06	70.338	85.148
	2024(ref)						

Married women were significantly less likely to express negative intentions (OR = 0.229, 95% CI: 0.194–0.271) and more inclined toward positive responses (OR = 2.756, 95% CI: 1.184–3.716), relative to those with neutral attitudes—suggesting that institutional or economic security continues to play a buffering role. However, the limited expression of positivity even within this group hints at a more fragile effect than traditional frameworks might imply.

Education introduced a bifurcation. Women with less than a bachelor’s degree showed an 85% decrease in the likelihood of negative expression (OR = 0.153, 95% CI: 0.124–0.191), but no significant shift toward positivity. Conversely, postgraduates were nearly three times more likely to express negativity (OR = 2.819, 95% CI: 2.408–3.300) and significantly less likely to report positive views (OR = 0.145, 95% CI: 0.068–0.613), reflecting perceived opportunity costs and possible ideological divergence from normative expectations.

Among affective dimensions, socio-cultural burden exhibited the most polarized pattern: it dramatically increased the odds of negativity (OR = 11.110, 95% CI: 9.070–12.116) while virtually eliminating the chance of positivity (OR = 0.012, 95% CI: 0.003–0.048). Psychological stressors followed a similar trajectory (OR = 8.280 for negative; OR = 0.109 for positive), and socioeconomic pressures also aligned with this pattern, albeit more moderately (OR = 1.352 for negative; OR = 0.249 for positive).

Regional effects further reinforced these divides. Women in top-tier cities were four times more likely to report negative intentions (OR = 4.051, 95% CI: 3.286–4.995), and 95% less likely to report positive ones (OR = 0.046, 95% CI: 0.017–0.124), compared to those in Hubei. A similar pattern appeared in Hunan (OR = 2.127 for negative; OR = 0.042 for positive), while Tianjin showed a notable reduction in negative intent (OR = 0.241, 95% CI: 0.189–0.307) but no corresponding uptick in positive orientation.

Age patterns revealed a clear life-course gradient. Women under 20 were largely absent from both ends of the spectrum (OR = 0.012 for negative; OR = 0.073 for positive), suggesting delayed engagement with fertility decisions. In contrast, those aged 20–30 exhibited a strong positive orientation (OR = 4.250, 95% CI: 2.120–8.460) and a notably reduced likelihood of negative responses (OR = 0.109, 95% CI: 0.092–0.129), identifying them as a key transitional group.

Temporal shifts suggest rising pessimism. Compared to 2024, women surveyed in 2023 were significantly less likely to express negativity (OR = 0.171, 95% CI: 0.138–0.211) and far more likely to report positive intentions (OR = 81.060, 95% CI: 70.338–85.148). This optimism was also evident in 2021 (OR = 16.166 for positive), but absent in 2022, which showed a moderate decline in negative sentiment (OR = 0.699) without a corresponding rise in positivity (p = 0.155).

Collectively, these findings highlight that fertility intentions are not simply attitudinal gradients but structurally segmented orientations. Negative responses reflect widespread yet diffuse suppression, while positive attitudes are confined to narrower ideological and demographic zones. These outcomes are not mirror opposites, but reflect distinct logics shaped by social context.

## 4. Discussion

This study presents a comprehensive examination of how fertility intentions among urban Chinese women are shaped and articulated amidst escalating structural pressures. Over the study period, reproductive pessimism increased by more than 20 percentage points, indicating a growing gap between reproductive aspirations and perceived feasibility. Rather than indicating a mere decline in fertility desire, this trend suggests a broader pattern of attitudinal disengagement that arises from the cumulative effects of economic, institutional, cultural, and ecological pressures. Framed within the cognitive-social model, these intentions emerge as adaptive responses to the dynamic interplay between internal evaluations and external structural limitations. They represent not merely personal reluctance but also a broader cognitive and emotional recalibration of reproductive decisions in the context of modern urban environments.

Economic insecurity emerged as a consistently salient factor associated with negative fertility attitudes, particularly in relation to gendered labor dynamics and institutional constraints—a pattern further contextualized through observed discourse on educational stratification, workplace inequality, and social support inadequacy. Housing costs (TF-IDF = 2.34) and child-rearing expenses (TF-IDF = 4.50) were consistently identified as central financial stressors across discourse and regression models. While 33 Chinese cities surpassed the World Bank’s high-income GDP threshold by 2023 [39]—this concentration of wealth has intensified cost-of-living pressures. Paradoxically, economic advancement exacerbates reproductive constraints by amplifying financial thresholds for family formation. Housing unaffordability—fueled by population concentration, speculative markets, and land scarcity—serves not only as a material obstacle but as a symbolic deterrent [40–43]. A Beijing mother’s remark that her ¥7.2 million mortgage constitutes “not just debt but a 30-year sentence that precludes children’s futures” vividly captures the symbolic weight of homeownership in China’s post-reform social contract. Property ownership functions not only as a material asset but increasingly as a perceived prerequisite for intergenerational stability. This reality is further compounded by the substantial financial burden of raising a child in China, estimated at approximately 680,000 yuan from birth to university graduation [44]. Such pressures place significant strain on women balancing family planning with economic stability. These dynamics mirror patterns in other low-fertility societies, including Italy and Norway, where economic affluence paradoxically coexists with widespread reproductive pessimism [45,46].

Age and educational attainment interact to deepen the structural disincentives surrounding fertility decisions. As women pursue higher education, the extended duration of academic engagement delays entry into reproductive life stages, deferring marriage and parenthood. Beyond postponement, this trajectory increases exposure to work environments where the opportunity costs of motherhood grow more pronounced [47]. Regression results confirm that both increasing age and higher educational attainment are associated with greater likelihood of negative fertility intentions and diminished positive orientation—a trend consistent with patterns observed across other low-fertility contexts [48,49].For highly educated women, transitioning into the labor market often brings intensified exposure to institutional barriers. In China, recent evidence highlights heightened work–family conflicts that lead many women to postpone or forgo parenthood altogether [50, 51]. Discourse-level analysis supports this: TF-IDF scores for terms like “career advancement” (2.35) and “workplace pregnancy equity” (1.56) reflect sustained anxiety over hiring discrimination, career stagnation, and long-term wage penalties. These findings are echoed in international literature indicating that in the absence of institutional support, motherhood is increasingly framed as a high-risk, low-reward decision, particularly for professional women [52].

Workplace norms and policy infrastructures critically mediate this calculus. Nordic countries have historically offered generous support—universal childcare, gender-equal parental leave—which enables 89% maternal labor market reentry and reduces work–family tension [53]. However, recent fertility declines in the region suggest that institutional generosity alone may not be sufficient to sustain reproductive optimism. In contrast, China’s fragmented childcare system and minimal paternal leave—averaging just 15 days, with two-thirds of fathers taking fewer than five—continue to reinforce the male-breadwinner and female-caregiver model [54,55]. These systemic gaps leave women to absorb disproportionate burdens during the perinatal period and beyond, particularly in high-pressure professional settings. The resulting conflict between career advancement and childbearing reflects not only personal trade-offs but broader institutional inertia, reinforcing structural gender inequality across both public and private domains.

Institutional pressures also extend into the private sphere. Marriage continues to influence fertility intentions but in more complex ways than traditionally assumed. While regression results indicated that married women were less likely to express negative intentions (OR = 0.229) and more likely to express positive ones (OR = 2.756), the broader discourse reveals a striking divergence from this statistical trend. Even among married women, positive sentiment was notably subdued, suggesting that the institution of marriage—despite its traditionally protective role in fertility, as frequently emphasized in demographic literature [23,41]—no longer guarantees emotional affirmation or perceived readiness for parenthood. This disconnect reflects a growing skepticism toward the protective value of marriage, particularly in contexts where women continue to bear a disproportionate share of domestic responsibilities.According to national surveys, married women in China perform on average 1.35 more hours of housework per day than men and account for 73% of all childcare responsibilities [56–58]—an embodiment of the “second shift,” where women shoulder a dual burden of paid and unpaid labor within the household [59]. This imbalance is not merely symbolic; it manifests in tangible material penalties—most notably, a 49% earnings reduction post-childbirth, a disparity absent among men and largely driven by prolonged career interruptions, reduced working hours, and limited promotion opportunities stemming from caregiving responsibilities [60]. In this light, marriage amplifies rather than mitigates reproductive risk. Its institutional formality offers symbolic security, but little practical redistribution of risk or care.

Cultural reconfigurations are simultaneously challenging normative expectations of reproduction. The analysis identified growing discourse around DINK lifestyles, voluntary childlessness, and the retreat from marriage as a reproductive institution—particularly among younger, educated urban women. These choices are increasingly presented as rational, empowered responses to a precarious and uncertain world, reflecting Inglehart’s post-materialist value thesis that prioritizes self-actualization over traditional family obligations [61]. Terms like “DINK” (TF-IDF = 1.90) and “deinstitutionalization of marriage” (TF-IDF = 1.65) demonstrated significant salience in negative intention discourse, supported by regression results linking socio-cultural framing to an elevenfold increase in negative sentiment (OR = 11.110) and a sharp decline in positive expression (OR = 0.012). References to “self-partnered” life choices (e.g., Xiaohongshu user: “I have no need for a wedding to validate my life choices”) reflect the normalization of reproductive nonconformity. These trajectories, consistent with findings in Western societies [62, 63], often represent a pursuit of personal freedom and relational autonomy rather than outright rejection of cultural norms. Importantly, such narratives are disproportionately voiced by individuals who do not cite financial barriers, implying that higher socio-economic capital enables greater latitude to opt out of reproductive expectations. This discursive evolution is thus both ideational and stratified: agency is not uniformly distributed but shaped by access to resources, education, and relational independence.

A novel dimension emerged in the form of ecological anxiety. “Social and environmental change” (TF-IDF = 2.00) frequently co-occurred with negative attitudes, accompanied by expressions such as “this world is a death sentence for the next generation.” These sentiments, aligned with Schneider-Mayerson and Leong’s theory of eco-reproductive ethics, are grounded in lived concerns about climate instability, environmental degradation, and global political uncertainty. Unlike economic pressures, ecological anxieties are more ideologically encoded and largely absent in positive discourse, suggesting a deeply moralized framing of reproductive restraint. This asymmetric semantic distribution reinforces that while negative intentions are multidimensional, positive ones tend to cluster around a narrower band of structural security. Recent systematic reviews further underscore this trend, positioning voluntary childlessness as a responsible ethical choice in the face of climate crisis [64,65]. In China’s densely populated urban centers, such expressions reflect a psychological burden that transcends material calculations, underscoring the growing moralization and existential framing of fertility decisions within the Anthropocene context.

Taken together, these findings reveal a fundamental misalignment between individual reproductive capacities and the institutional, economic, and sociocultural conditions in which reproductive decisions unfold. Fertility intentions—framed through cognitive–social, behavioral, and motivational lenses—are not isolated reflections of personal desire, but adaptive responses to systemic constraints that increasingly render parenthood a high-risk endeavor. It is both analytically reductive and ethically problematic to interpret declining fertility as the failure of individual women; rather, it reflects a rational recalibration in the face of unmet structural support and institutional inertia. This shift marks a broader trajectory in human development, from biologically driven reproduction toward a deliberate pursuit of autonomy, well-being, and moral agency. As material security expands, individuals prioritize reflective life planning over normative imperatives, reshaping fertility as a matter of choice rather than obligation. In this context, fertility decline should not be pathologized as a demographic crisis, but recognized as a transitional expression of modernity—one that redefines human value beyond reproduction toward equity, sustainability, and self-determination. Responding to this transformation requires not only structural reform—gender-equitable labor protections, universal childcare, and inclusive parental leave—but also a cultural realignment that affirms reproductive autonomy as a socially supported, ethically grounded, and individually empowered choice.

## 5. Limitations

This study provides insight into fertility intentions among urban Chinese women through large-scale user-generated content, yet several methodological and interpretive limitations remain.

The sample is inherently selective, shaped by the demographics of active social media users—typically younger, urban, and more educated—thereby limiting the generalizability of findings beyond this population segment. The cross-sectional nature of the data also restricts inference on long-term intention trajectories or behavioral outcomes. Socio-demographic attributes were inferred through linguistic markers rather than self-report, introducing classification uncertainty despite multi-stage validation procedures. Additionally, the use of culturally specific metaphors and figurative language may obscure semantic clarity in ways not easily captured by computational models. Sentiment classification, while implemented through an established neural network system (IBM Watson NLU), reflects the interpretive constraints of its training corpus. Outputs should be understood as probabilistic estimations rather than objective representations of affective stance. Particularly in high-context languages such as Chinese, rhetorical nuance and moral framing may challenge sentiment polarity schemes.

Although the methodological framework employed here allows for consistent large-scale analysis, caution is warranted when interpreting affective or demographic patterns as definitive. Future research would benefit from incorporating longitudinal data, offline triangulation, and culturally grounded lexical resources to enhance analytical depth and reliability.

## 6. Conclusion

Fertility intentions among urban Chinese women are increasingly shaped not by isolated personal preference, but by how society allocates risk, opportunity, and care. This shift reveals deeper tensions between demographic expectations and the lived conditions in which life decisions unfold. Addressing this disconnect is not only a matter of reversing fertility decline, but of reimagining what social systems must provide to make parenthood a viable and valued choice.

## Supporting information

S1 Appendix De-identified Data for Fertility Intentions. Anonymized dataset of female user-generated content with extracted socio-demographic variables used for analysis.
(XLSX)

S2 Appendix TF-IDF Validation and Sentiment Alignment. Validation of term selection and sentiment classification, including expert ratings and statistical robustness checks.
(PDF)

S3 Appendix Definitions of External Factors. Detailed descriptions and sentiment labels of external factors influencing fertility intentions, grouped by thematic category.
(PDF)

## References

[1] United Nations Department of Economic and Social Affairs, Population Division. World Population Prospects 2024: Summary of Results [Internet]. New York: United Nations; 2024 [cited 2024 Jul 9]. Available from: https://population.un.org/wpp/publications/.

[2] Nakatani H. Ageing and shrinking population: The looming demographic challenges of super-aged and super-low fertility society starting from Asia. Glob Health Med. 2023;5(5):257–63. doi: 10.35772/ghm.2023.01057 37908514 PMC10615022

[3] Luo Y, Su B, Zheng X. Trends and Challenges for Population and Health During Population Aging - China, 2015-2050. China CDC Wkly. 2021;3(28):593–8. doi: 10.46234/ccdcw2021.158 34594944 PMC8393078

[4] Oizumi R, Inaba H, Takada T, Enatsu Y, Kinjo K. Sensitivity analysis on the declining population in Japan: Effects of prefecture-specific fertility and interregional migration. PLoS One. 2022;17(9):e0273817. doi: 10.1371/journal.pone.0273817 36103457 PMC9473415

[5] Yoo SH, Agadjanian V. The paradox of change: Religion and fertility decline in South Korea. DemRes. 2021;44:537–62. doi: 10.4054/demres.2021.44.23

[6] National Bureau of Statistics of China. Main Data of the Seventh National Population Census. 2021. Available from: https://www.stats.gov.cn/english/PressRelease/202105/t20210510_1817185.html

[7] United Nations Department of Economic and Social Affairs, Population Division. World Population Prospects 2024: Summary of Results [Internet]. New York: United Nations; 2024 [cited 2025 May 10]. Available from: https://www.un.org/development/desa/pd/content/world-population-prospects-2024-summary-results-0

[8] China Economic Net. National Population Changes Sampling Survey Data [Internet]. 2024 Jan 18 [cited 2024 May 25]. Available from: http://www.ce.cn/xwzx/gnsz/gdxw/202401/18/t20240118_38870849.shtml

[9] Craig J. Replacement level fertility and future population growth. Popul Trends. 1994;(78):20–2. 7834459

[10] Smith M, Mitchell AS, Townsend ML, Herbert JS. The relationship between digital media use during pregnancy, maternal psychological wellbeing, and maternal-fetal attachment. PLoS One. 2020;15(12):e0243898. doi: 10.1371/journal.pone.0243898 33326465 PMC7743947

[11] Liu B. Research on the Influence of Douyin’s Short Video on Love on Contemporary Chinese Female College Students’ Concept of Marriage and Love. CHR. 2023;5(1):230–7. doi: 10.54254/2753-7064/5/20230232

[12] QuestMobile Research Institute. QuestMobile 2024 new media ecosystem review: Top five platforms cover 1.071 billion users, content commercialization enters explosive growth phase… [Internet]. 2024 Dec 24 [cited 2025 Apr 7]. Available from: https://www.questmobile.com.cn/research/report/1871401635281997826

[13] QuestMobile. QuestMobile 2024 ‘She Economy’ Insights Report [Internet]. 2024 Mar 6 [cited 2024 May 20]. Available from: https://www.questmobile.com.cn//research/report/1764860900811051009

[14] Zeng S. Chinese female representation on short video applications and their perception: Douyin (TikTok) as a case study [dissertation]. Portugal: Universidade Catolica Portuguesa; 2023 [cited 2024 Apr 7]. Available from: ProQuest Dissertations & Theses database. Document ID: 2957139934.

[15] Kuaishou Technology. 2023 annual report [Internet]. 2024 Apr 22 [cited 2025 Apr 7]. Available from: https://ir.kuaishou.com/system/files-encrypted/nasdaq_kms/assets/2024/04/22/19-26-33/2023%20AR_EN.pdf

[16] China Trading Desk. Kuaishou’s Chinese New Year insights: A deep dive into user data and trends [Internet]. 2023 Jan 21. Available from: https://www.chinatradingdesk.com/post/kuaishou-s-chinese-new-year-insights-a-deep-dive-into-user-data-and-trends

[17] Qian-gua Data. 2024 Active User Research Report (Little Red Book Platform) [Internet]. 2024 Apr 10 [cited 2024 Jun 1]. Available from: https://www.qian-gua.com/blog/detail/2898.html

[18] Chung DT. How user-generated content on social media platform can shape consumers’ purchase behavior? An empirical study from the theory of consumption values perspective. Cogent Business & Management. 2025;12(1). doi: 10.1080/23311975.2025.2471528

[19] Khadivzadeh T, Latifnejad Roudsari R, Bahrami M, Taghipour A, Abbasi Shavazi J. The influence of social network on couples’ intention to have the first child. Iran J Reprod Med. 2013;11(3):209–18. 24639748 PMC3943220

[20] Wijenayake S, van Berkel N, Kostakos V, Goncalves J. Quantifying determinants of social conformity in an online debating website. Int J Hum Comput Stud. 2022;158:102743. doi: 10.1016/j.ijhcs.2021.102743

[21] Bachrach CA, Morgan SP. A Cognitive-Social Model of Fertility Intentions. Popul Dev Rev. 2013;39(3):459–85. doi: 10.1111/j.1728-4457.2013.00612.x 25132695 PMC4132897

[22] World Health Organization. Gender and health [Internet]. Geneva: World Health Organization; 2021 May 24 [cited 2025 Apr 12]. Available from: https://www.who.int/news-room/questions-and-answers/item/gender-and-health

[23] Speizer IS, Calhoun LM. Her, his, and their fertility desires and contraceptive behaviours: A focus on young couples in six countries. Glob Public Health. 2022;17(7):1282–98. doi: 10.1080/17441692.2021.1922732 33939936 PMC8563483

[24] Hives L, Bray EP, Nowland R, Thomson G. Social media influencers in the space of pregnancy and parenting: a scoping review protocol. BMJ Open. 2024;14(10):e087200. doi: 10.1136/bmjopen-2024-087200 39424382 PMC11492936

[25] Grace B, Shawe J, Johnson S, Stephenson J. You did not turn up… I did not realise I was invited…: understanding male attitudes towards engagement in fertility and reproductive health discussions. Hum Reprod Open. 2019;2019(3):hoz014. doi: 10.1093/hropen/hoz014 31218265 PMC6573469

[26] Adhikari S, Lutz W, KC S. Rural/urban fertility differentials and the role of female education in declining birth rates: comparative analysis in Asia, Africa, and Latin America. Asian Population Studies. 2024;:1–25. doi: 10.1080/17441730.2024.2430035

[27] Kebede E, Striessnig E, Goujon A. The relative importance of women’s education on fertility desires in sub-Saharan Africa: A multilevel analysis. Popul Stud (Camb). 2022;76(1):137–56. doi: 10.1080/00324728.2021.1892170 33724165

[28] Fiskin G, Sari E. Evaluation of the relationship between youth attitudes towards marriage and motivation for childbearing. Children and Youth Services Review. 2021;121:105856. doi: 10.1016/j.childyouth.2020.105856

[29] Kumari B, Hajela K, Ali A, Sharma AK, Yadav RK, Ranjan A, et al. Evaluation of C4b as an adjunct marker in symptomatic RT-PCR negative Covid-19 cases. Indian J Clin Biochem. 2023;38(1):102–9. doi: 10.1007/s12291-022-01033-z 35756690 PMC9207170

[30] Guzzo KB, Hayford SR. Pathways to Parenthood in Social and Family Context: Decade in Review, 2020. J Marriage Fam. 2020;82(1):117–44. doi: 10.1111/jomf.12618 34012172 PMC8130890

[31] Ajzen I, Klobas J. Fertility intentions. DemRes. 2013;29:203–32. doi: 10.4054/demres.2013.29.8

[32] Khurana D, Koli A, Khatter K, Singh S. Natural language processing: state of the art, current trends and challenges. Multimed Tools Appl. 2023;82(3):3713–44. doi: 10.1007/s11042-022-13428-4 35855771 PMC9281254

[33] IBM. IBM Watson Natural Language Understanding: Chinese language support [Internet]. 2023. Available from: https://www.ibm.com/docs/en/watson-libraries?topic=natural-language-understanding-language-support

[34] Xu H, Liu B, Li P, Sun S. Constructing the affective lexicon ontology. J China Soc Sci Tech Inf. 2008;27(2):180–5.

[35] Li Z, Dai Y, Li X. Construction of sentimental knowledge graph of Chinese government policy comments. Knowledge Management Research & Practice. 2021;20(1):73–90. doi: 10.1080/14778238.2021.1971056

[36] Liu X, Wang S, Lu S, Yin Z, Li X, Yin L, et al. Adapting Feature Selection Algorithms for the Classification of Chinese Texts. Systems. 2023;11(9):483. doi: 10.3390/systems11090483

[37] Zhai C, Lafferty J. A study of smoothing methods for language models applied to Ad Hoc information retrieval. In: Proceedings of the 24th annual international ACM SIGIR conference on Research and development in information retrieval, 2001;334–42. doi: 10.1145/383952.384019

[38] Lin Y, Lin H, Xu K, Sun X. Learning to rank using smoothing methods for language modeling. J Am Soc Inf Sci Tec. 2013;64(4):818–28. doi: 10.1002/asi.22789

[39] Zhao J, Chen W, Liu Z, Liu W, Li K, Zhang B, et al. Urban expansion, economic development, and carbon emissions: Trends, patterns, and decoupling in mainland China’s provincial capitals (1985–2020). Ecological Indicators. 2024;169:112777. doi: 10.1016/j.ecolind.2024.112777

[40] Japaridze I, Sayour N. Housing Affordability Crisis and Delayed Fertility: Evidence from the USA. Popul Res Policy Rev. 2024;43(2). doi: 10.1007/s11113-024-09865-8

[41] Wang S, Wang Y, Shen Y. The Impact of Supportive Housing Policy Scenarios on Marriage and Fertility Intentions: A Vignette Survey Experimental Study in Shanghai, China. Popul Res Policy Rev. 2023;42(6). doi: 10.1007/s11113-023-09844-5

[42] Zou Y, Zhong N, Chen H, Pu C. Housing burden across Chinese cities: Spatio-temporal patterns and influential factors. Applied Geography. 2024;166:103259. doi: 10.1016/j.apgeog.2024.103259

[43] Yao P, Jia Q, Liu J, Yamaka W. Reform of Collective Land for Construction and Rental Housing and the Growth of Farmers’ Property Income: Evidence from China. Land. 2022;12(1):131. doi: 10.3390/land12010131

[44] China Daily. Report: Raising a child in China costs 6.3 times per capita GDP. 2024. Available from: https://www.chinadaily.com.cn/a/202402/21/WS65d5ee0ea31082fc043b85eb.html

[45] Lappegård T, Kristensen AP, Dommermuth L, Minello A, Vignoli D. The impact of narratives of the future on fertility intentions in Norway. J of Marriage and Family. 2022;84(2):476–93. doi: 10.1111/jomf.12822

[46] Novelli M, Cazzola A, Angeli A, Pasquini L. Fertility Intentions in Times of Rising Economic Uncertainty: Evidence from Italy from a Gender Perspective. Soc Indic Res. 2020;154(1):257–84. doi: 10.1007/s11205-020-02554-x

[47] Preis H, Tovim S, Mor P, Grisaru-Granovsky S, Samueloff A, Benyamini Y. Fertility intentions and the way they change following birth- a prospective longitudinal study. BMC Pregnancy Childbirth. 2020;20(1):228. doi: 10.1186/s12884-020-02922-y 32303192 PMC7164284

[48] Pezzulo C, Nilsen K, Carioli A, Tejedor-Garavito N, Hanspal SE, Hilber T, et al. Geographical distribution of fertility rates in 70 low-income, lower-middle-income, and upper-middle-income countries, 2010-16: a subnational analysis of cross-sectional surveys. Lancet Glob Health. 2021;9(6):e802–12. doi: 10.1016/S2214-109X(21)00082-6 34019836 PMC8149299

[49] Kim D, Jung H. The Long-Term Impact of College Education on Women’s Attitudes Toward Marriage and Children. Journal of Family Issues. 2023;45(1):103–23. doi: 10.1177/0192513x221150982

[50] Xiang Z, Zhang X, Li Y, Li J, Wang Y, Wang Y, et al. Fertility intention and its affecting factors in China: A national cross-sectional survey. Heliyon. 2023;9(2):e13445. doi: 10.1016/j.heliyon.2023.e13445 36814608 PMC9939585

[51] Yang H, Han R, Wang Z. Third-child fertility intention and its socioeconomic factors among women aged 20-34 years in China. BMC Public Health. 2023;23(1):821. doi: 10.1186/s12889-023-15719-3 37143051 PMC10161537

[52] Götmark F, Andersson M. Human fertility in relation to education, economy, religion, contraception, and family planning programs. BMC Public Health. 2020;20(1):265. doi: 10.1186/s12889-020-8331-7 32087705 PMC7036237

[53] Organisation for Economic Co-operation and Development OECD. Nordic early childhood education and care policies. OECD Family Database. 2023. https://www.oecd.org/els/family/database.htm

[54] National Health Commission of the People’s Republic of China. White paper on the development of child care services in China. 2023. http://en.nhc.gov.cn/2023-06/01/c_894879.htm

[55] Wang Z, Zhou W. Research on paternity leave for men. Popul Econ. 2022;(4). doi: 10.3969/j.issn.1000-4149.2022.04.008

[56] National Bureau of Statistics of China. 2018年全国时间利用调查公报. 2018 National Time Use Survey Bulletin. 2019. Available from: http://www.stats.gov.cn/sj/zxfb/202302/t20230203_1900224.html

[57] All-China Women’s Federation. Research report on the status of Chinese women in family childcare. Beijing: All-China Women’s Federation. 2022. http://www.women.org.cn/english/research/2022childcarereport.pdf

[58] Zhao S. Gender in Families: A Comparison of the Gendered Division of Child Care in Rural and Urban China. Child Youth Care Forum. 2020;49(4):511–31. doi: 10.1007/s10566-019-09541-5

[59] Hochschild AR, Machung A. The second shift: Working parents and the revolution at home. Rev ed ed. London: Penguin Books. 2012.

[60] Cukrowska-Torzewska E, Matysiak A. The motherhood wage penalty: A meta-analysis. Soc Sci Res. 2020;88–89:102416. doi: 10.1016/j.ssresearch.2020.102416 32469733

[61] Lesthaeghe R. The second demographic transition: a concise overview of its development. Proc Natl Acad Sci U S A. 2014;111(51):18112–5. doi: 10.1073/pnas.1420441111 25453112 PMC4280616

[62] Muñoz-Bullon F, Sanchez-Bueno MJ, Suárez-González I. Diversification decisions among family firms: The role of family involvement and generational stage. BRQ Business Research Quarterly. 2018;21(1):39–52. doi: 10.1016/j.brq.2017.11.001

[63] Hayfield N, Terry G, Clarke V, Ellis S. “Never Say Never?” Heterosexual, Bisexual, and Lesbian Women’s Accounts of Being Childfree. Psychology of Women Quarterly. 2019;43(4):526–38. doi: 10.1177/0361684319863414

[64] Schneider-Mayerson M, Leong KL. Eco-reproductive concerns in the age of climate change. Climatic Change. 2020;163(2):1007–23. doi: 10.1007/s10584-020-02923-y

[65] Dillarstone H, Brown LJ, Flores EC. Climate change, mental health, and reproductive decision-making: A systematic review. PLOS Clim. 2023;2(11):e0000236. doi: 10.1371/journal.pclm.0000236

