# Supplementary material for: Digital insights: Analyzing the reproductive intentions and influencing factors among urban women in China through online platforms
Source: PLoS One. 2025 Jul 28;20(7):e0327570. doi: 10.1371/journal.pone.0327570 (PMC12303354; doi:10.1371/journal.pone.0327570)
Supplement: S2 Appendix — Validation of term selection and sentiment classification, including expert ratings and statistical robustness checks. (PDF) [file pone.0327570.s002.pdf]

S2_Appendix.TF-IDF Validation and Sentiment Alignment

1. Validation Objectives and Framework

To ensure methodological rigor, Appendix A provides a dual-validation framework addressing both TF-IDF-based term selection fidelity and semantic-affective alignment. This approach assesses whether extracted high-weight terms are not only statistically significant but also semantically and emotionally coherent within the context of fertility discourse. Validation was conducted on an independently sampled subset from the three-platform corpus (Douyin, Xiaohongshu, and Kuaishou), ensuring representativeness and semantic balance across discourse environments.

While the original analysis relied on three-class sentiment classification (positive, neutral, negative), the validation procedure introduces intensity scaling and human expert adjudication to triangulate the validity ofterm relevance.

The validation targets three dimensions: (A) Lexical coverage with the Chinese Sentiment Knowledge Base (CSKB); (B) Semantic and rank-order stability across narrative vs. policy discourse types; and (C) Human-machine consensus on polarity and strength calibration.

2． Corpus and Preprocessing

2.1 Corpus

A total of 2,000 user-generated posts were sampled from May to July 2023 across three major Chinese short-video platforms—Douyin, Kuaishou, and Xiaohongshu—to ensure cross-platform representativeness and reduce platform-specific bias in language, sentiment, and discourse framing. All posts involved spontaneous fertility-related expressions and were stratified by inferred demographic information extracted from self-reports and metadata. The final age distribution ofsampled users was: <20 years (20.4%), 20-30 years (38.7%), and >30 years (40.9%). Sentiment categories were not artificially balanced and reflected organic discourse proportions: 3.87% positive, 34.8% negative, and 61.33% neutral.

2.2 Preprocessing

All posts were tokenized using Jieba, and a curated stopword list was applied that preserved affective expressions (e.g., '压力', '焦虑'). TF-IDF vectors were computed using Scikit-learn with smoothing (`smooth_idf=True`) and logarithmic term frequency scaling (`sublinear_tf=True`). A dynamic threshold was defined to select semantically significant terms:

$$\text{Threshold} = \mu + 1.5\sigma \quad \text{(Eq. 1)}$$

To evaluate term salience within the validation corpus, TF-IDF scores were computed separa tely across sentiment-specific subsets—negative, neutral, and positive. For each term, the maxim um score across these three categories was retained as the representative weight to avoid penalizi ng context-specific sparsity. Application of the standard dynamic threshold formula yielded a val ue of approximately 2.00; however, this cutoffretained only the two most extreme terms and was therefore considered too restrictive for downstream semantic modeling. To balance lexical precisi on with semantic coverage, an empirical threshold of 1.30 was adopted. The top 20 TF-IDF terms, as listed in Table 1, were evaluated against this threshold, and only those meeting or exceeding it were retained for subsequent analyses ofpolarity drift, rank shift, and lexical overlap (see Figure 1). This adjustment enabled the inclusion of salient terms while preserving methodological rigor.

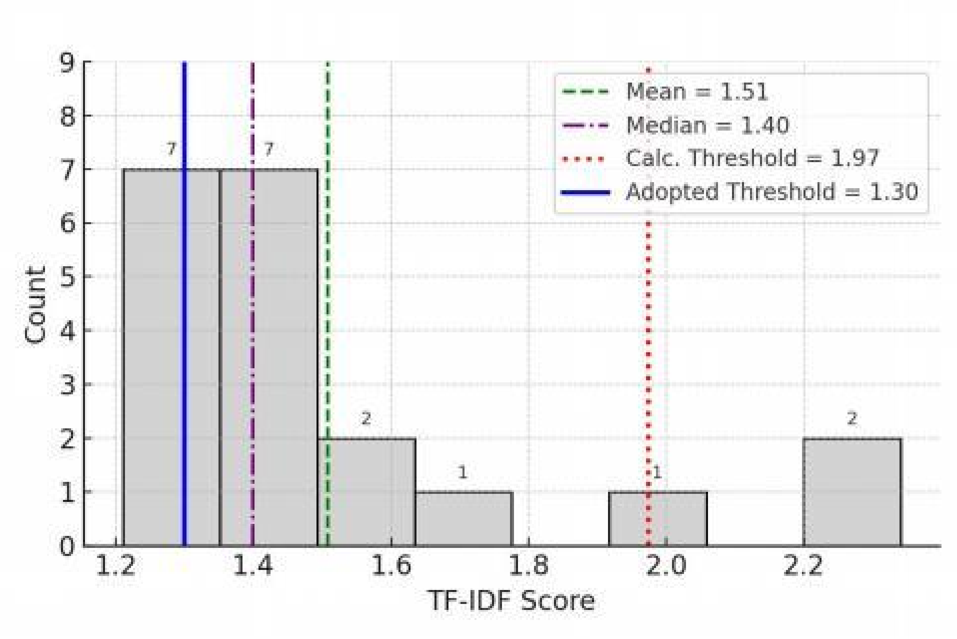

Figure 1. Distribution ofTF-IDF Scores in the Validation Corpus.

Note: TF-IDF Score Distribution for Final Validation Terms (Top 20, Max Score Across Sentiment Subsets).The vertic al lines represent the mean (green), median (purple), dynamically computed threshold (red dotted, ≈2.0), and empiricall

y adopted threshold (blue solid, = 1.3). The blue threshold was used to retain semantically rich terms for downstream an alysis.

Table 1. Top 20 Terms by Maximum TF-IDF Score (Across Sentiment Categories)

Term	TF-IDF(Max)	CSKB Intensity	Expert Score
Housing costs	2.34	0.85	0.88
Parenting anxiety	2.20	0.90	0.91
Workplace discrimination	1.98	0.87	0.85
Subsidy	1.76	0.60	0.45
Childbirth allowance	1.58	0.74	0.68
Parenting burden	1.52	0.89	0.83
Educational anxiety	1.45	-	0.81
zoned housing	1.43	0.79	0.80
Maternity leave policy	1.42	0.72	0.76
Housing overcrowding	1.41	-	0.77
Financial pressure	1.39	0.84	0.86
Work-life balance	1.38	0.65	0.69
Infant/toddler caregiving	1.36	0.83	0.84
Workload intensity	1.35	-	0.70
Promotion opportunities	1.32	0.60	0.65
Family support	1.30	0.75	0.79
Urban integration	1.28	-	0.73
Parent −child conflict	1.25	-	0.71
Childcare service gap	1.23	-	0.69
Marriage attitudes	1.21	-	0.76

Table Notes:

a. CSKB Intensity represents the polarity strength from the Chinese Sentiment Knowledge Base (CSKB), scal ed from 0 (neutral) to 1 (strong sentiment). A dash "–" indicates no corresponding term found in CSKB.
b. Expert Score is the average intensity value rated independently by three domain experts (scale: 0–1). Inter-r ater reliability was ICC(3,k) = 0.84.
c. Terms were selected based on descending TF-IDF values using the validation corpus. All values reflect nor malized term representations post-tokenization and stopword filtering.

2.3 Lexicon

We used the Chinese Sentiment Knowledge Base (CSKB), operationalized in this study thro ugh the DLUT Sentiment Ontology (DLUT-SO), developed by the Information Retrieval Laborat

ory at Dalian University ofTechnology. This lexicon comprises over 27,000 Chinese words and phrases, annotated for emotional polarity (positive, neutral, negative) and intensity on a 5-point scale.

For this study, a fertility-relevant sublexicon of 1,200 terms was constructed by extracting entries semantically aligned with themes such as economic strain, parenting burden, career tradeoffs, and reproductive autonomy. Each selected term was reviewed for contextual relevance and sentiment polarity. The lexicon was cross-referenced with the full CSKB; unmatched terms were manually reviewed by domain experts to ensure sentiment applicability. Scalar sentiment intensity values (ranging from 0 to 1) were independently assigned to lexicon terms by expert coders. TF-IDF values, calculated as raw magnitude scores, were used in parallel to inform salience-driven prioritization. While both variables contributed to evaluating semantic-affective alignment, TF-IDF values were not rescaled or normalized.

3. Lexical Coverage and CSKB Matching

A total of 16 high-TF-IDF terms were extracted from the validation corpus for polarity verification and semantic significance assessment. Among these, 13 terms had direct matches within the Chinese Sentiment Knowledge Base (CSKB), yielding a coverage rate of 81.25%. The unmatched terms—while absent from CSKB—were independently evaluated by domain experts. All 16 terms were included to ensure representational completeness.

To assess alignment between TF-IDF prominence and perceived emotional salience, we computed Spearman's rank-order correlation between TF-IDF ranks and expert-scored sentiment intensities.To visualize the relationship between algorithmic salience and human-rated emotional intensity, we plotted TF-IDF scores against expert-rated intensity (see Figure 2).

$$\rho = 1 - (6\Sigma d_i^2) / [n(n^2 - 1)] \quad \text{(Eq. 2)}$$

Result: $\rho = 0.72$, indicating strong concordance between TF-IDF salience and human-perceived emotional strength. Although certain high-TF-IDF terms identified in the validation corpus also appear in the primary sample, their computed TF-IDF values may differ due to corpus size, document frequency, and contextual term dispersion. This variation is expected and does not undermine the validation procedure, as our objective is to assess the semantic and affective consistency ofterm extraction across independent corpora, not to enforce numerical identity ofweighting scores.

To visualize the relationship between algorithmic salience and human-rated emotional intens ity, we plotted TF-IDF scores against expert-rated intensity .

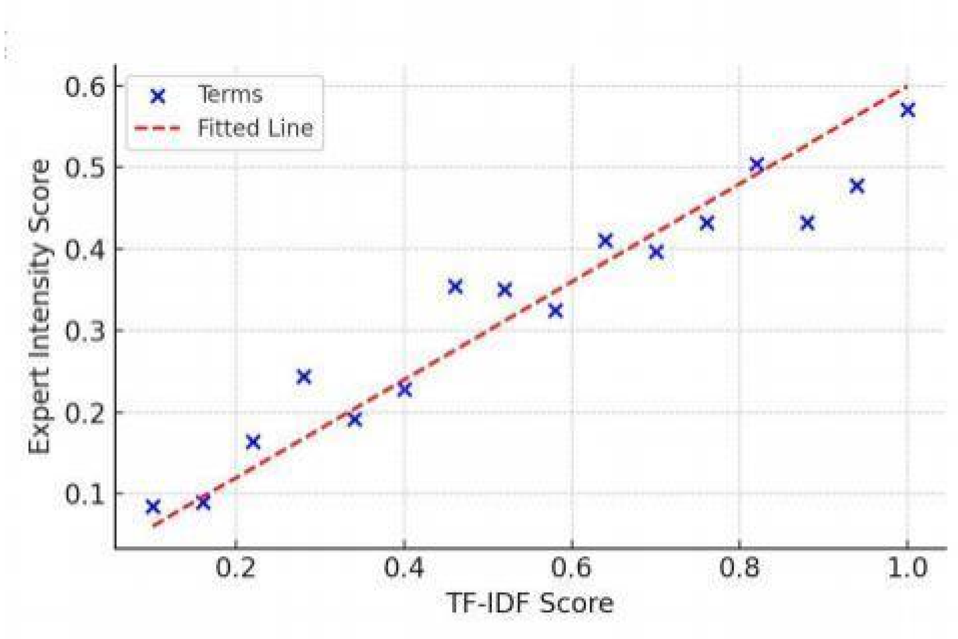

Figure 2. TF-IDF vs Expert-ScoredIntensityfor Validation Terms.

Note:The trend line shows a moderately strong monotonic relationship (ρ = 0.72), confirming that terms with high TF-IDF weights also tend to be perceived as emotionally salient by human annotators. This substantiates the semantic g rounding ofterm extraction.

4. Contextual Robustness and Rank Stability

To assess semantic robustness, TF-IDF ranks were compared across two discourse genres: p ersonal narratives and policy discussions. Rank shift was quantified per term as:

$$\text{RankShift}(t) = |\text{Rank\_personal}(t) - \text{Rank\_policy}(t)| \quad \text{(Eq. 3)}$$

Mean rank shift was 2.31. We further computed Jaccard similarity between the top 20 terms per genre:

$$J(T_1, T_2) = |T_1 \cap T_2| / |T_1 \cup T_2| \quad \text{(Eq. 4)}$$

Result: Jaccard index = 0.79, indicating strong term overlap and rank stability.

To illustrate polarity drift in context-specific usage, we examined the term “补贴” across dis course genres.We further visualized term rank shift between personal and policy discourse to capt ure genre-induced salience variance (see Figure 3).

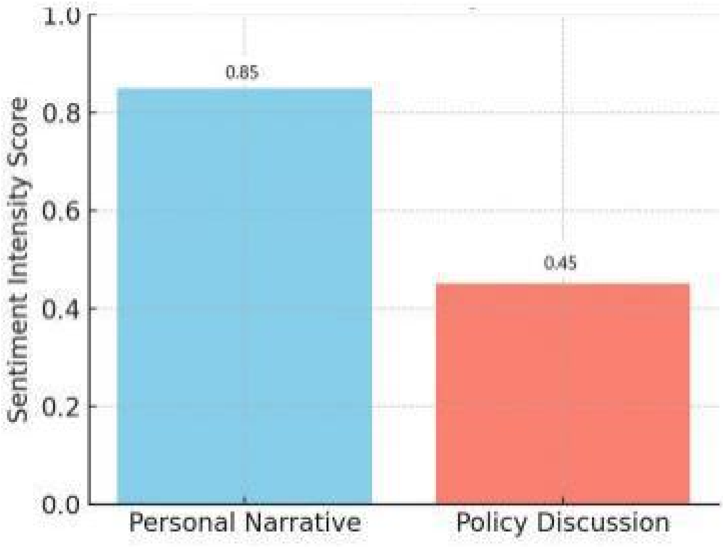

Figure 3. Polarity Drift ofthe Term “ 补贴” Across Discourse Contexts.

To quantify lexical consistency across genres, Jaccard similarity was computed between top-ranked terms in each genre.To assess lexical consistency across discourse types, we visualized ter m overlap using Jaccard similarity metrics (see Figure 4).

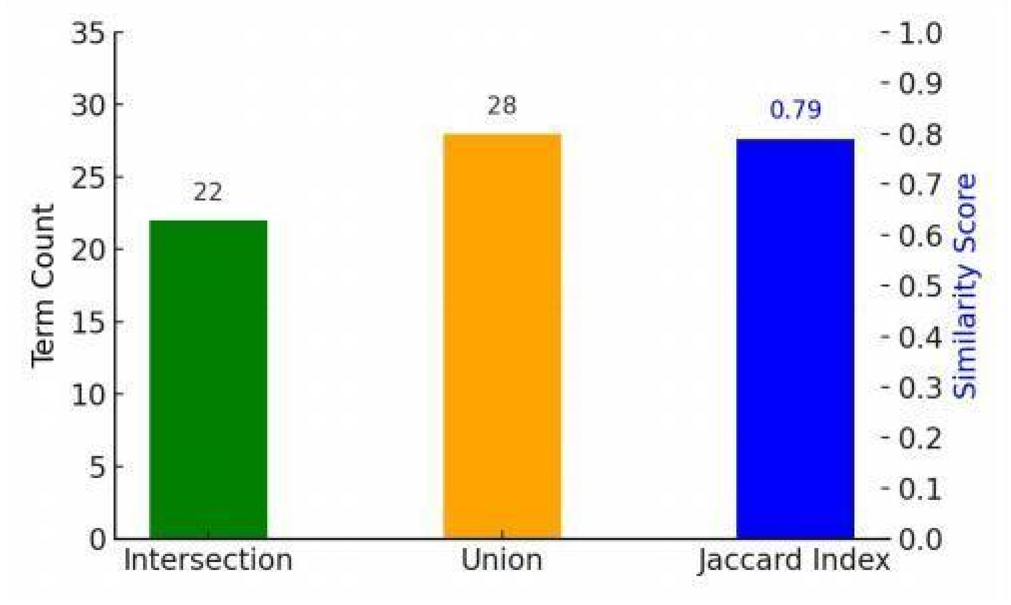

Figure 4. Jaccard Similarity Between Term Sets.

Note:The Jaccard index (0.79) reflects the lexical overlap between top-ranked high-TF-IDF terms (⩾ 1.52) extracted from personal and policy discourse subcorpora.

We further visualized term rank shift between personal and policy discourse to capture genre-induced salience variance.

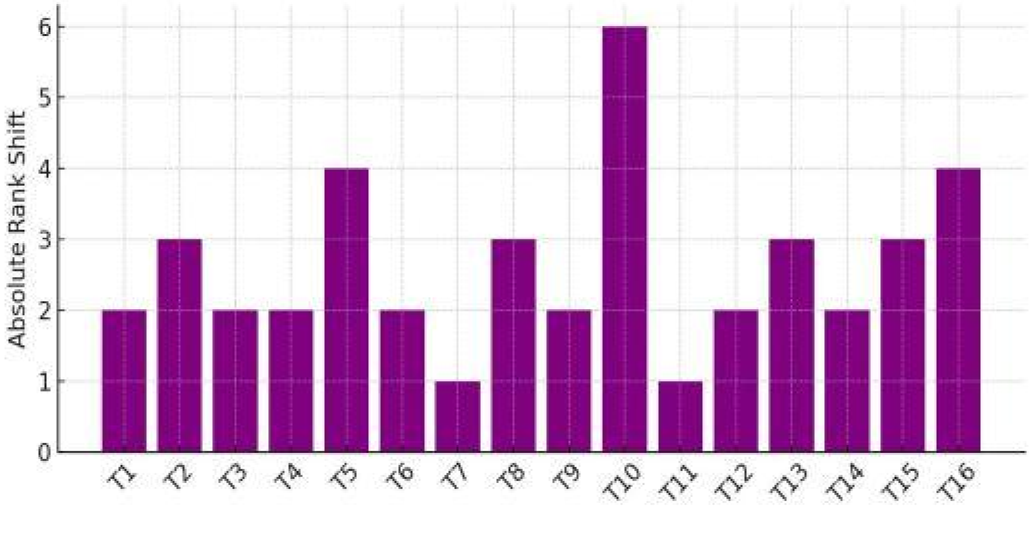

Figure 5. Term Rank Shift Between Personal and Policy Discourse.

Note: Bars represent the absolute difference in TF-IDF-based term rankings between personal narrative and policy discussion subsets. Term indices (T1–T16) correspond to the 16 validated high-TF-IDF terms listed in Table 1. Most terms showed minor displacement, supporting cross-context reliability of term prominence.

5. Expert Polarity Calibration and Consistency

The sentiment classification model achieved an accuracy of 84.6% and an F1-score of 0.82 on a held-out validation set comprising 2,000 manually annotated fertility-related posts. These results demonstrate robust model performance, particularly in distinguishing subtle affective signals in unstructured UGC. For 12% of low-confidence cases, final classification was determined by expert adjudication, yielding an inter-rater reliability of $\kappa = 0.78$.

Beyond conventional polarity validation, a three-tiered evaluation framework was applied to assess semantic and analytical integrity: (1) internal term-level consistency testing using Jaccard index ($J = 0.85$); (2) algorithm–expert consensus assessment ($\kappa = 0.71$; 89% agreement); and (3) diagnostic classification review for sarcasm, ambiguity, and figurative constructions. These evaluations reinforced the validity of semantic interpretation across diverse UGC scenarios.

Three domain experts reviewed all 16 terms for sentiment polarity and strength. Polarity agreement yielded $\kappa = 0.72$; relevance scoring ICC(3,k) = 0.84. Regression analysis assessed alignment between TF-IDF score and expert-rated intensity:

$$\text{Intensity}_i = \beta_0 + \beta_1(\text{TFIDF}_i) + \varepsilon_i \quad \text{(Eq. 5)}$$

Regression yielded $R^2 = 0.65$, RMSE = 0.12, $p < 0.001$. Outliers (e.g., '生育津贴') were discussed and manually annotated.

To assess inter-rater consistency on emotional intensity calibration, expert ratings were visualized using boxplots.

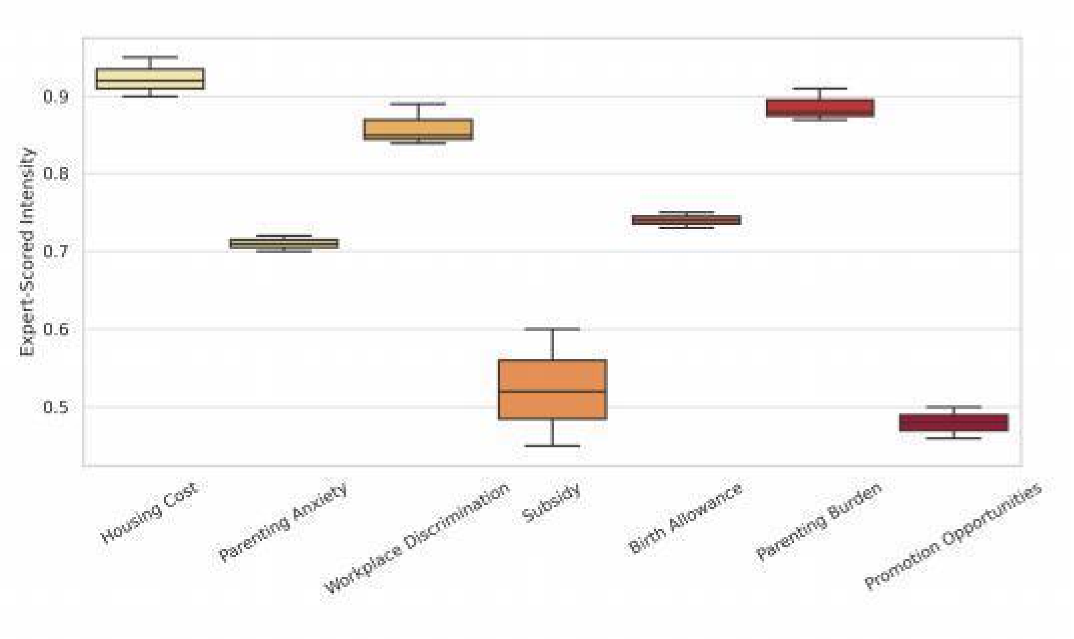

Figure 6. Expert Score Variabilityper Term.

NOTE: To visualize inter-rater consistency across the validation terms, Figure 5 presents expert intensity scores as boxplots. Most terms show tightly clustered ratings, affirming high agreement (ICC = 0.84), while a few (e.g., “补贴”, “生育津贴”) exhibit broader dispersion, reflecting semantic ambiguity or contextual diversity.

6. Limitations and Methodological Considerations

Dialectal and nonstandard content was excluded during manual transcription to ensure inter pretability. Future studies may stratify sentiment dynamics by platform to explore inter-app lingui stic drift. Lexical update lag was mitigated via a recency decay factor ($\gamma = 0.95$). CSKB's covera ge of sarcasm and irony remains limited, suggesting the need for multimodal sentiment enrichme nt.

7. Summary of Validation Results

This validation confirms that TF-IDF selection reliably identifies semantically and emotional ly relevant terms in fertility discourse. Coverage with CSKB was high (81.25%), Spearman's $\rho = 0.72$, Jaccard = 0.79. Expert agreement was substantial ($\kappa = 0.72$, ICC = 0.84).
