# Supplementary material for: Digital insights: Analyzing the reproductive intentions and influencing factors among urban women in China through online platforms
Source: PLoS One. 2025 Jul 28;20(7):e0327570. doi: 10.1371/journal.pone.0327570 (PMC12303354; doi:10.1371/journal.pone.0327570)
Supplement: S3 Appendix — Detailed descriptions and sentiment labels of external factors influencing fertility intentions, grouped by thematic category. (PDF) [file pone.0327570.s003.pdf]

S3_Appendix.Definitions of External Factors

This appendix provides **comprehensive definitions** and **contextual explana tions** of the external factors identified in the quantitative analysis of fertility in tentions. These factors are grouped into four categories: **Socioeconomic Status**, **Social Support**, **Social Psychological Factors**, and **Socio-Cultural Factors**. E ach factor is explained in detail, along with its corresponding sentiment classifi cation (negative, neutral, and positive), based on user-generated content. These definitions are intended to help the reader understand the multifaceted impact o f these factors on fertility intentions.

1. Socioeconomic Status

1 Urban Mobility

Definition:Urban mobility refers to the ability of individuals to move thro ugh urban environments efficiently, encompassing the accessibility of public tra nsportation, road infrastructure, and the overall convenience of commuting withi n the city. This mobility impacts how individuals access jobs, healthcare, educa tion, and social services. For women, in particular, urban mobility can also infl uence how easily they can balance domestic responsibilities with professional c ommitments, especially when they are caregivers or need to manage household duties. Limited urban mobility can increase feelings of frustration and stress, particularly when individuals feel trapped in an area with limited resources, po or infrastructure, or unaffordable housing, which can lead to delays in family p lanning decisions.

When individuals face long commutes, limited transportation options, or traffic congestion, it can exacerbate feelings of time scarcity and exhaustion. These pressures are especially felt by women, who may already experience the dual burden of domestic and professional roles. The resulting anxiety can discourage people from expanding their families, as they may feel they cannot provide a stable or comfortable environment for children.

Example: "I spend over two hours a day commuting, and the thought of having to take care of kids on top of my job and long commute makes me unsure about having children in the near future."

2 **Housing Cost**

Definition:Housing cost refers to the financial strain caused by acquiring, renting, or maintaining a home, particularly in urban areas where real estate prices and rent tend to be much higher. For many individuals, especially young couples, securing stable housing is a significant financial hurdle. The high cost of housing can act as a strong deterrent to having children, as individuals or couples may feel that they cannot afford both a home and the additional expenses that come with raising children. This economic pressure is even more pronounced for women, who often face challenges in securing job stability, earning potential, and managing childcare expenses.

The inability to afford suitable housing or the overwhelming cost of living in a major city can cause stress and uncertainty about the future, leading to the postponement of fertility decisions. When individuals feel like their living situation is insecure, they may hesitate to take on the financial commitment that childbearing requires.

Example: "The rising cost of rent makes it impossible for us to save for a house. We're already stretched thin financially, and having children just feels like an unrealistic goal at this point."

3 **Child-Rearing Expenses**

Definition:Child-rearing expenses refer to the financial costs associated with raising children, including education, healthcare, childcare, and general living expenses. These costs can place a significant burden on parents, especially in urban environments where the cost of living is high. Childcare, schooling, and healthcare can be especially expensive, particularly for families who do not have sufficient support or resources to manage these costs. The high cost of raising children can often deter individuals from having more children, as they may feel financially unprepared or overwhelmed.

For women, particularly, child-rearing expenses are often seen as an additional strain due to wage gaps, career interruptions, and the high cost of maternal care. These financial pressures can lead to delays in having children or the decision to limit family size.

Example: "The thought of paying for daycare and sending my kids to school is already overwhelming. We're struggling to afford these costs, and having another child would just add to the financial burden."

4 Adaptation of Urban Newcomers

Definition:The adaptation of urban newcomers refers to the process of adjusting to the fast-paced and often stressful environment of a new city, particularly for individuals who have relocated from smaller towns or rural areas. This adaptation can include overcoming challenges such as finding affordable housing, securing stable employment, adjusting to a higher cost of living, and integrating into a new social environment. For women, adapting to city life can be particularly challenging due to the increased expectations placed on them in terms of caregiving, work-life balance, and career progression.

The stress of adapting to a new urban environment can make individuals feel unstable and unsure about the future, particularly when faced with the high cost of living or social isolation. This uncertainty often leads to delays in family planning, as individuals may not feel confident about their ability to provide a stable and nurturing environment for children.

Example: "I moved to this city for a job, but I feel overwhelmed by the fast pace of life, the high rent, and the lack of a social support system. I don't feel ready to have children in this uncertain environment."

2. Social Support

1 Maternal Stress

Definition:Maternal stress refers to the emotional, psychological, and physical strain that mothers experience due to the demands of motherhood, including caregiving, managing household chores, and fulfilling work obligations. Societal expectations often place the primary responsibility for childcare on women, leading to an imbalance in household duties and professional commitments. This strain is exacerbated by the lack of adequate support systems, such as affordable childcare, flexible work schedules, and access to healthcare. When women are unable to find adequate support, the stress of managing multiple roles can lead to burnout and frustration, making the decision to have more children difficult or impossible.

This stress is compounded by gendered expectations around parenting and caregiving, where women are often expected to be the primary caregivers while also managing careers. The societal pressure to excel in both roles without sufficient assistance can cause significant anxiety, resulting in delayed or avoided childbearing.

Example: "Between work, household chores, and taking care of my kids, I never have time for myself. I'm exhausted, and the idea of adding another child to the mix just seems overwhelming."

2 Workplace Pregnancy Equity

Definition:Workplace pregnancy equity refers to the degree to which women receive fair treatment regarding maternity leave, job security, and career pro

gression when they become pregnant. In many workplaces, pregnancy is still seen as a disruption to an employee's productivity, and women often face discrimination or a lack of support during and after maternity leave. This inequity can lead to significant anxiety and stress, as women may fear that having children will hurt their careers. These workplace pressures can result in delays in childbearing, as women may prioritize job security and career advancement over starting a family.

Example: "My workplace doesn't provide paid maternity leave, and I fear that taking time off will hurt my career. I'm hesitant to have children because I don't want to risk my job stability."

3 **Childcare Services**

Definition:Childcare services refer to the availability, accessibility, and affordability of services that provide care for children, including daycare centers, after-school programs, and nannies. The availability of quality childcare is a significant factor in fertility intentions, particularly for working parents. When childcare services are too expensive, unavailable, or of low quality, it places a heavy burden on parents, particularly mothers, who must balance professional responsibilities with caregiving. The lack of reliable childcare can discourage women from having children or limit family size, as it becomes increasingly difficult to manage both work and family responsibilities.

Example: "Childcare costs are so high that I can't afford to go back to work. Without affordable options, it feels impossible to balance having children with maintaining a career."

4 **Career Advancement and Personal Goals**

Definition:Career advancement and personal goals refer to the aspirations individuals have regarding their professional development, personal growth, and the pursuit of personal achievements. For many, the desire to progress in their careers or focus on personal goals often conflicts with the demands of childb

earing. Women, in particular, may feel the pressure of career stagnation if they take time off to have children. The desire to achieve financial security or career success often leads individuals to delay childbearing, as they prioritize achieving personal milestones before starting a family.

Example: "I want to finish my degree and establish my career before I have children. Right now, it feels like I can't manage both career advancement and motherhood."

3. Social Psychological Factors

1 Childbirth Care

Definition:Childbirth care refers to the medical and emotional support provided to women during pregnancy, childbirth, and the postpartum period. The quality of care, the availability of healthcare professionals, and the support of family or friends can significantly influence an individual's fertility decisions. Positive experiences with childbirth care, such as receiving appropriate medical attention and emotional support, can encourage individuals to have more children, while negative experiences can deter individuals from childbearing.

Example: "I had a great experience at the hospital, where the staff was very supportive, and everything went smoothly. This has made me more confident about having another child in the future."

② Pregnancy-Related Health Concerns

Definition:Pregnancy-related health concerns encompass any medical complications during pregnancy, such as gestational diabetes, hypertension, or fetal development issues. These complications can lead to anxiety about future pregnancies, prompting some women to delay or avoid having children due to perceived health risks.Online platforms like Douyin (TikTok) can exacerbate these concerns. For example, non-medical influencers may exaggerate conditions like pubic symphysis dysfunction, describing it as a pelvic fracture that cannot heal nat

urally. Such misinformation significantly heightens fear, particularly among unm arried or childless women, impacting their decisions regarding pregnancy. This type of content amplifies psychological distress and can lead to a reluctance to have children due to exaggerated health fears.

Example:"After my difficult pregnancy, I'm worried about the health risks involved in having another child. The complications I faced have made me h esitant to consider pregnancy again."

3 Work-Life Balance

Definition:Work-life balance refers to the ability of individuals to effective ly manage their professional and personal lives. Achieving a healthy work-life balance is particularly challenging for women, who often juggle multiple roles, including professional responsibilities, childcare, and household duties. When work-life balance is disrupted, it can lead to stress, burnout, and a reduction in fertility intentions. Women who feel they cannot balance career and family m ay delay or avoid having children altogether.

Example:"I'm working long hours and taking care of everything at home. It's just too much, and I'm not sure when I'll ever have the time to have an other child."

4 Psychosocial Adjustment Issues

Definition:Psychosocial adjustment issues refer to the emotional and psych ological challenges individuals face when adjusting to the roles and responsibili ties of parenthood. These issues can include the emotional toll of changing ide ntity from an individual to a parent, the stress of balancing multiple roles, and the adjustment to societal expectations. Difficulty in adjusting to these change s can influence fertility decisions, as individuals may feel overwhelmed or uns ure about the future.

Example:"Adjusting to life as a parent has been harder than I thought, an d I'm still struggling to find a balance. I'm not sure I'm ready to have anothe r child with all the stress I'm dealing with."

4. Socio-Cultural Factors

1 DINK (Dual Income No Kids)

Definition:DINK (Dual Income, No Kids) refers to a lifestyle choice made by couples who decide not to have children, often to prioritize career advance ment, financial independence, personal freedom, or lifestyle choices such as tra vel. The growing trend of DINKs reflects broader societal shifts where individ uals and couples are more focused on self-fulfillment, professional growth, and financial stability than traditional family roles. This lifestyle choice is increasi ngly seen as a valid and modern personal decision, though it can still be view ed critically in some cultures that emphasize traditional family structures.

Example: "My partner and I are both focusing on our careers right now, and we're not ready to give up our freedom for children. We enjoy our lifesty le and want to continue traveling and focusing on personal goals."

② Diverse Sexual Orientations (e.g., Lesbian)

Definition: This refers to the recognition and inclusion of individuals with diverse sexual orientations, including same-sex couples and those in non-hetero normative relationships. Fertility decisions within the LGBTQ+ community may be influenced by social, cultural, and legal factors such as access to fertility t reatments, legal recognition of relationships, societal acceptance, and the availab ility of support systems. Same-sex couples may also face unique challenges in terms of family-building, such as societal discrimination, the lack of inclusive healthcare options, and access to legal recognition of parental rights.

Example: "As a lesbian couple, we face challenges in accessing fertility tr eatments, but we feel encouraged by the growing inclusivity of healthcare prov

iders and the supportive legal framework. We’re hopeful for the future, though it’s still an uphill battle."

3 Deinstitutionalization of Marriage

Definition: Deinstitutionalization of marriage refers to the gradual shift in s ocietal views, where marriage is no longer seen as a mandatory institution for childbearing or family formation. Over recent years, especially among women, there has been a growing tendency to delay or even forgo marriage entirely, of ten in favor of career, personal growth, or financial stability. This shift reflects a change in societal norms, where personal freedom and choice in family pla nning are prioritized over traditional expectations.

Many women today no longer feel that marriage is necessary to have chil dren. This change is driven by multiple factors: some women view marriage as an outdated institution that imposes societal and gendered expectations of care giving, household responsibilities, and economic dependency. For these women, having children without marriage—sometimes through short-term or non-commi tted relationships—has become more common, as they seek to avoid the respon sibilities and constraints associated with marriage. This trend is sometimes refer red to as the “leave the father, keep the child” phenomenon, where the focus i s on motherhood rather than traditional family structures.

At the same time, many women continue to value love and commitment, but no longer see marriage as the necessary formality to validate these qualitie s. They increasingly see marriage as just another possible relationship arrangem ent, not an essential component of family life. This shift in attitudes has empo wered women to make independent choices about when and how to have child ren, without the pressure to marry first.

Example: "I don’t think marriage should be a prerequisite for having chil dren. We’re in a strong relationship, and we’re ready to start a family, even if we aren’t married yet."

4 Social and Environmental Change

Definition: Social and environmental change refers to the broader shifts in society and the environment that influence fertility intentions. Within this study, these changes include concerns about climate change, environmental degradation, economic uncertainty, social unrest, and shifting cultural norms. For many indi viduals, the future stability of the planet and society plays a key role in decisi ons regarding childbearing. These social and environmental concerns may lead some to delay or reconsider having children, as individuals may feel uncertain or pessimistic about the world they would be bringing children into.

Example: "With the world facing climate change and economic uncertainty, I'm hesitant to bring a child into this world. I'm unsure about what the futur e holds, and I don't want to burden my child with the challenges ahead."
