# Supplementary material for: Digital insights: Analyzing the reproductive intentions and influencing factors among urban women in China through online platforms
Source: PLoS One. 2025 Jul 28;20(7):e0327570. doi: 10.1371/journal.pone.0327570 (PMC12303354; doi:10.1371/journal.pone.0327570)
Supplement: Attachment — Submitted filename: PONE-D-24-26854.pdf [file pone.0327570.s004.pdf]

PLOS ONE

Digital Insights: Exploring Women's Reproductive Intentions and Influencing Factors in the Online Sphere

--Manuscript Draft--

**Manuscript Number:**	PONE-D-24-26854
**Article Type:**	Research Article
**Full Title:**	Digital Insights: Exploring Women's Reproductive Intentions and Influencing Factors in the Online Sphere
**Short Title:**	Digital Insights: Women's Reproductive Intentions Online
**Corresponding Author:**	Xuan Sun, Ph.D<br>Nankai University<br>Tianjin, CHINA
**Keywords:**	Fertility Intentions, Online Platforms, Reproductive Female, TikTok, Xiaohongshu, Kuaishou
**Abstract:**	Objectives:This study aims to provide profound insights into contemporary women's reproductive intentions and identify the factors influencing their fertility decisions by leveraging data from high-traffic websites.<br>Methods: Three prominent online platforms, namely TikTok, Xiaohongshu, and Kuaishou, were selected as sources. Data spanning from May 2021 to April 2024 were systematically collected using keyword search and content analysis methods. Rigorous data cleaning and classification procedures were employed to ensure the validity and accuracy of the data.<br>Results: High-frequency keyword analysis and binary regression techniques uncovered several pivotal factors influencing fertility intentions among online users. Significant socioeconomic determinants encompassed urban mobility (16.8%), housing pressures (25.1%), and the cost of raising children (13.1%). Maternal stress (2.6%) and concerns over workplace pregnancy discrimination (13.0%) also emerged as crucial influencers. Anxiety related to childbirth (4.0%), such as issues with compassionate care during delivery and pregnancy-related body anxieties, significantly impacted fertility decisions. Cultural beliefs (5.0%), including preferences like DINK and lesbian (2.3%), further influenced reproductive intentions. Binary logistic regression analysis highlighted that individuals with a bachelor's degree (B = -0.54, 95% CI [0.552, 5.34], $p < 0.05$) and those with higher education levels (B = -1.282, 95% CI [0.295, 1.932], $p < 0.05$) exhibited lower fertility intentions. Data analysis from 2022 (B = -2.654, 95% CI [0.026, 0.189], $p < 0.001$) and 2023 (B = -3.923, 95% CI [4.437, 6.005], $p < 0.001$) indicated significant deviations from 2021. Furthermore, residents of first-tier cities showed notable variations (B = -3.498, 95% CI [0.001, 0.659], $p < 0.05$). Socioeconomic factors, social support, and fertility-related anxiety/fear exhibited a significant negative correlation with fertility intention.<br>Conclusion: This study identifies socioeconomic conditions and support structures as pivotal determinants influencing fertility intentions within distinct social groups. Additionally, it underscores the significance of cultural beliefs and anxieties related to fertility. These findings are crucial for policymakers aiming to foster healthy attitudes toward marriage and fertility, thereby supporting societal development positively.
**Order of Authors:**	Jing Xiang, M.D.
	Xuan Sun
**Additional Information:**	
**Question**	**Response**
**Financial Disclosure**<br><br>Enter a financial disclosure statement that describes the sources of funding for the work included in this submission. Review	Yes

Powered by Editorial Manager® and ProduXion Manager® from Aries Systems Corporation

the submission guidelines for detailed requirements. View published research articles from *PLOS ONE* for specific examples.<br><br>This statement is required for submission and **will appear in the published article** if the submission is accepted. Please make sure it is accurate.<br><br>**Funded studies**<br>Enter a statement with the following details:<br>• Initials of the authors who received each award<br>• Grant numbers awarded to each author<br>• The full name of each funder<br>• URL of each funder website<br>• Did the sponsors or funders play any role in the study design, data collection and analysis, decision to publish, or preparation of the manuscript?<br><br>Did you receive funding for this work?	
Please add funding details.<br>as follow-up to "**Financial Disclosure**<br><br>Enter a financial disclosure statement that describes the sources of funding for the work included in this submission. Review the submission guidelines for detailed requirements. View published research articles from *PLOS ONE* for specific examples.<br><br>This statement is required for submission and **will appear in the published article** if the submission is accepted. Please make sure it is accurate.	Youth Project of the Humanities and Social Sciences Fund of the Ministry of Education, Research on the Social Coordinated Mechanism of Medical-Patient Community Construction (19YJC840030).

**Funded studies**<br>Enter a statement with the following details:<br>• Initials of the authors who received each award<br>• Grant numbers awarded to each author<br>• The full name of each funder<br>• URL of each funder website<br>• Did the sponsors or funders play any role in the study design, data collection and analysis, decision to publish, or preparation of the manuscript?<br><br>Did you receive funding for this work?"	
Please select the country of your main research funder (please select carefully as in some cases this is used in fee calculation).<br>as follow-up to "**Financial Disclosure**<br><br>Enter a financial disclosure statement that describes the sources of funding for the work included in this submission. Review the submission guidelines for detailed requirements. View published research articles from *PLOS ONE* for specific examples.<br><br>This statement is required for submission and **will appear in the published article** if the submission is accepted. Please make sure it is accurate.<br><br>**Funded studies**<br>Enter a statement with the following details:<br>• Initials of the authors who received each award<br>• Grant numbers awarded to each author<br>• The full name of each funder<br>• URL of each funder website<br>• Did the sponsors or funders play any role in the study design, data collection and analysis, decision to publish, or preparation of the manuscript?<br><br>Did you receive funding for this work?"	CHINA - CN
**Competing Interests**	The authors have declared that no competing interests exist.

Use the instructions below to enter a competing interest statement for this submission. On behalf of all authors, disclose any competing interests that could be perceived to bias this work—acknowledging all financial support and any other relevant financial or non-financial competing interests.<br><br>This statement is required for submission and **will appear in the published article** if the submission is accepted. Please make sure it is accurate and that any funding sources listed in your Funding Information later in the submission form are also declared in your Financial Disclosure statement.<br><br>View published research articles from *PLOS ONE* for specific examples.<br><br>**NO authors have competing interests**<br><br>Enter: *The authors have declared that no competing interests exist.*<br><br>**Authors with competing interests**<br><br>Enter competing interest details beginning with this statement:<br><br>*I have read the journal's policy and the authors of this manuscript have the following competing interests: [insert competing interests here]*<br><br>* typeset	
**Ethics Statement**<br><br>Enter an ethics statement for this submission. This statement is required if the study involved:<br><br>• Human participants<br>• Human specimens or tissue<br>• Vertebrate animals or cephalopods<br>• Vertebrate embryos or tissues	N/A

• Field research<br><br>Write "N/A" if the submission does not require an ethics statement.<br><br>General guidance is provided below. Consult the submission guidelines for detailed instructions. **Make sure that all information entered here is included in the Methods section of the manuscript.**	

Powered by Editorial Manager® and ProduXion Manager® from Aries Systems Corporation

**Format for specific study types**<br><br>**Human Subject Research (involving human participants and/or tissue)**<br>• Give the name of the institutional review board or ethics committee that approved the study<br>• Include the approval number and/or a statement indicating approval of this research<br>• Indicate the form of consent obtained (written/oral) or the reason that consent was not obtained (e.g. the data were analyzed anonymously)<br><br>**Animal Research (involving vertebrate animals, embryos or tissues)**<br>• Provide the name of the Institutional Animal Care and Use Committee (IACUC) or other relevant ethics board that reviewed the study protocol, and indicate whether they approved this research or granted a formal waiver of ethical approval<br>• Include an approval number if one was obtained<br>• If the study involved *non-human primates*, add *additional details* about animal welfare and steps taken to ameliorate suffering<br>• If anesthesia, euthanasia, or any kind of animal sacrifice is part of the study, include briefly which substances and/or methods were applied<br><br>**Field Research**<br><br>Include the following details if this study involves the collection of plant, animal, or other materials from a natural setting:<br>• Field permit number<br>• Name of the institution or relevant body that granted permission	
**Data Availability**<br><br>Authors are required to make all data underlying the findings described fully available, without restriction, and from the time of publication. PLOS allows rare exceptions to address legal and ethical concerns. See the PLOS Data Policy and FAQ for detailed information.	No - some restrictions will apply

A Data Availability Statement describing where the data can be found is required at submission. Your answers to this question constitute the Data Availability Statement and **will be published in the article**, if accepted.<br><br>**Important:** Stating 'data available on request from the author' is not sufficient. If your data are only available upon request, select 'No' for the first question and explain your exceptional situation in the text box.<br><br>Do the authors confirm that all data underlying the findings described in their manuscript are fully available without restriction?	
**Describe where the data may be found in full sentences. If you are copying our sample text, replace any instances of XXX with the appropriate details.**<br><br>• If the data are **held or will be held in a public repository**, include URLs, accession numbers or DOIs. If this information will only be available after acceptance, indicate this by ticking the box below. For example: *All XXX files are available from the XXX database (accession number(s) XXX, XXX.).*<br>• If the data are all contained **within the manuscript and/or Supporting Information files**, enter the following: *All relevant data are within the manuscript and its Supporting Information files.*<br>• If neither of these applies but you are able to provide **details of access elsewhere**, with or without limitations, please do so. For example:<br><br>*Data cannot be shared publicly because of [XXX]. Data are available from the XXX Institutional Data Access / Ethics Committee (contact via XXX) for researchers who meet the criteria for access to confidential data.*<br><br>*The data underlying the results presented in the study are available from (include the name of the third party*	All original data from the article can be publicly obtained by contacting the corresponding author.

and contact information or URL).<br>• This text is appropriate if the data are owned by a third party and authors do not have permission to share the data.<br><br>* typeset	
Additional data availability information:	

Powered by Editorial Manager® and ProduXion Manager® from Aries Systems Corporation

Manuscript
Click here to access/download;Manuscript;Manuscript with authors information.docx

Original Articles

Digital Insights: Exploring Women's Reproductive Intentions and Influencing Factors in the Online Sphere

Jing Xiang[1]，Xuan Sun[2▲]

[1] Jing Xiang，The first Writer, Gynecology Department of Nankai University Hospital,Nankai University,Tianjin,China

Address: 94 Nankai District, Tianjin, China

E-mail：JennifreX_DOC@163.com

TEL:0086-18522174166

ORCID ID:0009-0009-8189-1928

[2] Xuan Sun,The second writer and corresponding author,Zhou Enlai School of Government, Nankai University,Tianjin, China

Address: 94 Nankai District, Tianjin, China

Email: sunxuan@nankai.edu.cn

Tel: 0086-18649113210

ORCID:0000-0002-7450-6210

Abstract

Objectives:This study aims to provide profound insights into contemporary women's reproductive intentions and identify the factors influencing their fertility decisions by leveraging data from high-traffic websites.

Methods: Three prominent online platforms, namely TikTok, Xiaohongshu, and Kuaishou, were selected as sources. Data spanning from May 2021 to April 2024 were systematically collected using keyword search and content analysis methods. Rigorous data cleaning and classification procedures were employed to ensure the validity and accuracy of the data.

Results: High-frequency keyword analysis and binary regression techniques uncovered several pivotal factors influencing fertility intentions among online users. Significant socioeconomic determinants encompassed urban mobility (16.8%), housing pressures (25.1%), and the cost of raising children (13.1%). Maternal stress (2.6%) and concerns over workplace pregnancy discrimination (13.0%) also emerged as crucial influencers. Anxiety related to childbirth (4.0%), such as issues with compassionate care during delivery and pregnancy-related body anxieties, significantly impacted fertility decisions. Cultural beliefs (5.0%), including preferences like DINK and lesbian (2.3%), further influenced reproductive intentions. Binary logistic regression analysis highlighted that individuals with a bachelor's degree ($B = -0.54$, 95% CI [0.552, 5.34], $p < 0.05$) and those with higher education levels ($B = -1.282$, 95% CI [0.295, 1.932], $p < 0.05$) exhibited lower fertility intentions. Data analysis from 2022 ($B = -2.654$, 95% CI [0.026, 0.189], $p < 0.001$) and 2023 ($B = -3.923$, 95% CI [4.437, 6.005], $p < 0.001$) indicated significant deviations from 2021. Furthermore, residents of first-tier cities showed notable variations ($B = -3.498$, 95% CI [0.001, 0.659], $p < 0.05$). Socioeconomic factors, social support, and fertility-related anxiety/fear exhibited a significant negative correlation with fertility intention.

Conclusion: This study identifies socioeconomic conditions and support structures as pivotal determinants influencing fertility intentions within distinct social groups. Additionally, it underscores the significance of cultural beliefs and anxieties related to fertility. These findings are crucial for policymakers aiming to foster healthy attitudes toward marriage and fertility, thereby supporting societal development positively.

Keywords: Fertility Intentions, Online Platforms, Reproductive Female, TikTok, Xiaohongshu, Kuaishou

1. Background

In recent years, the global issue of declining fertility rates has gained increasing prominence[1],[2]. Despite China's shift from its strict one-child policy to policies permitting two or even three children since 2015, the anticipated rebound in fertility rates has not occurred as expected.With the rapid socioeconomic development globally, the issue of women’s fertility intentions has gradually become a focal point of concern. The urgency and importance of this issue extend beyond individual family choices, encompassing national population strategies and macro-level social structure adjustments. Women face unprecedented challenges and complex considerations in deciding whether to have children, when to have children, and how many children to have. These decisions have profound implications for family structures and the labor market, and are directly related to national sustainable development and population stability.

In China, women’s roles and challenges in family life, the workplace, and personal development are continuously evolving. According to data from the National Bureau of Statistics, China’s Total Fertility Rate (TFR) was only 1.3 in 2020 [National Bureau of Statistics. Seventh National Population Census Data[3]. By 2023, the total number of births was 9.02 million, a decrease of 540,000 from the previous year, marking the seventh consecutive year of decline; the TFR dropped to 1.02, less than half of the replacement level (2.1), making it the second lowest among major

global economies [4].Historical and international experience suggests that a TFR around 1.5 is a "highly sensitive warning line," indicating an era of low fertility that necessitates in-depth research and analysis of the underlying complex socioeconomic factors.

The rapid development of the internet has made online platforms significant channels for information dissemination in modern society, profoundly affecting people's daily lives and thought exchanges. According to reports from the China Internet Network Information Center (CNNIC), by the end of 2023, China's internet user population reached 1.092 billion [5].Additionally, data from QuestMobile in 2024 indicated that women accounted for 49.5% of internet users[6].To obtain more accurate and real-time data on fertility intentions and influencing factors, this study selected the three most popular social media platforms among women—TikTok, Kuaishou, and Xiaohongshu[6]—as survey mediums. These platforms, with their large user bases, frequent interactions, and extensive content, can reflect the attitudes and intentions of women from different regions, social roles, and socioeconomic levels toward fertility issues.

China's mobile internet data confirms [7], that as of September 2023, the combined active user base of TikTok, Kuaishou, Xiaohongshu, Bilibili, and Weibo reached 1.088 billion, with a penetration rate of 88.9%. Among these platforms, Douyin leads the first tier with 743 million monthly active users and a year-on-year growth rate of 5.1%. In the second tier, Weibo and Kuaishou have 485 million and 457 million monthly active users, respectively, while Bilibili and Xiaohongshu occupy the third tier with 210 million and 199 million monthly active users, respectively. These figures highlight the significant potential of these platforms for information collection and public opinion surveys.

This study aims to explore contemporary Chinese women's fertility intentions and the driving factors behind these intentions by analyzing data from Douyin, Xiaohongshu, and Kuaishou. Focusing on key factors such as urbanization, education level, occupational stress, and social support,through detailed data analysis, this research seeks not only to uncover the dilemmas and challenges women face in their

fertility choices but also to provide valuable empirical evidence for policymakers. The goal is to optimize the existing social support system, improve women's living and working environments, and ultimately promote fertility intentions among women of childbearing age. These research findings will offer significant references for future policy adjustments, helping to create a more inclusive and supportive social environment that fosters positive population development.

2. Methodology

2.1 Dada processing

In this study, three representative online platforms—TikTok, Xiaohongshu, and Kuaishou—were deliberately selected to systematically collect comprehensive data on women's fertility intentions spanning from May 2021 to April 2024. The choice of this timeframe was informed by China's recent adoption of the three-child policy on May 31, 2021. A diverse set of keywords related to "fertility intentions," "urbanization", "economic burden of childbearing/raising," "maternal stress," "social support for fertility," "intensive mothering," "work-life balance," "gender discrimination in the workplace," "female career development," and "cultural beliefs ,"etc,were employed.

Publicly accessible posts, comments, and video content discussing fertility-related topics by female users within the specified timeframe were extracted. A mixed-method approach combining quantitative and qualitative techniques was adopted for analysis. IBM Watson Natural Language Understanding (NLU) was utilized for text analysis, encompassing word frequency and sentiment analysis. Word frequency analysis pinpointed prevalent themes and topics, while sentiment analysis provided insights into the emotional tones expressed by women discussing fertility.

Residence city information was derived from IP addresses associated with each data entry, and details such as educational level, marital status, and age bracket were

extracted from the content of valid data submissions. Entries lacking relevant information were excluded from the dataset.

The collected data underwent rigorous purification and categorization processes to eliminate redundant and duplicated material, ensuring data integrity and precision. As this study employed a retrospective approach and relied solely on publicly available and anonymized data from online forums, it did not raise privacy concerns and therefore did not require ethical clearance.

2.2 Statistics

The dataset underwent rigorous statistical examination using IBM SPSS Statistics 25.0 software, encompassing an in-depth analysis of fertility intentions and their variations based on socio-geographic factors (age, education level,marital status, residence). Descriptive statistics were applied to summarize qualitative variables, and chi-square tests were used to initially evaluate variable associations, with statistical significance set at $p < 0.05$ (two-tailed). To ensure model validity, collinearity checks were performed, screening out variables with high collinearity or statistical insignificance. Based on this meticulous process, key predictor variables were identified and integrated into a binary logistic regression model to assess their impact on fertility intentions.

3. Results

3.1 Social-demographic Characteristics

This study compiled data from 8,162 online platform users, generating 13,500 valid data entries. Table 1 outlines the correlation between various socio-demographic factors and fertility intentions.

A comprehensive analysis uncovered notable impacts of several variables, specifically marital status, age, place of residence, and educational attainment. Among

unmarried platform users, fertility intentions were notably lower than those of married users, as evidenced by the statistically significant marital status test ($\chi^2 = 20.92$, $P < 0.001$). This finding suggests a profound connection between evolving perspectives on marriage and fertility aspirations, influenced by the financial and stability considerations associated with modern marriage, thereby affecting the fertility plans of unmarried individuals. Furthermore, while married users expressed stronger fertility desires, a notable proportion still indicated negative intentions, reflecting the potential role of marital satisfaction and financial pressures in shaping their fertility choices. This implies that although marital status is a key determinant of fertility intentions, personal decisions are also influenced by a range of internal and external factors.

In terms of age, younger users expressed significantly lower fertility aspirations, whereas those over 30 demonstrated slightly elevated intentions, albeit with a substantial proportion indicating negative leanings ($\chi^2 = 28.524$, $P < 0.001$). This suggests that younger generations may be postponing or even forfeiting fertility plans due to elevated living expenses and career demands. To foster stronger fertility intentions among this demographic, it is imperative to reduce their economic burdens and enhance the overall fertility landscape.

Significant disparities were observed based on the city of residence ($\chi^2 = 115.493$, $P < 0.001$). Bigger urban dwellers exhibited lower fertility aspirations, likely attributed to elevated living costs and resource limitations, whereas residents of smaller cities demonstrated comparatively stronger intentions. Educational achievement also played a pivotal role in shaping fertility aspirations ($\chi^2 = 42.417$, $P < 0.001$), with highly educated users expressing reduced fertility plans. This trend could be attributed to a heightened awareness of living standards and parenting expenses among this group, emphasizing the necessity for supportive policies to alleviate the childcare burden on this segment of the population.

Temporal shifts indicated the lowest fertility aspirations in 2021 and 2023, followed by a resurgence in 2024 ($\chi^2 = 129.99$, $P < 0.001$). This rebound potentially correlates with policy revisions and economic recovery in the post-pandemic era,

highlighting the importance of implementing targeted assistance measures during critical periods.

3.2 High-Frequency Terms of Fertility Intentions

Through an exhaustive analysis and categorization of frequently occurring terms extracted from online comments, discussions, and video content related to fertility intentions, we have identified several pivotal factors that exert a profound influence on individuals' fertility decisions. These factors are thoroughly outlined in Table 2, capturing the complexity and specificity of the underlying issues.

The statistical significance of socioeconomic status in influencing fertility intentions is apparent, with a chi-square value of 1.608 and a p-value less than 0.05, as illustrated in Figure 1. Among these, economic pressures, particularly urban mobility constraints and housing affordability issues, stand out as critical barriers. The term "urban mobility" specifically highlights how the challenges associated with adapting to a new city, such as job security and social integration, often lead to delays or negative impacts on fertility intentions. Our data reveals that among users who mentioned "urban mobility," 16.82% expressed negative fertility intentions, whereas only 0.03% held positive views. Similarly, "housing stress" was frequently cited, primarily due to the psychological burden of instability and lack of control over one's living space, including concerns about eviction and inability to customize one's home. This, coupled with the financial burden of raising children and potential happiness decline among urban newcomers, significantly contributes to negative fertility attitudes, emphasizing the intricate relationship between economic hardships and fertility choices.

Social support factors, on the other hand, exhibit an exceptionally high level of statistical significance ($\chi^2 = 20.961$, $P < 0.001$). Pressures related to maternal roles and workplace discrimination against pregnant women or new mothers are particularly salient, with 13.01% of users mentioning workplace discrimination against women. This discrimination, which often involves bias against those with

childbearing plans, creates a hostile work environment that discourages fertility. Notably, among those who mentioned this term, the overwhelming majority expressed negative fertility intentions, highlighting the detrimental effect of workplace discrimination on women's willingness to have children. Furthermore, the scarcity of affordable and accessible childcare options for dual-income households and the pursuit of female career advancement emerged as significant concerns, with 6.43% and 6.02% of users, respectively, reporting negative fertility intentions. These observations underscore the importance of supportive social structures and inclusive work environments in fostering positive fertility intentions.

Anxiety and fear related to fertility issues also demonstrate notable statistical importance ($\chi^2 = 6.665$, $P < 0.05$). Physical anxiety associated with pregnancy, particularly concerns about body changes such as weight gain, breast sagging, skin aging, pelvic floor muscle relaxation, and postpartum syndromes, is a significant factor mentioned by 3.34% of users. Additionally, the lack of compassionate medical care during pregnancy and childbirth, including instances of rough or inhumane treatment by healthcare professionals, is a crucial concern cited by 0.36% of respondents. This absence of empathy and support during a vulnerable time significantly impacts fertility desires, with users expressing both positive and negative fertility intentions citing this as a concern. The emotional and psychological well-being of individuals during pregnancy, therefore, plays a crucial role in shaping their fertility intentions.

While cultural perceptions did not reach statistical significance in directly influencing fertility intentions ($\chi^2 = 3.5$, $P = 0.321$), emerging cultural trends like the DINK (Dual Income, No Kids) lifestyle and evolving views on relationships were mentioned by users. The terms "DINK family" and "Deinstitutionalization of marriage," representing these cultural shifts, were frequently cited, accounting for 2.27% and 0.71% of the total user base, respectively. The DINK concept, in particular, highlights a preference for pets over children as a source of emotional fulfillment, while "demarriage" reflects changing attitudes towards traditional marriage norms and the acceptance of diverse sexual orientations. These cultural shifts, while not

statistically significant, provide insights into evolving societal values that can subtly influence individual fertility decisions.

In summary, this analysis reveals that socioeconomic factors, social support, fertility-related anxiety, and cultural perceptions, though to varying degrees of statistical significance, all contribute to shaping individuals' fertility intentions on online platforms. By preserving the specific nuances and details of these factors, we offer a comprehensive and nuanced understanding of the complex interplay between economic, social, psychological, and cultural aspects in fertility decision-making.

3.3 Regression Model of Fertility Influences

Table 3 meticulously demonstrates that the independent variables—including age range, marital status, residential city, educational level, reasons for Negative Fertility Intentions, and year of data collection—possess acceptable tolerance values and Variance Inflation Factors (VIF), thereby confirming their independence and stability in influencing fertility intentions, unhampered by multicollinearity. Consequently, these variables were incorporated into the regression model to ensure the robustness and reliability of the results.

After verifying the model's assumptions through the Hom-Lai-Shepard test (χ^2 = 4.654, ~~df = 8,~~ p = .794), further assessment of the model's performance was undertaken. The relatively modest Nagelkerke R-Square value (0.340) reflects the inherent complexity of explaining fertility intentions within the realm of social population studies, influenced by factors such as the number and explanatory power of independent variables. While acknowledging the moderate model fit, these metrics underscore the intricacies involved in capturing the full spectrum of factors that shape fertility intentions within social contexts.

Table 4 presents a comprehensive binary logistic regression model, offering insights into the influence of various independent variables on fertility intentions. Notably, educational attainment emerges as a significant predictor, with individuals possessing bachelor's (B = -0.54, $p < 0.05$, Exp(B) = 1.716, 95% CI [0.552, 5.34]) and

postgraduate (B = -1.282, $p < 0.05$, Exp(B) = 0.754, 95% CI [0.295, 1.932]) degrees exhibiting notably lower fertility intentions compared to those with lower educational levels. This translates to a decrease in fertility intentions by approximately 0.6 and 1.3 times, respectively, highlighting the profound impact of educational attainment on fertility decisions.

Temporal variations are also evident, with the year of data collection significantly influencing fertility intentions,as illustrated in Figure 2. Compared to the baseline year (2024), data from 2022 (B = -2.654, $p < 0.001$, Exp(B) = 0.07, 95% CI [0.026, 0.189]) and 2023 (B = -3.923, $p < 0.001$, Exp(B) = 5.557, 95% CI [4.437, 6.005]) reveal significant declines in fertility intentions, possibly influenced by socio-economic conditions or policy shifts during those years.

Residential city is another pivotal factor influencing fertility intentions. Residents of first-tier cities (Beijing, Shanghai, Guangzhou, Shenzhen) exhibit significantly low fertility intentions (B = -3.498, $p < 0.05$, Exp(B) = 0.03, 95% CI [0.001, 0.659]), as shown in Figure 3, likely due to high living costs and demanding work environments. In contrast, residents of Hunan province display higher fertility intentions (B = 2.834, $p < 0.05$, Exp(B) = 17.011, 95% CI [2.004, 144.4]), indicating the influence of unique socio-economic or cultural factors.

Meantime,the reasons for negative fertility intentions are statistically significant (B=40.912, df=3, $P<0.001$), indicating that these factors have a substantial impact on shaping fertility intentions. When cultural beliefs as baseline,socioeconomic factors show a significant negative correlation in the model (B=-3.843, $P<0.05$, Exp(B) = 0.43, 95% CI [0.123, 1.507]), indicating that an increase in socioeconomic factors significantly decreases fertility intentions.

Secondly, social support factors also display a significant negative correlation (B=-5.002, $P<0.001$, Exp(B) = 0.007, 95% CI [0.001, 1.041]). This suggests that a lack of social support significantly increases negative fertility intentions, emphasizing the importance of social support in shaping fertility decisions.

Finally, birth-related anxiety or fear shows a significant negative correlation (B=-2.485, $P<0.001$, Exp(B) = 0.083, 95% CI [0.023, 0.308]). This indicates that

higher levels of birth-related anxiety or fear significantly lower fertility intentions, highlighting the emotional aspects that influence fertility decisions.

Surprisingly, age and marital status do not significantly impact fertility intentions in this model, suggesting that while these two factors may not directly determine fertility intentions, they do not rule out indirect effects mediated by economic or social support factors.

In conclusion, this refined and polished model underscores the multifaceted nature of factors influencing fertility intentions, emphasizing the crucial roles of educational attainment, temporal variations, residential city, and, to a lesser extent, marital status. These findings contribute to a deeper understanding of the intricate interplay between socio-economic factors and individual characteristics in shaping fertility decisions.

4. Discussion

This study, leveraging rigorous statistical analysis and a binary logistic regression model, meticulously dissects the multifaceted and intricate factors that shape fertility intentions in Chinese society. It transcends simplistic categorizations by delving into both individual-level attributes, including education, age, and marital status, city of residence ,as well as societal dimensions that encompass economics, culture, and policy frameworks.

Firstly, the inverse association between educational attainment and fertility intentions is a multifaceted phenomenon, transcending simplistic causal interpretations. Rather, it stems from a complex interplay of diverse factors. Highly educated individuals, who prioritize personal achievement and career advancement, are often confronted with elevated living costs and intense competition, leading to delayed first-marriage ages and subsequently, postponed childbearing plans, and in some instances, fostering negative fertility intentions, as evidenced by studies conducted in Korea[8]and China[9],[10]. This pressure extends beyond the material

realm, permeating into the spiritual aspirations and expectations of these individuals. Their heightened emphasis on self-actualization and quality of life prompts a more rational and comprehensive evaluation of fertility decisions. As such, when devising fertility support policies, governments must adopt a holistic approach that transcends mere economic incentives. It is imperative to address the professional development and spiritual needs of highly educated individuals by offering tailored and comprehensive support measures that cater to their unique circumstances, thereby fostering a more conducive environment for fertility intentions.

Secondly, the fertility dilemma in the context of urbanization emerges as an inevitable byproduct of modern societal progression. The rapid urbanization process in both large and small cities, accompanied by significant costs in terms of time, economics, human resources, and fleeting opportunity costs, keeps individuals in a constant state of tension. This tension, combined with the relentless pace of life and intense competitive pressures within urban environments, frequently acts as a deterrent for young couples contemplating parenthood, as evidenced by several studies [11],[12],[13],[14].This is particularly acute for residents of first-tier cities, who grapple with acute shortages of reliable childcare resources and escalating housing prices, as highlighted in a study by Lin et al.[15]The suppressive influence of socioeconomic pressures on fertility intentions constitutes a intricate and profound societal challenge. The escalating living costs, notably high housing prices and education expenses, have significantly hindered families from realizing their fertility aspirations, as evidenced by research conducted by Chen et al.[16]and Yang et al.[17]. This is also the reason why there is often a significant difference in the willingness to have children between urban and suburban populations[18].This pressure not only shapes the fertility decisions of childbearing-age couples but also exerts a profound impact on the population structure and developmental trajectory of society at large, underscoring the urgency for comprehensive strategies to address this multifaceted issue.

Moreover, this research emphatically highlights the critical role of social support and mental health in shaping fertility intentions. Those lacking sufficient social

support and experiencing fertility-related anxiety[19],[20] are more likely to harbor negative fertility intentions. When women contemplate childbirth, whether imminent or in the long term, they often confront a labor market that is unaccommodating, characterized by invisible workplace discrimination[21],[22] that significantly hampers their ability to plan for parenthood[23]. Additionally, for women who have given birth and returned to the workforce, the arduous task of regaining prior resource allocations and career advancement prospects is fraught with challenges, as these opportunities may have been usurped or limited.

Simultaneously, the burden of motherhood emerges as a pivotal determinant influencing women's reproductive decisions and overall well-being. This pressure is exacerbated by the persistence of traditional gender role expectations within families, coupled with the multifaceted demands placed on women in contemporary society. Within the traditional Chinese family culture, gender roles are distinctly defined, with men traditionally occupying roles as primary providers and decision-makers, while women are primarily responsible for domestic duties and childcare. This entrenched gender division has reinforced stereotypes, resulting in women shouldering disproportionately heavier familial responsibilities compared to men.Despite remarkable strides made by women in socioeconomic advancement as society evolves, the lingering influence of traditional family values continues to impact their lives, shaping their experiences and choices surrounding fertility and parenthood. The phenomenon of "intensive mothering" arises amidst this backdrop. Contemporary mothers strive for professional success while simultaneously embracing their roles as wives and mothers, confronting the dual pressures of work and family. They must navigate the demands of the workforce to achieve economic independence, all while managing the arduous tasks of housework and childcare upon returning home. This excessive burden of motherhood consumes women's time, energy, and often, their mental and physical health, leading to a marked decline in well-being[24].

As a result, the pressure of intensive mothering significantly dampens married women's desire for a second or third child. Fear of an imbalanced work-family life and concerns over inadequate nurturing and companionship for their children deter

many from expanding their families. These apprehensions are reinforced through social media, friends, and family interactions, inadvertently influencing unmarried women's views on marriage and parenthood.

To alleviate this profound dilemma, the government must significantly increase investment in urban childcare resources, optimize resource allocation, and encourage enterprises and social organizations to participate in the construction of childcare service systems, thereby fostering a diversified childcare support network, enhancing the quality of public kindergartens and childcare centers, and reducing childcare costs. Additionally, a series of targeted economic incentive policies should be implemented, including more proactive fiscal and taxation policies, fertility allowances, housing incentives, and education subsidies. Strengthening social security systems and enhancing basic public services for couples of childbearing age, promoting economic development model transformation and industrial structure upgrading to generate more employment opportunities and income streams, and regulating the real estate market are also crucial steps to ultimately alleviate the economic pressures faced by these couples and create more favorable conditions for their fertility plans.

Regarding family culture, a concerted and multifaceted approach involving all sectors of society is necessary to mitigate the economic and emotional pressures associated with childbirth and parenting. This includes strengthening public service systems, expanding parental leave benefits, and refining fertility support measures. Simultaneously, society must actively promote gender equality, dismantle outdated gender role stereotypes, and foster a culture where men are encouraged to share domestic and childcare responsibilities equally with women. Furthermore, the media and public figures hold significant influence in shaping societal attitudes towards fertility and family values. They must leverage their platforms to advocate for positive fertility narratives and promote informed discourse that acknowledges the complexities and joys of parenthood.

Collaboration among governments, society, and communities is vital in fostering a fertility-friendly environment. This entails reinforcing mental health services tailored to couples of childbearing age, advocating for family-friendly workplaces that

support female employees' fertility rights and career aspirations, and mobilizing social organizations to contribute to a diverse support network for families. By enhancing mental health support and societal perceptions of parenthood, these efforts aim to bolster fertility intentions.

The primary limitation of this study stems from its exclusive reliance on social media data, which may not fully encapsulate the diverse perspectives and nuanced experiences of women across different demographics. To mitigate this limitation and gain a more comprehensive view, future research should endeavor to broaden its scope by integrating data from a wider array of social media platforms and adopting a multimodal approach that includes qualitative methods such as interviews and focus groups, alongside longitudinal studies. This multifaceted approach will facilitate a deeper understanding of the intricate interplay of factors that shape women's reproductive intentions and their evolution over time, taking into account shifts in socioeconomic conditions, cultural norms, and policy frameworks.

In conclusion, this study presents a valuable contribution to the understanding of women's reproductive intentions in the digital era, shedding light on the complex factors that influence these decisions. By acknowledging and addressing the study's limitations and advocating for more comprehensive research methodologies, we can pave the way for policymakers, healthcare providers, and advocates to collaboratively create supportive environments that empower women to make informed choices about their reproductive health, ultimately fostering more equitable and sustainable societies.

5. Conclusion

The findings underscore the need for holistic policy interventions that transcend simplistic economic incentives and address the professional development, mental health, and social support needs of diverse populations. As women continue to navigate the dual pressures of career advancement and motherhood, fostering a

supportive environment that acknowledges the complexities and joys of parenthood becomes imperative. Ultimately, this study serves as a call to action for policymakers, healthcare providers, and society at large to collaborate in creating a more inclusive and supportive landscape for reproductive autonomy and family well-being.

Conflict of interest

The authors declare no competing financial or personal interests that could have appeared to influence the work reported in this paper.

Declaration

The authors consent to publication.

Author contributions

The first author is primarily responsible for research methodology design, data analysis, and manuscript writing, while the second author is responsible for data organization, manuscript revision, and providing funding support.

Funding

Youth Project of the Humanities and Social Sciences Fund of the Ministry of Education, Research on the Social Coordinated Mechanism of Medical-Patient Community Construction (19YJC840030).

Reference

[1] United Nations Department of Economic and Social Affairs, Population Division. World population prospects 2022: Summary of results. New York: United Nations Department of Economic and Social Affairs, Population Division; 2022.

[2] Hiroki Nakatani, Aging and shrinking population: The looming demographic challenges of super-aged and super-low fertility society starting from Asia, Global Health & Medicine, 2023, Volume 5, Issue 5, Pages 257-263, Released on J-STAGE November 06, 2023, Advance online publication October 20, 2023, Online ISSN 2434-9194, Print ISSN 2434-9186, https://doi.org/10.35772/ghm.2023.

[3] National Bureau of Statistics. Seventh National Population Census Data [EB/OL]. (2021-05-11) [2024-05-25]. https://www.stats.gov.cn/sj/pcsj/rkpc/7rp/indexch.htm.

[4] China Economic Net. National Population Changes Sampling Survey Data [EB/OL]. (2024-01-18) [2024-05-25]. http://www.ce.cn/xwzx/gnsz/gdxw/202401/18/t20240118_38870849.shtml.

[5] China Internet Network Information Center. 53rd Statistical Report on China's Internet Development [EB/OL]. (2024-03-22) [2024-05-20]. http://www.cnnic.cn//n4/2024/0322/c88-10964.html.

[6] QuestMobile GROWTH User Profile Label Database. QuestMobile 2024 "She Economy" Insights Report: Nearly 610 Million Active Female Users, with Monthly Average Times of 13.9 Hours in Shopping a

nd 13.2 Hours in Reading [EB/OL]. (2024-03-06) [2024-05-20]. https://www.questmobile.com.cn//research/report/1764860900811051009.

[7] QuestMobile TRUTH China Mobile Internet Database. 2023 New Media Ecology Insights: Industry User Scale Reaching 1.088 Billion, User Flow and Diversion Entering New Stage; Platforms Seeking Growth and Monetization via Two Major Paths - Industry Research Report [EB/OL]. (2023-09) [2024-05-26]. http://www.questmobile.com.cn/research/report/1726888249161519105.

[8] Park S , Hwang M .Factors on Childbirth Intention of Working Married Women in Korea[J].Journal of Public Society, 2022.DOI:10.21286/jps.2022.11.12.4.48.

[9] Baizan P, Nie W. The Impact of Education on Fertility During the Chinese Reform Era (1980-2018): Changes Across Birth Cohorts and Interaction with Fertility Policies. Eur J Popul. 2024 Jan 30;40(1):7. doi: 10.1007/s10680-023-09691-2. PMID: 38289489; PMCID: PMC10828303.

[10] Yan Z, Hui L, Wenbin J, Liuxue L, Yuemei L, Bohan L, Lili W. Third birth intention of the childbearing-age population in mainland China and sociodemographic differences: a cross-sectional survey. BMC Public Health. 2021 Dec 14;21(1):2280. doi: 10.1186/s12889-021-12338-8.

[11] Tan J , Kang S . Urban Housing Prices and Migration′ s Fertility Intentions: Based on the 2018 China Migrants′ Dynamic Survey[J]. Papers, 2021. DOI: 10.48550/arXiv.2112.07273.

[12] Meng L, Peng L, Zhou Y. Do housing booms reduce fertility intentions? Evidence from the new two-child policy in China[J]. Regional Science and Urban Economics, 2023, 101: 103920. ISSN 0166-0462. https://doi.org/10.1016/j.regsciurbeco.2023.103920.

[13] Ge B. Investigation on Residents′ Fertility Intention of a County in Dali State in Yunnan Province Under Universal Two-Child Policy[J]. Medicine and Society, 2019.

[14] Menon M, Perali F. Cost Of Raising Children, Child Poverty And Fertility Decisions[J]. Rivista Internazionale di Scienze Sociali, 2019, 156. DOI: 10.26350/000518_000032.

[15] Lin T, Mu L, Liu Y. A study on the impact factors of procreating willingness of urban and rural families under the background of ″comprehensive two-child policy″ : a case study of urban and rural families around Chaohu Lake. Northwest Popul J. 2020;41:78–87.

[16] Chen, SM., Zhang, Y. & Wang, YB. Individual differences in relative fertility costs and fertility benefits and their effects on fertility desire for a second child in China: a latent profile analysis. Reprod Health 16, 110 (2019). https://doi.org/10.1186/s12978-019-0770-1.

[17] Yang, H., Han, R. & Wang, Z. Third-child fertility intention and its socioeconomic factors among women aged 20–34 years in China. BMC Public Health 23, 821 (2023). https://doi.org/10.1186/s12889-023-15719-3

[18] Yang H, Han R, Wang Z. Third-child fertility intention and its socioeconomic factors among women aged 20-34 years in China[J]. BMC Public Health, 2023, 23(1): 821. DOI: 10.1186/s12889-023-15719-3.

[19] He Y, Abdul Wahab NET, Muhamad H. Factors impacting fertility anxiety among Chinese young women with marital status differences[J]. Heliyon, 2023, 10(1): e23715. DOI: 10.1016/j.heliyon.2023.e23715.

[20] Zeng T, Li B, Zhang K, et al. The association between childbirth-related fear, childbirth readiness, and fertility intentions, and childbirth readiness as the mediator[J]. Reprod Health, 2023, 20: 62. DOI: 10.1186/s12978-023-01607-x.

[21] Kim JH, Oh SS, Bae SW, Park EC, Jang SI. Gender Discrimination in the Workplace: Effects on Pregnancy Planning and Childbirth among South Korean Women[J]. Int J Environ Res Public Health, 2019,

16(15): 2672. DOI: 10.3390/ijerph16152672.
[22] Zhang C, Wei L, Zhu Y, Teng L, Zhang W, Xu J, Qin M, Jiang N, Alias H, Wong LP. Fertility intentions among young people in the era of China′ s three-child policy: a national survey of university students[J]. BMC Pregnancy Childbirth, 2022, 22(1): 637. DOI: 10.1186/s12884-022-04873-y.
[23] Zhou Y. The dual demands: gender equity and fertility intentions after the one-child policy. J Contemp China. 2019;28:367–84.
[24] Elliott, S., Powell, R., & Brenton, J. (2015). Being a Good Mom: Low-Income, Black Single Mothers Negotiate Intensive Mothering. Journal of Family Issues, 36(3),351-370. https://doi.org/10.1177/0192513X13490279.

Table1.Overview Of Social Geographical Characteristics And Fertility Views Of Network Users

**Social-Demographic Factors**	**Category**	**Fertility Intention （N，100%）**		**N，100%▲**	**χ^2**	**P**
		**Positive**	**Negative**			
Marrital Status	Single	27，0.33%	6275，76.88%	6302，77.21%	20.92	<0.001
	Married	26，0.32%	1834，22.47%	1860，22.79%		
Age Interval	<20	3，0.04%	57，0.70%	60，0.74%	28.524	<0.001
	20-30	25，0.31%	2288，28.03%	2313，28.34%		
	>30	25，0.31%	5764，70.62%	5789，70.93%		
Resident City	Top Tier Cities	16，0.20%	1719，21.06%	1735，21.26%	115.493	<0.001
	Hubei	1，0.01%	2238，27.42%	2239，27.43%		
	Hunan	25，0.31%	3787，46.40%	3812，46.71%		
	Shandong	3，0.04%	12，0.15%	15，0.19%		
	Tianjin	8，0.10%	353，4.32%	361，4.42%		
Education	Bachelor	9，0.11%	4164，51.02%	4173，51.13%	42.417	<0.001
	Beyond Bachelor’s	34，0.42%	3598，44.08%	3632，44.50%		
	Sub-Bachelor’s	10，0.12%	347，4.25%	357，4.37%		
Date Collection Year	2021	-	1108，13.58%	1108，13.58%	129.99	<0.001
	2022	45，0.55%	1686，20.66%	1731，21.21%		
	2023	2，0.03%	2037，24.96%	2039，24.98%		
	2024	6，0.07%	3278，40.16%	3284，40.23%		
Sum		53，0.65%	8109，99.35%	8162，100%		

Top Tier Cities：Beijing/Shanghai/Guangzhou/Shenzhen;100%▲ The denominator is the total population.

Table 2. Summary Of High Frequency Entries Of Network Users

Reason Categories	Birth claim (high-frequency entry)	Fertility Intention (N,100%)		N,100%▲	χ^2	P
		Positive	Negative			
Socioeconomic Status	Urban Mobility	3，0.04%	1373，16.82%	1376，16.86%	1.608	<0.05
	Housing Stress	6，0.07%	2045，25.06%	2051，25.1%		
	Child-Rearing Costs	5，0.06%	1066，13.06%	1071，13.12%		
	Urban Newcomers' Happiness Decline	3，0.04%	632，7.74%	635，7.78%		
Social Support	Maternal Stress	-	211，2.59%	211，2.59%	20.961	<0.001
	Potential Workplace Pregnancy Discrimination	18，0.22%	1044，12.79%	1062，13.01%		
	Childcare Shortage For Dual-Income Parents	-	525，6.43%	525，6.43%		
	Career Development And Self-Pursuit	-	491，6.02%	491，6.02%		
Birth-Related Anxiety / Fear	Lack Of Compassionate Care During Childbirth	4，0.05%	25，0.31%	29，0.36%	6.665	<0.05
	Pregnancy-Related Physical Anxiety	10，0.12%	263，3.22%	273，3.34%		
	Uncertainty About Work-Life Balance During Pregnancy	-	9，0.11%	9，0.11%		
	Psychosocial Adjustment Disorders	1，0.01%	16，0.20%	17，0.21%		
Cultural Beliefes	DINK (Dual Income No Kids)	1，0.01%	38，0.47%	39，0.48%	3.5	0.321
	Diverse Sex Orientation (Lesbian)	2，0.02%	184，2.25%	186，2.27%		
	Deinstitutionalization Of Marriage	-	58，0.71%	58，0.71%		
	Pessimistic Views On Social And Environmental Development	-	129，1.58%	129，1.58%		
Sum		53，0.65%	8109，99.35%	8162，100%		

100%▲ The denominator is the total population.

Table 3. Collinearity Diagnostics for Independent Variables

Cofficient a

Model		Unstandardized Coefficients		Standardized Coefficients	t	P	95.0% Confidence Interval		Collinearity statistics	
		B	S.E	Beta			Lower	Upper	Tolerance	VIF
1	(Constant)	-4.996	1.766		-2.83	<0.05	-8.457	-1.535		
	Age Range	0.005	0.002	0.03	2.118	<0.05	0.018	0.11	0.609	1.642
	Reasons for Negative Fertility Intentions	-0.004	0.001	-0.041	-3.68	<0.001	-0.006	-0.002	0.951	1.051
	Marital Status	-0.015	0.003	-0.077	-4.845	<0.001	-0.021	-0.009	0.479	2.088
	City of Residence	-0.007	0.001	-0.081	-5.85	<0.001	-0.009	-0.005	0.623	1.604
	Education Level	-0.005	0.002	-0.039	-3.277	<0.05	-0.009	-0.002	0.835	1.197

a Dependent Variable: Fertility Intention

Table 4. Binary Logistic Regression Model for Fertility Intention

Variables in the Equation

		B	S.E	Wald	df	P	Exp(B)	95% Confidence Interval	
								Lower	Upper
Step 1a	**Education Level**			3.585	2	<0.05			
	Bachelor	-0.54	0.579	0.87	1	<0.05	1.716	0.552	5.34
	Above Bachelor’s	-1.282	0.48	0.345	1	<0.05	0.754	0.295	1.932
	**Year of Data Collection**			50.362	3	<0.001			
	2021	14.125	1173.247	0	1	0.99	13.6	0.348	19.5
	2022	-2.654	0.504	27.673	1	<0.001	0.07	0.026	0.189
	2023	-3.923	1.241	9.988	1	<0.05	5.557	4.437	6.005
	**City of Residence**			28.716	4	<0.001			
	Top Tier Cities	-3.498	1.572	4.95	1	<0.05	0.03	0.001	0.659
	Hubei	2.566	0.706	13.219	1	<0.001	13.008	3.263	51.866
	Hunan	2.834	1.091	6.745	1	<0.05	17.011	2.004	144.4
	Shandong	0.918	0.431	4.541	1	<0.05	2.505	1.076	5.828
	**Marital Status (Single)**	-0.158	0.496	0.102	1	0.75	0.854	0.323	2.256
	**Age Range**			0.399	2	0.819			
	20-30years	-0.354	0.861	0.17	1	0.68	0.702	0.13	3.791
	>30years	-0.28	0.458	0.375	1	0.54	0.755	0.308	1.855
	**Reasons for Negative Fertility Intentions**			40.912	3	<0.001			
	Socioeconomic Status	-3.843	0.64	1.738	1	<0.05	0.43	0.123	1.507
	Social Support	-5.002	0.917	29.735	1	<0.001	0.007	0.001	1.041
	Birth-Related Anxiety / Fear	-2.485	0.667	13.896	1	<0.001	0.083	0.023	0.308
	**Constant**	6.88	1.107	38.603	1	<0.001	972.861		

a Variables entered in Step 1: Education Level, Year of Data Collection, City of Residence, Marital Status, Reasons for Negative Fertility Intentions,Age Range.

Top Tier Cities：Beijing/Shanghai/Guangzhou/Shenzhen

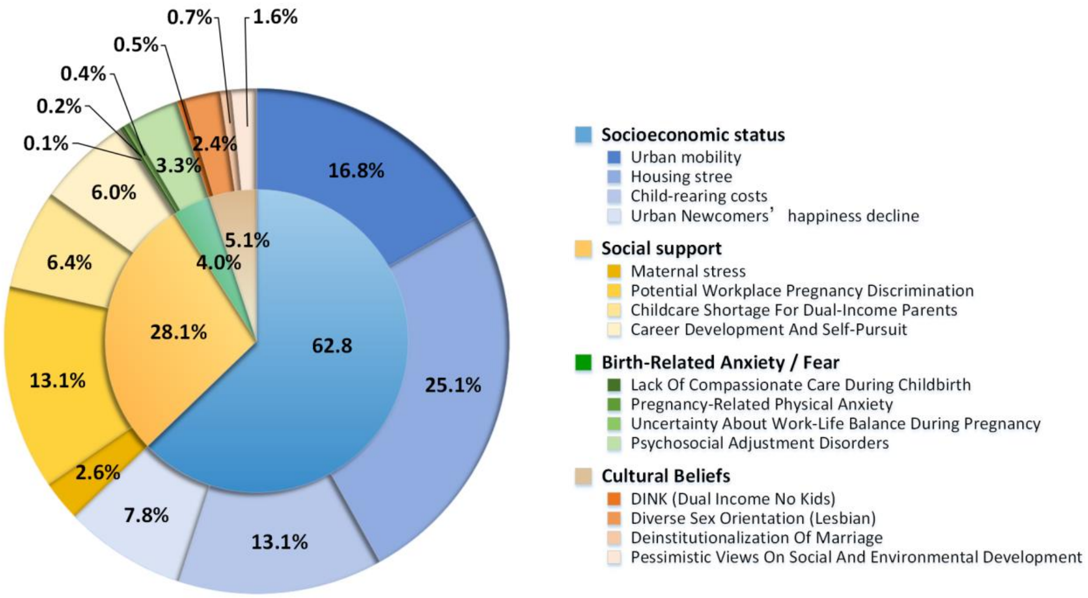

Figure 1.Classification of Reasons for Negative Fertility Intentions Among Internet Users

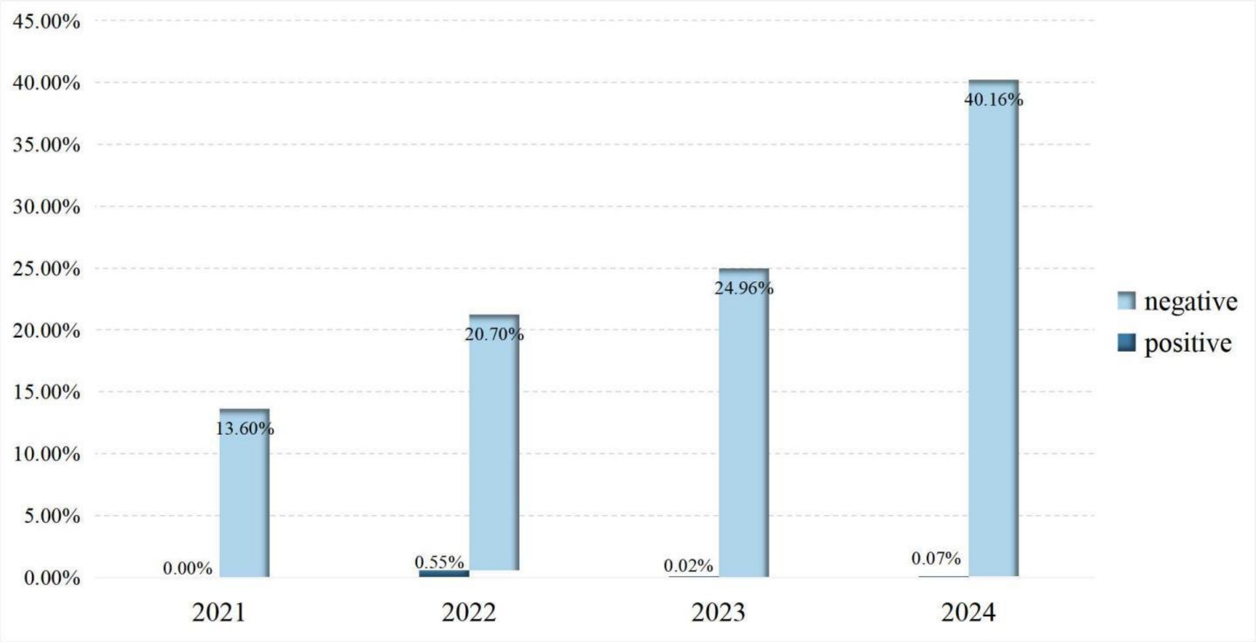

Figure 2.Temporal Changes in Fertility Intentions of Internet Users

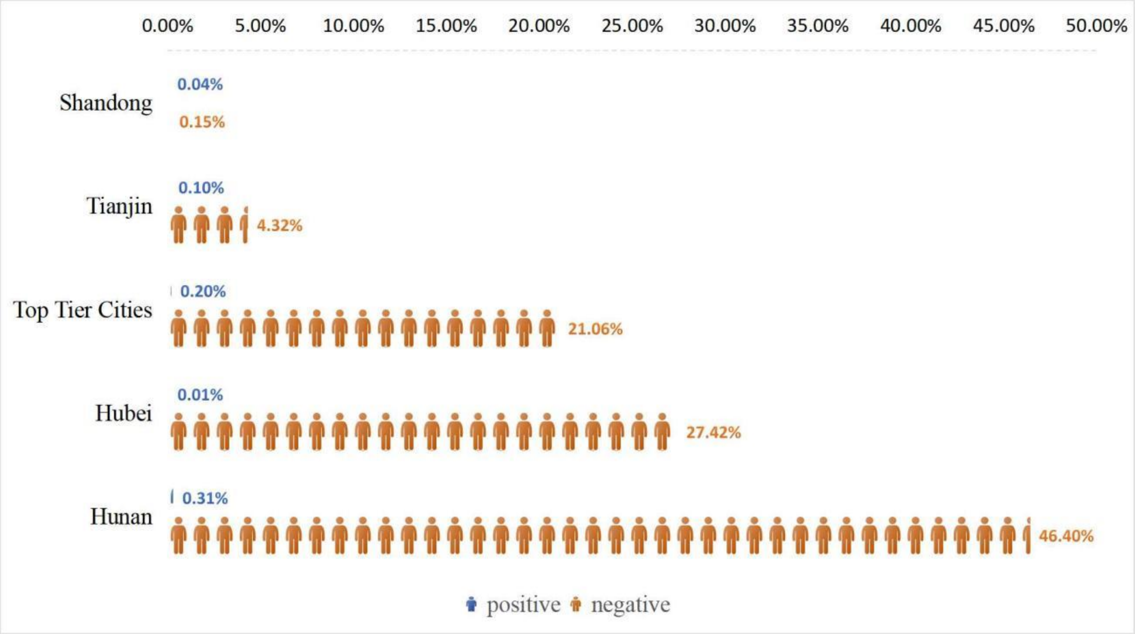

Figure 3.Overview of Fertility Intentions Between Urban Areas

 Click here to access/download;Figure;Figure 1.png

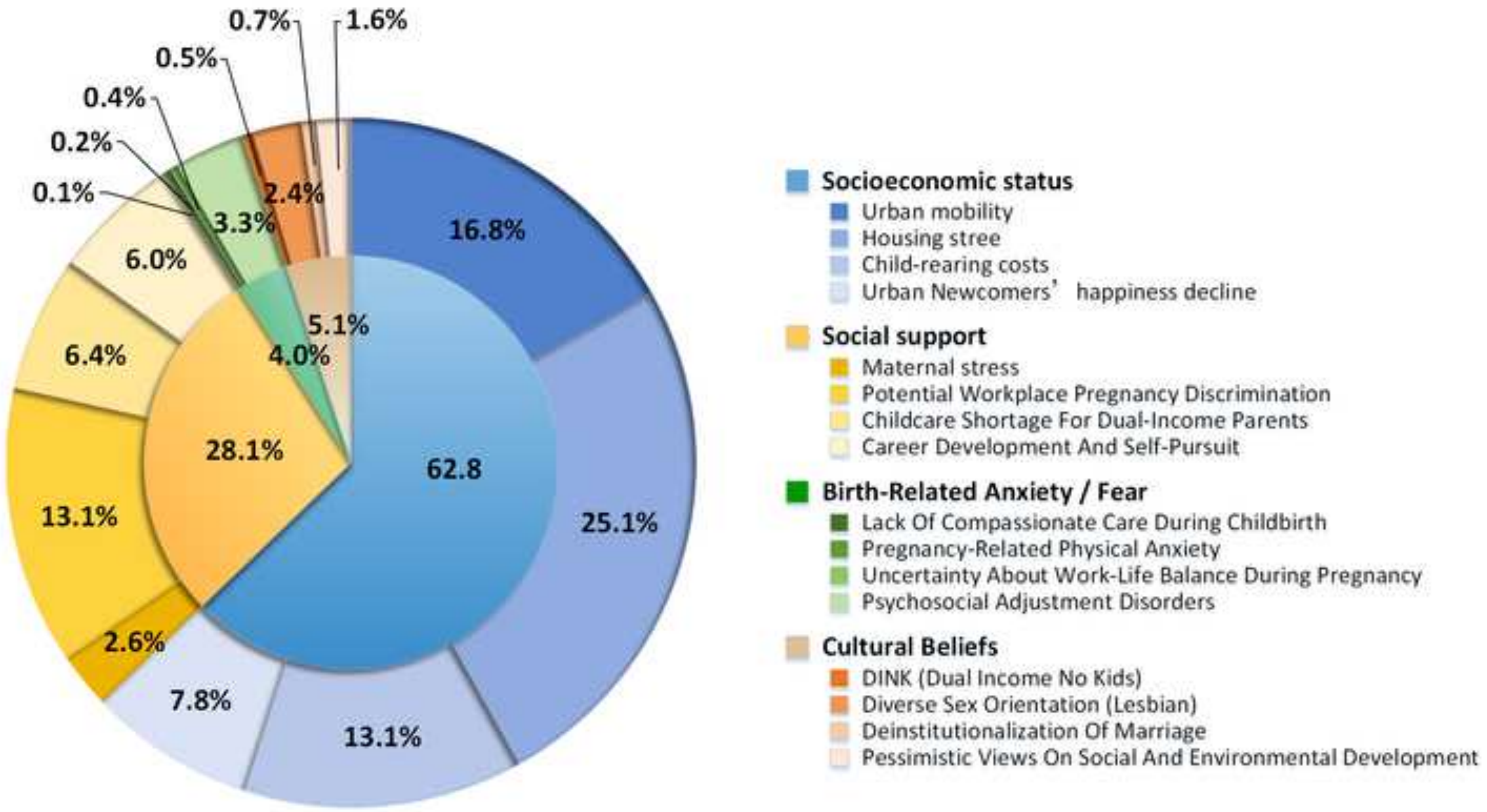

Click here to access/download;Figure;Figure 2.jpg

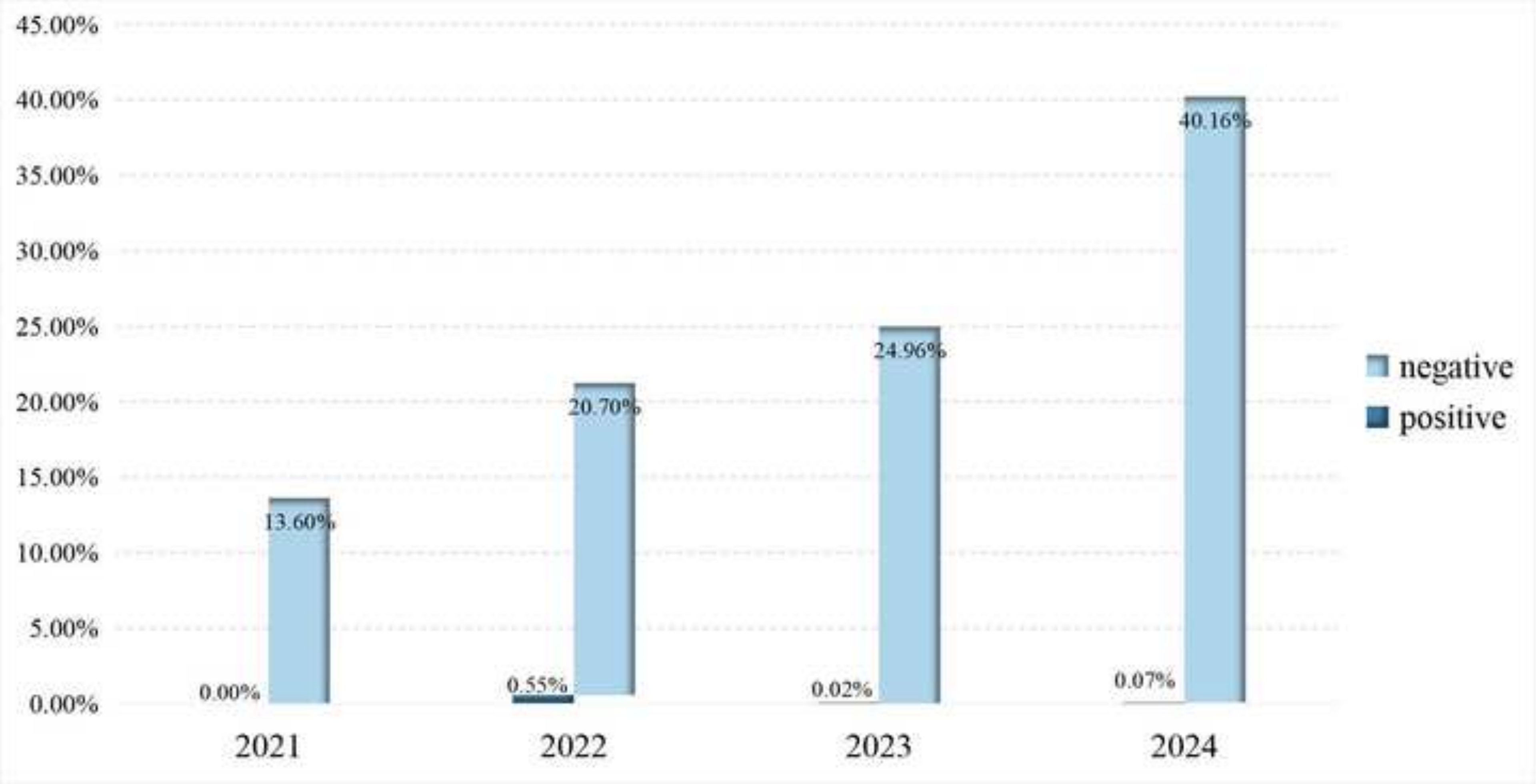

Click here to access/download;Figure;Figure 3.jpg

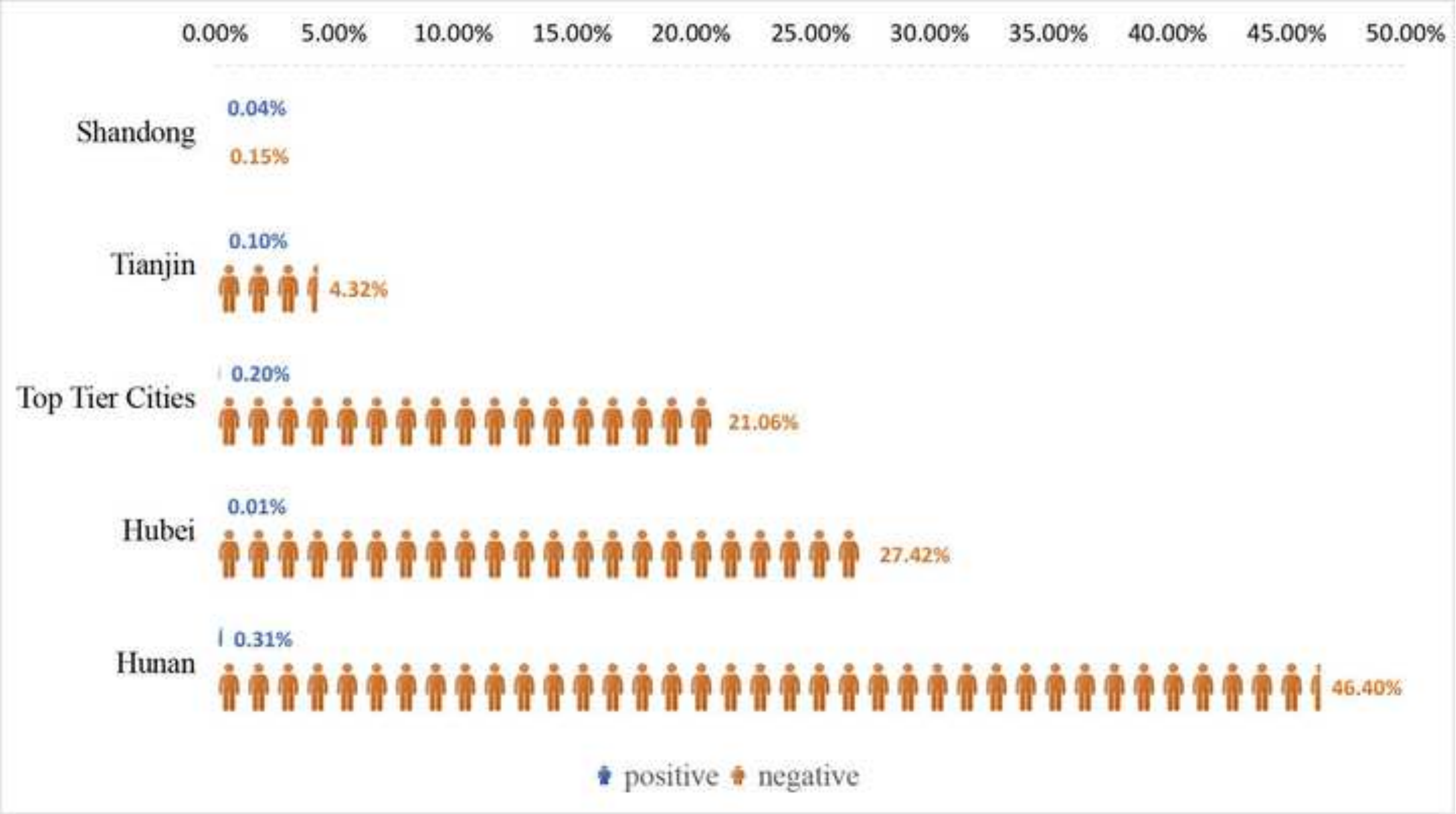

Click here to access/download
Supporting Information - Compressed/ZIP File Archive
Table.rar
