# Supplementary material for: Digital insights: Analyzing the reproductive intentions and influencing factors among urban women in China through online platforms
Source: PLoS One. 2025 Jul 28;20(7):e0327570. doi: 10.1371/journal.pone.0327570 (PMC12303354; doi:10.1371/journal.pone.0327570)
Supplement: Attachment — Submitted filename: point to point response to reviewers.docx [file pone.0327570.s007.docx]

**We would like to express our gratitude to the anonymous reviewers for their valuable suggestions, and to the editor for their diligent work. We have carefully considered each comment and made the necessary revisions to the manuscript.**

-------------------------------------------------------------------------------------------------------

 Part one: **Comments to the Author**

1. Is the manuscript technically sound, and do the data support the conclusions?

The manuscript must describe a technically sound piece of scientific research with data that supports the conclusions. Experiments must have been conducted rigorously, with appropriate controls, replication, and sample sizes. The conclusions must be drawn appropriately based on the data presented.

Reviewer #1: Yes

Reviewer #2: Yes

Reviewer #3: No

Response to Reviewer #3:

We sincerely appreciate your thoughtful feedback. We have carefully addressed your concerns and made significant improvements to the manuscript, especially in terms of methodological rigor and data clarity. Specifically, we have:

(1) Clarified Methodology: We have revised the methodology section to provide more precise details about the experimental design, controls, and sample size. This should ensure that the study is described with the necessary technical rigor.
(2) Enhanced Data Presentation: We have also provided more in-depth explanations of the data analysis process, ensuring transparency and rigor in how the conclusions were drawn from the data.

We hope these revisions better address your concerns regarding the technical soundness of the research and the adequacy of the data supporting the conclusions.

2. Has the statistical analysis been performed appropriately and rigorously?

Reviewer #1: Yes

Reviewer #2: Yes

Reviewer #3: No

Response to Reviewer #3:

 Thank you for your valuable comments. Upon review, we realized that our original dataset only included negative and positive fertility intentions, which might have overlooked the significant role of neutral intentions. Neutral attitudes represent a state of uncertainty or a temporary postponement of fertility decisions, influenced by various internal or external factors. Such attitudes are crucial for providing a comprehensive understanding of current fertility trends among women on the internet. Therefore, we have now included the previously excluded neutral fertility intention data and performed a systematic reanalysis of the entire dataset. This reanalysis has led to a complete revision of the results and interpretations, offering a more nuanced and holistic view of fertility intentions.

3. Have the authors made all data underlying the findings in their manuscript fully available?

The [PLOS Data policy](http://track.editorialmanager.com/CL0/http://www.plosone.org/static/policies.action%23sharing/1/010f019402a00fec-ce6fb51d-8b53-4c2a-bb40-0abfe9c2fa34-000000/ZUYd2K0EAUAx9GLMlGxRXbh2TA-qVly9qxcbbKtvYkE=191) requires authors to make all data underlying the findings described in their manuscript fully available without restriction, with rare exception (please refer to the Data Availability Statement in the manuscript PDF file). The data should be provided as part of the manuscript or its supporting information, or deposited to a public repository. For example, in addition to summary statistics, the data points behind means, medians and variance measures should be available. If there are restrictions on publicly sharing data—e.g. participant privacy or use of data from a third party—those must be specified.

Reviewer #1: Yes

Reviewer #2: Yes

Reviewer #3: No

Response to Reviewer #3:

We sincerely appreciate your feedback on the data availability. We fully understand and respect the journal’s data sharing policy. In response to your concern, we will ensure that the raw data underlying our findings is made publicly available by uploading it as a supplementary file.We hope this fully addresses your concerns and aligns with the journal’s requirements.

4. Is the manuscript presented in an intelligible fashion and written in standard English?

PLOS ONE does not copyedit accepted manuscripts, so the language in submitted articles must be clear, correct, and unambiguous. Any typographical or grammatical errors should be corrected at revision, so please note any specific errors here.

Reviewer #1: Yes

Reviewer #2: Yes

Reviewer #3: No

Response to Reviewer #3:

Thank you for your careful review and insightful comments regarding the clarity and language of the manuscript. We have thoroughly proofread the entire manuscript to correct any typographical or grammatical errors. We have also made revisions to ensure the manuscript adheres to the standards of academic English, with particular attention to improving clarity and readability. We believe these revisions have addressed your concerns and significantly improved the overall presentation of the manuscript.

Part two: **Review Comments to the Author**

**Reviewer #1**: 


3. Experiments, statistics, and other analyses are performed to a high technical standard and are described in sufficient detail.
Yes, however, it needs more detail. For example, the preprocess techniques and classification the authors did on need to be more detailed in methodology (See my comments)

Response to Reviewer #1:

Thank you for your valuable feedback. In response to your suggestion, we have expanded the Methodology section to provide more detailed descriptions of both the data preprocessing techniques and the sentiment classification process, as follows:

(1) Data Preprocessing: We have added a more thorough explanation of the data collection process, which involved using a custom streaming tool to capture fertility-related discussions on various platforms. We now clarify the specific keywords employed and the methodology used for gender classification. Additionally, we address steps taken to mitigate potential biases in the data and ensure its robustness for the analysis.
(2) Classification Methodology: We have provided more detail on the sentiment analysis process, specifically outlining our use of IBM Watson’s Natural Language Understanding (NLU) platform and cross-validation with the Chinese Sentiment Knowledge Base (CSKB). Furthermore, we explain how to using manual review to ensure accurate sentiment categorization.

These revisions can be found in Section 2: Methodology of the revised manuscript. We believe these additions address your concerns and enhance the clarity and transparency of our research process.

5. The article is presented in an intelligible fashion and is written in standard English.
Yes, however, some parts need to be improved (see my comments)

Labor is essential because it focuses on the key factors in the Online Sphere. As the methodologies and results are original, we accept the document on condition that our comments are considered.

**Reviewer #2**: The paper is good. Climate/climate change, and weather effects are two of the major factors that can biologically affect fertility in women. Amongst other factors affecting fertility, can the authors consider climate or climate change as one of the factors?

Response to Reviewer #2:

Thank you for your valuable comments and insightful suggestion. We appreciate your observation regarding the influence of climate and climate change on fertility.

Indeed, during our analysis of web interaction data, we observed that a significant portion of participants expressed concerns and pessimistic sentiments related to environmental issues, including climate change and pollution. These environmental factors were frequently mentioned as contributing to their anxieties about the future, and were particularly salient in the context of fertility decisions. We have since expanded the manuscript to incorporate these concerns and have added relevant discussions and citations from existing research on the impact of environmental factors, such as climate change, on fertility intentions.

We believe that the integration of these issues strengthens the overall analysis and reflects the broader social and environmental context that influences fertility decisions.

**Reviewer #3**: This paper uses data from three social media platforms in China to explore trends in fertility intentions and reasons for variation between people. There are several major things that must be addressed before this paper can be published.

1) The analysis needs to be changed. Unless I am mistaken, for the table 1 analysis the authors have calculated column percentages (i.e. distribution by the outcome variable) and then looked at whether there is a significant difference by the exposure variable. This is the wrong way round for their causal inference (i.e. they need to test whether there is a difference in intentions between the different exposure groups). As a result, the description of their results is entirely incorrect as they are actually reporting the percent within those who have negative intentions. For example, they say ‘In terms of age, younger users expressed significantly lower fertility aspirations, whereas those over 30 demonstrated slightly elevated intentions, albeit with a substantial proportion indicating negative leanings (χ² = 28.524, P < 0.001).’. This conclusion is based on the column % (of those who have negative intentions, proportionally more are under 30) whereas by age group it is the other way round – 98.9% of under 30s have negative intentions and 99.5% of those over 30 have negative intentions. Table 2 is also based on inference from column %.

Thank you for your detailed and insightful comments. We appreciate your careful attention to the statistical analysis, and we fully acknowledge the concerns regarding the causal inference in our methodology.

We have reviewed the analysis carefully and agree with your observation that the approach to calculating column percentages and the way we described the results was incorrect. Specifically, the causal inference was not properly tested, as we indeed should have focused on examining differences in fertility intentions across exposure groups (i.e., age groups, education levels, etc.), rather than within the group of individuals with negative intentions.

To address this, we have revised our statistical analysis as follows:

(1) Revised Statistical Approach: We have re-conducted the analysis using the correct approach, where we tested for differences in fertility intentions between different exposure groups (e.g., age, education level, etc.) rather than within the subgroups of negative fertility intentions. This corrected analysis allows for more accurate causal inference.
(2) Clarified Results and Descriptions: The results have been rewritten to correctly reflect the revised analysis. For example, regarding age, we now correctly describe that 98.9% of those under 30 expressed negative fertility intentions, whereas 99.5% of those over 30 did the same, and we make sure to present these findings based on the exposure group (i.e., age) rather than the outcome variable (i.e., fertility intentions).
(3) Table 1: We have revised Table 1 by recalculating the percentages based on the correct statistical inference. This ensures that the table now reflects the intended analysis and supports the revised findings.

We believe these revisions adequately address the concerns you raised, and we have updated the manuscript accordingly. Thank you again for pointing out this important issue, and we hope the revised version meets your expectations.

2) Lack of detail on data and analysis
The reader must be able to evaluate the reliability of the analysis, but the lack of detail about the data and its operationalisation makes that impossible.

- What is a content analysis? How is this done? How much of this analytical technique is reliant on human interpretation of clustering? What is a sentiment analysis? What is a high-frequency term and a birth claim? Could the authors give examples of some of the quotes used that were used to infer a positive/negative intention or the explanations given for their childbearing decision-making in table 2?

Thank you for your valuable feedback. In response to your comments, we have made the following revisions to the manuscript to clarify the methodology and improve transparency:

(1) Sentiment Analysis:We used sentiment analysis to classify fertility-related discussions into positive, negative, and neutral categories, employing Natural Language Processing (NLP) techniques. This process was largely automated with minimal reliance on human interpretation to ensure objective classification.
(2) Content Analysis and High-Frequency Term Extraction:Content analysis was performed to identify key themes and patterns in the data, using the TF-IDF algorithm for high-frequency term extraction. We also incorporated manual checks to ensure the accuracy and reliability of the results.

We have updated the Methodology section (pages 6-7) to provide clearer explanations of these techniques. We hope this addresses your concerns.

- What language were the videos in? Was it transcripts of videos that were analysed, and if so, how reliable is the transcription of the audio?
- How was the data about sociodemographic profile gathered? The only description is at the top of page 6 that these were ‘extracted from valid data submissions’. Is it publicly available on individual’s profiles? How reliable is this data? Could the authors provide a descriptive profile of their sample so that an assessment of sample representativeness can be made? They say their analysis was limited to female users – how was this established? How was this information then grouped by the authors into the categories they chose? Does this categorisation match the data distribution or was it informed by theory etc.? Why have they chosen just these variables? For example, number of children already is likely to be highly influential on fertility intentions. The description of what a ‘top-tier’ or ‘first-tier’ city comes far too late in the text.

Thank you for your thoughtful feedback. We have revised the manuscript and added additional details based on your suggestions (please refer to pages 5-8 for updates). Below are our responses:

(1) **Language and Video Transcriptions**: Our research primarily analyzes real-time interaction data from three major platforms, focused on the interactions of Chinese online women. The videos are transcribed manually into text, accommodating Mandarin and widely understandable dialects. Content that could lead to comprehension bias (e.g., difficult-to-understand speech or heavy use of metaphors) is excluded. The transcribed text is then processed and analyzed using NLP and TF-IDF for categorization and statistical analysis .
(2) **Sociodemographic Profile**: Demographic data are primarily derived from user profiles, IP locations, and self-reported information. Data points missing relevant demographic variables or difficult to obtain are excluded. We used user-generated content (posts, comments) to infer key sociodemographic factors, but note that these data were not always fully reliable. For reliability, we cross-checked self-reported data with inferred attributes, and excluded any ambiguous cases (page 6-7). We have also included a descriptive profile of our sample in the revised manuscript to allow an assessment of sample representativeness.
(3) **Gender Classification**: The study focuses on female users due to their central role in fertility-related decisions. We conducted gender classification through several steps:
1) Public user information: including usernames, profile descriptions, and profile pictures.
2) Language pattern analysis: examining pronouns and engagement behaviors.
3) Exclusion of ambiguous cases: profiles with neutral usernames or anonymous profiles were excluded to ensure data accuracy.

Given current limitations in gender classification in China, we defined “female” as biological females for the purpose of this study. Although gender identity based on appearance or language may not have been fully captured, we minimized errors through multiple validation steps (page 5).

(4) **Predictive Variables and Categorization**: Our study focuses on predictive variables such as rough categories of age and education level, and IP address. These are extracted from user-generated content and cannot accurately measure specific fertility intentions (e.g., first or subsequent childbearing). Our approach is more suited for identifying general fertility attitudes, but we acknowledge the limitations of this method in terms of precise categorization (page 7).
(5) **Definition of 'First-Tier' Cities**: We regret the late definition of 'first-tier cities' in the manuscript. It was indeed an oversight not to introduce this term earlier. We have now added a clear definition of first-tier cities at the appropriate place in the manuscript for better clarity (page 9).

- Why did the authors choose just these 3 apps? Also they mostly say they are looking at TikTok, Xiaohongshu and Kuaishou but at one point they switch TikTok to Douyin.

Thank you for your insightful comment. We initially considered several platforms, including Douyin (formerly TikTok), Kuaishou, Xiaohongshu, Tencent Weibo, and Zhihu, as they are well-known social media outlets in China with diverse user bases. However, after reviewing platform traffic data (detailed in the background section, pages 3-4), we narrowed our focus to Douyin, Kuaishou, and Xiaohongshu. These three platforms have a significant female user demographic, particularly among those aged 18-34, which is key for studying fertility attitudes.

Regarding the mention of TikTok, we had originally referred to Douyin by its international name, but we have now standardized the reference to Douyin throughout the manuscript for consistency.

We hope this explanation clarifies our platform selection.

- What exactly is the outcome variable? The authors just say they are looking at fertility intentions (which has a very specific definition in the demographic literature which I would recommend the authors engage with and decide if this is the best terminology), which they then categorise into positive and negative. What does this mean? Not wanting children vs wanting children? Was child number intentions considered? Timing of childbearing?

We would like to express our sincere gratitude to the reviewer for your professional and insightful review, as well as your valuable comments. In this study, **the outcome variable** focuses solely on generalized fertility attitudes,or desires among women, which are categorized into positive, neutral, and negative attitudes primarily using NLP technology, in combination with manual review. These fertility attitudes represent women’s overall stance on childbearing, shaped by their current internal and external environments, and should not be interpreted as indicators of specific subsequent fertility behaviors. Rather, they reflect an observation of their intentions.

While it is acknowledged that fertility attitudes may vary between the first child and subsequent children, our study aims to provide a comprehensive overview of women’s generalized fertility attitudes as observed through the lens of social media, utilizing a large dataset. We recognize the limitations inherent in this study and have addressed these limitations in the manuscript.

- The logistic regression would be easier to interpret if odds ratios were presented and reference groups clearly outlined. How are the factors that influence childbearing operationalised in the regression?

Thank you for your valuable feedback. We agree that presenting the odds ratios and clearly defining reference groups would improve the interpretation of the logistic regression results. In the revised manuscript, we have made the following updates:

(1) **Odds Ratios and Reference Groups:**
    The revised manuscript now clearly defines the reference categories for each predictor variable. For example, neutral fertility intentions were used as the reference group in the multinomial logistic regression model. For categorical variables such as marital status and education level, the reference groups are single and bachelor’s degree, respectively. The odds ratios (OR) for each factor are now presented in Table 4, with 95% confidence intervals and p-values to provide clearer interpretation.
(2) **Operationalization of Factors Influencing Childbearing:**
    The factors influencing fertility intentions were operationalized as follows:
1) Age: Divided into age groups (below 20, 20-30, above 30).
2) Marital Status: Coded as binary (married vs. unmarried).
3) Geographic Location: Based on residence in top-tier cities (e.g., Beijing, Shanghai, Guangzhou, Shenzhen) versus other regions.
4) Education Level: Grouped by education attainment (e.g., below bachelor's, bachelor's, above bachelor’s).
5) External Factors: Such as socio-economic status, social psychological factors, socio-cultural Factors and social support were also incorporated as categorical predictors.

These operationalizations are further explained in the Methodology section of the revised manuscript, ensuring that the regression model's predictors are clearly defined and their relationship with fertility intentions is transparent.

3) Writing style needs to be adjusted to make points based on evidence
Overall this paper tends to make statements about the reasoning for their analysis and trends in fertility in China, but do not support these with suitable evidence like citations or results from their analysis. This is particularly problematic in the discussion which needs to be reviewed.

The authors could also be much more critical of this analysis and what it can and can’t tell the reader/policy-makers. They list one point in the discussion that the analysis is limited to users of these apps, but they could go much further. They could discuss some of the things outlined in point 2 above about the data quality, but also much more about how their sample is likely to be selective. I would imagine, for example, that people who make videos about having children are more likely to complain than talk about the virtues. Is there evidence to that effect?

Thank you for your valuable feedback and suggestions. We fully agree that the writing style needs to be adjusted to ensure that all points are supported by appropriate evidence. To address your concerns, we have made the following improvements:

(1) Enhancing Evidence Support: We have revised the paper to include additional citations and results from our analysis to substantiate our claims about the reasoning for our analysis and trends in fertility in China. Every assertion in the discussion section will now be backed by empirical data, statistical results, or relevant literature, ensuring that our arguments are not only presented as statements but are grounded in solid evidence. For instance, trends observed in fertility intentions will be cross-referenced with existing studies, and our own data will be cited to demonstrate the alignment or deviation from broader societal trends.
(2) Critical Evaluation of the Analysis: We have expanded our critical discussion to address what our analysis can and cannot tell the reader, particularly for policy-makers. While we previously mentioned that the analysis is limited to users of certain apps, we now provide a more thorough critique. We discuss the data quality, including its reliance on self-reported user-generated content, and the potential biases inherent in this approach. We also highlight the selective nature of our sample—specifically, users who engage in content creation related to fertility might not represent the broader population. As you pointed out, users discussing fertility may be more likely to voice dissatisfaction or concerns, thus contributing to a skewed representation of attitudes. We have included a section discussing this potential bias and, where possible, cited relevant research to support these observations. For example, we reference studies that suggest that social media discussions on sensitive topics like childbearing often tend to focus on challenges and frustrations rather than positive experiences.
(3) Further Considerations for Future Research: Building on these discussions, future research should adopt a more nuanced approach. This could include embodied ethnography to explore how women navigate medical trauma and workplace discrimination, as well as experimental vignettes to test policy receptivity across urban tiers. Additionally, cross-national comparisons are needed to better understand the unique interactions between state policies, market forces, and family dynamics in China.

4) Stance that policymakers need to boost fertility is problematic and should be adjusted

The authors repeatedly present low fertility as a 'problem'. I would recommend the authors use a more objective description of fertility trends, for example removing references to low fertility as an ‘issue’ or a ‘concern’. My fear is that by adding opinions to descriptions of low fertility implies it is an inherently negative outcome when this is not necessarily true (i.e. it is the consequences of ageing populations that can be negative if suitable adaptations aren't in place).

Further, I would urge caution with the presentation of policy implications. The authors frequently refer to the need to raise and boost fertility with state intervention. I would be in favour of a different presentation that moves away from the authors seeming to promote raising fertility back to replacement levels, especially given that 'replacement level' is a somewhat arbitrary modelling construct that takes no account of population productivity which is also important for addressing the consequences of ageing populations. A more neutral policy implication would be that individuals are unable to have the children that they want due to structural constraints, like all the reasons for low fertility the authors identify in their analysis. The authors touch on this at the bottom of page 4 into page 5. I would suggest a clearer focus on this line of policy making to avoid any issues of suggesting state intervention policies targeted at raising fertility back to a 'desirable' levels.

Thank you for your insightful feedback. We understand your concerns and appreciate your perspective. Based on your suggestions, we have revised our discussion and policy implications accordingly.

(1) Reframing Fertility Trends: We have adjusted our language to adopt a more objective stance in describing fertility trends. Instead of framing low fertility as an inherent ‘problem,’ we now refer to it as a demographic shift that warrants attention. We emphasize that the consequences of low fertility are more closely tied to population ageing and the potential socio-economic impacts if appropriate adaptations are not made. By doing so, we align with your suggestion to avoid implying that low fertility itself is necessarily negative, but rather focus on the broader structural factors that contribute to demographic changes.
(2) Policy Implications and Structural Constraints: In response to your comments about state intervention, we have reframed our policy recommendations. Rather than advocating for policies that explicitly aim to "raise fertility to replacement levels," we now focus on the structural constraints individuals face in achieving their desired fertility outcomes. Our discussion emphasizes that factors such as economic pressures, work-life balance, and social support systems limit reproductive choices. By highlighting these barriers, we aim to propose policies that address the root causes of fertility constraints, ensuring that individuals have the autonomy and resources to make informed reproductive choices.

This revised approach aligns with your suggestion to present a more neutral policy stance that acknowledges the complexities surrounding fertility trends and the need for supportive interventions without assuming an explicit goal of increasing fertility levels.

5) Article needs to be thoroughly proofread as there are many typos (e.g. dada processing) and grammatical errors. Greater specificity is needed throughout as well, for example:

a. The title and abstract need to be clear that this is a paper focused on China
b. Abstract is hard to be understood without more explanation. The actual effect sizes can be removed, and instead add greater detail to what is meant. For example, what is DINK? What does ‘significant impact’ mean? What does ‘significant deviations’ mean? What does ‘notable variations’ mean?

Thank you for your detailed comments. We completely agree with your observations and have made the following revisions:

(1) Clarifying the Title and Abstract:We have revised the title to explicitly indicate that the paper is focused on China. This should make the geographical scope of the research clearer to the reader.

The abstract has been rewritten for greater clarity. We have removed the specific effect sizes and instead provided clearer explanations of the findings. In particular, terms like DINK (Dual Income, No Kids) are now defined, and we have avoided vague terms like "significant impact" and "notable variations." Instead, we describe the specific trends and factors influencing fertility intentions in a more understandable manner.

(2) Proofreading and Grammar:We have thoroughly proofread the entire manuscript and corrected all spelling mistakes and grammatical errors.

We believe these revisions address your concerns and enhance the overall clarity and precision of the manuscript. Thank you again for your valuable input.
