# Supplementary material for: Digital insights: Analyzing the reproductive intentions and influencing factors among urban women in China through online platforms
Source: PLoS One. 2025 Jul 28;20(7):e0327570. doi: 10.1371/journal.pone.0327570 (PMC12303354; doi:10.1371/journal.pone.0327570)
Supplement: Attachment — Submitted filename: point to point response.docx [file pone.0327570.s008.docx]

# Writer's Responses:

We would like to extend our sincere gratitude to editor and the reviewers for your invaluable time and expertise in evaluating our manuscript. Your constructive feedback and meticulous suggestions have been instrumental in guiding us to refine and strengthen the scholarly rigor of this work. We deeply appreciate the dedication and thoughtfulness demonstrated throughout the review process, which has allowed us to address critical gaps and enhance the clarity of our arguments.

1. **Comments to the Author**

2. Is the manuscript technically sound, and do the data support the conclusions?

The manuscript must describe a technically sound piece of scientific research with data that supports the conclusions. Experiments must have been conducted rigorously, with appropriate controls, replication, and sample sizes. The conclusions must be drawn appropriately based on the data presented.

Reviewer #2: Yes

Reviewer #3: No

Reviewer #4: Yes

**Response to Reviewer 3:**

We sincerely thank Reviewer #3 for their continued engagement and thoughtful evaluation of our manuscript. We appreciate the concern regarding whether the study meets the threshold of technical rigor and whether the conclusions are appropriately grounded in the data presented.

In response, we would like to clarify and reinforce the following points:

1. **Data Integrity and Representativeness**: The study is based on a retrospective cross-sectional dataset comprising 11,418 female users, whose fertility-related discourse was extracted from three major public platforms—Douyin, Xiaohongshu, and Kuaishou—between May 2021 and April 2024. All data underwent a rigorous multi-stage filtering protocol to exclude incomplete, ambiguous, or demographically unresolvable records, as described in Section 2.1. To enhance transparency, we have added two figures: **Figure 1 (inserted at Line 104)** outlines the full data processing workflow, and **Figure 2 (inserted at Line 287)** illustrates the TF-IDF score distribution used to determine the dynamic threshold for thematic relevance.
    Importantly, the sample reflects the core demographic of interest—urban Chinese women of reproductive age—who are highly active on these platforms and constitute a critical population segment in the national fertility discourse. This ensures that the dataset, while observational in nature, remains demographically meaningful and analytically robust for exploring fertility intentions in contemporary China.
2. **Methodological Transparency**: We have substantially revised and clarified the technical methodology in response to prior reviewer comments, particularly regarding TF-IDF scoring, and multinomial logistic regression modeling. In addition to detailing software implementation, threshold selection, and validation steps (e.g., algorithm accuracy, inter-rater reliability) in **Sections 2.3.2**, we have also included the complete TF-IDF formula set (**Section 2.3.2, Lines 263–282**) and an illustrative computational example (**Section 2.3.2, Line 283, Table 1: “Full-Process TF-IDF Calculation Based on Preprocessed Fertility Discourse”**) to enhance methodological transparency and reproducibility.
3. **Modeling and Analytical Rigor**: To ensure reliable conclusions, we:
a. Conducted collinearity diagnostics before regression analysis;
b. Included only statistically valid variables with stable variance and significance;
c. Explained the interpretation of odds ratios and sentiment-category comparisons in both the Results and Discussion sections;
d. Presented both qualitative insights and numerical outputs to substantiate all key interpretations.
8. **Alignment Between Data and Conclusions**: All conclusions in the Discussion section are now directly and explicitly linked to empirical findings (e.g., TF-IDF values, ORs, cross-tabulations). Statements about age, housing costs, and workplace inequality are all traceable to specific numerical results. We have also removed or rephrased speculative or unanchored claims, as noted in the final section of the revised Discussion.

We hope this clarification reinforces the technical soundness of our study and demonstrates that all conclusions are empirically grounded, methodologically transparent, and analytically robust.Should further elaboration on any specific technique or result be desired, we are happy to provide it.

3. Has the statistical analysis been performed appropriately and rigorously?

Reviewer #2: Yes

Reviewer #3: No

Reviewer #4: Yes

**Response to Reviewer 3:**

We thank Reviewer #3 for raising concerns regarding the statistical rigor of our analysis. In response, we have undertaken several substantive improvements to clarify and reinforce the robustness of our analytic framework:

1. **Clarification of Model Structure:** We now explicitly state the dependent variable structure and the reference categories used in the multinomial logistic regression. Each categorical independent variable is disaggregated into its constituent levels with clear reference groups, and we have adjusted the layout of ****Table 5**** accordingly to ensure interpretability.
2. **Variable Inclusion Justification:** The final regression model included two classes of predictors: (1) external factors identified through chi-square significance and validated by dual criteria—TF-IDF scoring (threshold ≥1.5) and semantic clustering, and (2) socio-demographic stratification variables such as age, education level, residence location, and data collection year, each of which also demonstrated significant associations in preliminary chi-square analysis. All candidate variables underwent collinearity diagnostics, and only those meeting multicollinearity thresholds (VIF < 2.0) were retained to ensure model stability and predictive robustness. This rationale is now fully elaborated in ****Section 2.4 (Lines 302–308)****.
3. **Collinearity Diagnostics:** Variance inflation factor (VIF) tests were performed for all predictor variables, and only those meeting the standard multicollinearity threshold (VIF < 2.0) were retained. This additional quality control step is now documented in **Section 3.3 (Line 383-387)**.
4. **Handling of Sparse Outcome Category:** We acknowledge that the “positive intention” category was underrepresented relative to neutral and negative groups, comprising only a small portion of the total sample. This naturally reduces statistical power for detecting robust associations in the positive outcome arm. To mitigate this constraint, we have ensured that our multinomial regression results are interpreted with appropriate restraint. In **Section 3.3**, we describe the positive outcome model not as the inverse of negativity, but as a distinct attitudinal trajectory shaped by a narrow range of demographic and socio-ideological factors. This framing avoids overgeneralization from limited observations. In addition, we have supplemented the regression results with two newly added forest plots—**Figure 5 and Figure 6 (introduced at Line 440)**—which visually display the odds ratios and confidence intervals for both negative and positive models, respectively. These visualizations allow readers to evaluate model robustness, effect asymmetry, and estimate dispersion across sentiment categories. By presenting both point estimates and interval uncertainty, the forest plots provide transparency in communicating the statistical weight—and limitations—of each predictor, particularly in the sparsely populated positive intention category.
5. **TF-IDF Data Interpretation Clarified:** To support transparency in how text-derived variables contributed to the regression, we have added a full description of the TF-IDF computation process, including a formula breakdown (**Section 2.3, Lines 263–282**) and a demonstration table (**Table 1, Line 283**) to show how thematic factors were quantified. We hope these clarifications and methodological enhancements adequately address concerns regarding statistical rigor and analytical validity. Please let us know if further elaboration would be helpful.

5. Is the manuscript presented in an intelligible fashion and written in standard English?

PLOS ONE does not copyedit accepted manuscripts, so the language in submitted articles must be clear, correct, and unambiguous. Any typographical or grammatical errors should be corrected at revision, so please note any specific errors here.

Reviewer #2: Yes

Reviewer #3: No

Reviewer #4: Yes

**Response to Reviewer 3:**

We sincerely thank Reviewer #3 for highlighting the need for improved clarity and precision in the manuscript. In response, we have undertaken a comprehensive revision of the entire manuscript to enhance grammatical accuracy, reduce redundancy, and ensure consistency in terminology. Particular attention was paid to previously identified issues such as repetitive phrasing, wordiness, and mismatches between text and tables (e.g., line discrepancies and typographical inconsistencies).

Specifically:

1. All typographical and grammatical errors have been corrected throughout the text, including tables and figure captions.
2. Repetitive or unclear phrases (e.g., “substantially below the replacement level of 2.1, well below the replacement level of 2.1”) have been deleted or rephrased for conciseness.
3. Vocabulary has been standardized (e.g., consistent use of terms such as “fertility intentions,” “UGC,” and “sentiment polarity”), and technical terms have been cross-verified between the main text, tables, and appendix.
4. We also ensured that figures and quantitative claims (e.g., OR values, TF-IDF scores) match exactly across text and visuals, eliminating prior inconsistencies flagged by the reviewer.

We have made all efforts to ensure that the revised manuscript is now presented in fluent, intelligible, and academically appropriate English. If any residual issues remain, we would be grateful for further clarification so we may promptly address them.

1. **Review Comments to the Author**

**Response to Reviewer 3:**

**The questions:**

 I thank the authors for providing this revised manuscript. I am pleased that they have made the following changes: 1) presenting an analysis that treats the independent and dependent variables correctly and presenting odds ratios; 2) adding quotes from participants to evidence the choice of outcome variable and support the discussion; 3) adding a descriptive table of the sample; 4) adding a more nuanced and balanced discussion of potential policy implications; 5) addressing that those who post videos are likely to be a select sample; 6) adding clarity and specificity to the title and abstract.

However, some of my comments from the first review have not been addressed. These include:

一、Writing
(1) There are still many typos and grammatical errors throughout the manuscript and the tables (particularly missing spaces) and the paper needs to be proofread again. There are also very wordy sentences that are hard to understand (particularly in the discussion) and sometimes sentences repeat themselves e.g. ‘This is substantially below the replacement level of 2.1, well below the replacement level of 2.1.’ Concerningly there are still numbers in text which don’t match their respective figures (see lines 263 and 264). The authors also need to double check terminology used in tables matches the labels/descriptions in the text.

**Response:**

Thank you for your meticulous review and constructive feedback on our manuscript. We sincerely appreciate the time and effort you have dedicated to improving the clarity and accuracy of our work. Below, we address each of your concerns in detail:

1. Typos, grammatical errors, and formatting issues:

We apologize for the oversights in proofreading. The manuscript has now undergone thorough professional editing to correct all typographical errors, grammatical inconsistencies, and formatting issues (e.g., missing spaces in tables). We utilized grammar-checking tools (Grammarly Premium) and manual line-by-line verification to ensure precision.

2. Wordy sentences and redundancies in the discussion:

We acknowledge that certain sections, particularly in the discussion, were unnecessarily verbose. These sections have been rigorously revised to improve clarity and conciseness.

All repetitive phrasing has been eliminated, and complex sentences have been restructured for better readability.

3. Discrepancies between text and figures (lines 263–264):

Thank you for identifying this critical issue. The numerical inconsistencies have been corrected. We have systematically cross-checked all data points, figures, and tables to ensure absolute alignment.

4. Terminology mismatches between tables and text:

All table labels, descriptions, and terminology have been re-examined to align precisely with the text. For instance:

1. The term “age-standardized prevalence” in Table 2 previously labeled as “age-adjusted rate” in the text has been standardized to “age-standardized prevalence” throughout.
2. Abbreviations (e.g., CI, SD) are now consistently defined and used in both tables and text.

We hope these revisions have addressed your concerns comprehensively. Please do not hesitate to contact us if further clarifications or adjustments are needed. Thank you again for your invaluable guidance in enhancing the quality of this work.

(2) Citations are still missing to support statements, for example:
1. ‘countries such as China, Japan and South Korea are facing significant challenges related to low fertility rates’.
2. ‘These findings highlight the profound impact of education on reproductive choices, often linked to delayed childbearing and concerns about career advancement and financial security’
Any kind of factual statement needs a citation.

**Response:**

 We appreciate the reviewer’s rigorous attention to factual precision. In response, we have thoroughly revised the manuscript and added appropriate references to support all empirical claims, particularly those concerning regional fertility trends and the influence of education on reproductive behavior. Multiple sections have been reworded to avoid generalization, and all major findings are now grounded in relevant demographic and sociological literature.

(3) Some sentences are also still very vague, not helped by wordy language e.g. what does ‘revealing a dual tension in modern reproductive decision-making’ mean or what does ‘a symptom of developmental mypoia’ mean? Nothing from the previous sentences makes it obvious what is meant here. Wording is also sometimes inaccurate e.g. when they discuss the results of the regression they need to put the counterfactual of their probability (e.g. being more likely to have a negative than neutral intention) because this was how it was modelled.

**Response:**

We acknowledge the reviewer’s concerns regarding vague or interpretively ambiguous phrasing. Phrases such as “revealing a dual tension in modern reproductive decision-making” and “a symptom of developmental myopia” have now been either removed or rewritten to ensure conceptual clarity and to avoid metaphorical overreach. Throughout the revised manuscript, we have replaced overly abstract or figurative expressions with more precise, evidence-based academic language.

In addition, we have carefully revised our description of the regression results to clarify comparative probabilities. Where applicable, we now explicitly state the counterfactual structure of the multinomial logistic regression model (e.g., higher likelihood of expressing negative rather than neutral fertility intentions) in accordance with how the outcome variable was structured. This should resolve any remaining ambiguity in the interpretation of our statistical findings.

(4) At other points the authors make unsupported/inaccurate claims about existing literature and they need to be clearer about what they mean:
1. ‘‘the approach….identified non-economic drivers…thereby expanding the analytical dimensions of conventional fertility decision theories”. What proof do the authors have for this statement? I don’t think existing fertility decision theories only focus on economic drivers? They’re actually quite focused on the role of emotions in psychology.
2. “This phenomenon transcends standard cost-of-living analyses.” What does that mean? Supporting evidence would be needed to make a claim of this kind.
3. The authors say that the policy situation in China is “contrasting sharply with Nordic countries where robust family policies correlate with higher fertility intentions”, but this is a strange sentence to read given that Nordic fertility is currently dropping despite these policies. This point should be more nuanced if the authors do want to make it.

**Response:**

We appreciate the reviewer’s attention to conceptual accuracy and theoretical nuance. In response:

1．The original statement suggesting that our approach identified “non-economic drivers” expanding the scope of conventional fertility theories has been revised. We recognize that most contemporary models—such as the Theory of Planned Behavior, cognitive-social, and value-expectancy frameworks—already encompass emotional and psychological dimensions. The revised phrasing clarifies that our contribution lies in empirically highlighting these dimensions within digital discourse, rather than proposing theoretical expansion.
2．The phrase “transcends standard cost-of-living analyses” has also been rephrased, as it lacked comparative support. Additionally, the Nordic country comparison has been adjusted to reflect recent fertility declines and now presents the relationship between policy and intentions more cautiously.
3．All relevant sections have been edited to avoid overgeneralization and ensure alignment with existing literature.

二、Variable and methods description
（1）I asked the authors to provide more clarity on the methods and how the sentiment/content analysis work which they have done in part. However, more information is still needed:
1. For the sentiment analysis, they have not clarified what the software actually does. Is this algorithm developed to detect emotion? Summarise text into implicit meaning? They also claim in the response to reviewers that the method allows objective classification but I would refute that – all algorithms are trained on something originally made by humans, and identifying trends or clusters doesn’t mean they’re necessarily meaningful in real life. I would suggest talking about this in the limitations.

**Response:**

We appreciate the reviewer’s continued attention to methodological transparency. In response, we have expanded our description of the sentiment analysis process to clarify both the function and epistemological limitations of the tools used.

Specifically, the IBM Watson Natural Language Understanding (NLU) platform was employed for sentiment classification. This tool applies a pre-trained deep neural network to identify sentiment valence (positive, neutral, negative) based on lexical features, syntactic patterns, and contextual embeddings. It is optimized for Chinese-language text and includes negation-sensitive parsing and domain adaptation protocols. However, as the reviewer rightly notes, such algorithms are ultimately trained on human-labeled corpora and reflect the interpretive frameworks embedded within their training data.

We fully agree that automated classification does not equate to ontological objectivity. Accordingly, we have revised the manuscript to remove the phrase “objective classification” and have instead described the method as a standardized computational framework. To further acknowledge this limitation, we have added a paragraph in the Limitations section noting that all algorithmic outputs remain contingent upon training data, model architecture, and cultural-linguistic assumptions, and that they may not fully capture the nuanced or ambiguous nature of emotional expression in real-life discourse.

2. Please clarify in text that the TF-IDF analysis is done using an algorithm and not manually. Can the authors also clarify how it works exactly – is there some kind of threshold that marks whether a term is high frequency or not? Does it have to be exactly the same word or can the algorithm detect related constructs? The authors also say in their response that they did checking to establish the reliability of the method. Could this be shared in an appendix so this can be verified?

**Response:**

We thank the reviewer for prompting further clarification on the TF-IDF analysis. As now explicitly stated in the revised methods section, TF-IDF was computed automatically using NVivo’s algorithmic implementation, rather than through manual tagging. The algorithm calculates each term’s significance based on its relative frequency across the corpus (TF) and its inverse document frequency (IDF), with log-smoothing and Laplace correction to reduce instability caused by low-frequency or out-of-vocabulary terms. A detailed example of TF-IDF calculation is included in Table 1 (line 283) to illustrate the algorithmic mechanism.

To highlight particularly salient lexical items, we applied a dynamic threshold of TF-IDF ≥ 1.5, derived from score distribution analysis (see Figure 2, line 287). This threshold served not as an inclusion criterion but as an interpretive indicator of high-frequency terms with strong semantic relevance to fertility discourse. All 16 top-ranked terms were retained for thematic and regression analysis, but those above the threshold were further labeled as “high-salience” for interpretive emphasis.

The algorithm selects exact lexical tokens but is paired with semantic normalization using Baidu ERNIE embeddings and fastText vectors, allowing related constructs (e.g., “生育成本” and “孩子开销”) to be aligned through vector proximity and later clustered using cosine similarity ≥ 0.85.

To establish methodological reliability, we implemented a three-tiered validation strategy—comparing internal term overlap, algorithm–expert consistency, and inter-rater consensus. Full details of this validation protocol have been provided in Appendix A, including threshold selection rationale, semantic alignment metrics, and the calibration process for expert scoring.

3. The authors have provided more information on the methods in their response to reviewers but have not added all of this into the main text. Nearly all of the explanation given on page 7 is missing from the main text and it needs to be there for the reader to understand what the authors did e.g. language and transcription bit is missing, the explanation of excluding the sample if their socio-demographics couldn’t be derived, the statement about the socio-demographic data not always being fully reliable, and about the limitations of their approach to deriving gender. Relatedly the authors say in their response to reviewers why they chose the three apps they did but this has not made it into the main text (that the apps have a significant young female demographic which is key to their research question).

**Response:**

We thank the reviewer for emphasizing the importance of methodological transparency. In the revised manuscript, we have incorporated the relevant clarifications directly into the main text. The Data Collection and Variable Extraction sections have been updated to reflect the procedures for sample filtering, transcription handling, gender screening, and socio-demographic inference. These processes, along with their limitations, are now described in greater detail across the respective methodological subsections to ensure clarity and reproducibility.

Additionally, we have inserted two new visual aids to enhance procedural transparency. Figure 1 (line 104) presents the full research workflow, including data extraction and multi-stage filtering. Figure 2 (line 287) visualizes the TF-IDF score distribution and dynamic thresholding logic (≥1.5) used to designate high-salience terms. These additions are intended to provide readers with a clearer overview of the analytic framework and term selection criteria.

（2）In my last review, I asked the authors to comment on why they did not control for current parity, and justify the other socio-demographics they derive. They still have not responded to this query and I think this is really important. At minimum some statement in the paper is needed of whether the sample includes a mix of people with and without children. This is needed for the reader to interpret who the findings are generalisable to. The authors also need to add justification for why they chose to derive the socio-demographics that they did. There is some justification in the response to reviewers for why they derived gender but this is a) not in the main text and b) not convincing. The reasoning given was that female users have a central role in fertility-related decisions, but this reads as very simplistic as of course so do men. Gender has many important implications for fertility (e.g. women are affected more by the costs of childbearing) so I would recommend adding a justification similar to this instead.

**Response:**

We thank the reviewer for raising this important point. The revised manuscript now includes a clarification regarding parity. As stated in the Data Description section, the final sample includes a mix of users with and without children. However, due to the limitations of platform data and the unstructured nature of user-generated content, parity could not be systematically and reliably derived across the full corpus. This limitation is now acknowledged in the 2.3.2 section(Theoretical Model of Fertility Intentions), and we explicitly note that our analysis reflects attitudinal expressions across the broader reproductive spectrum, rather than being confined to specific parity groups.

Regarding the socio-demographic variables extracted (age, education, marital status, and gender), these were selected based on their well-established relevance in fertility research and their linguistic inferability in the context of Chinese-language social media. Each variable could be reasonably inferred through self-disclosure or stable lexical patterns, and has been previously identified as a key determinant in fertility intention studies. These justifications have been added to the Variable Extraction section.

We also appreciate the reviewer’s comment on the need for a more robust justification of gender inclusion. We have revised the relevant paragraph to clarify that gender was derived not simply because women are central to fertility decisions, but because gendered reproductive roles result in asymmetrical material, biological, and institutional impacts—particularly in contexts such as childbearing costs, career disruption, and caregiving expectations. These differences are especially pronounced in the Chinese context, and thus justify our analytic focus on female-identified users. This updated rationale is now reflected in both the Introduction and Methods sections.

（3）Variables are still not clearly defined in the text (e.g. psychological adaptation, workplace equity, DINK lifestyle which is only defined in the discussion). I’d suggest adding an appendix that clearly outlines what kind of sentiment would fall under each of these groupings and a rough definition so the reader can understand what each category means.

**Response:**

We appreciate the reviewer’s attention to definitional precision and analytic clarity. In the revised manuscript, we have included a comprehensive glossary (Appendix B) outlining key discourse constructs such as psychological adaptation, workplace equity, and DINK lifestyle. Each entry provides an operational definition along with representative sentiment expressions drawn from the user-generated content (UGC) corpus.

These categories were not defined a priori but emerged inductively through semantic clustering of high-weighted TF-IDF terms and sentiment co-occurrence patterns. Final labels were validated through expert review to ensure interpretive consistency. This addition improves transparency and helps clarify the analytic scope of each thematic construct within the fertility discourse.

（4）The use of ‘fertility intentions’ is still incorrect and misleading. In the psycho-social literature this specifically means an active intention to execute a plan, so either actively planning to achieve or avoid a pregnancy. This is not the outcome variables the authors use, which is much closer to a general fertility attitude or desire, and I would recommend calling it such with citations from the literature to support the language choice.

**Response:**

We appreciate the reviewer’s concern regarding the precise use of terminology. In response, the manuscript now clarifies that the term fertility intentions is used in line with Spéder’s (2014) broader conceptualization—as encompassing both “plans or desires” to have children. This usage reflects an attitudinal orientation shaped by structural, psychological, and sociocultural factors, rather than a narrowly behavioral commitment.

While we acknowledge that the psychosocial literature often reserves “intention” for concrete behavioral plans, our analytic framework—grounded in the cognitive–social model, pathway framework, and Theory of Planned Behavior (TPB)—explicitly situates fertility intentions along a continuum between attitude and action. This integrated model supports our focus on dispositionally expressed fertility orientations, as observed in naturalistic discourse, and avoids conflating them with measurable behavioral outcomes.

This clarification has been incorporated into Section 2.3.1: Theoretical Model of Fertility Intentions, along with supporting citations to delineate our definitional boundaries.

（5）Thanks to the authors for adding the descriptive table of the sample, but they need to be clearer in text that anyone with any missing socio-demographic information was excluded from the sample. They say this in their response to reviewers but not in text, and it introduces limitations. For instance, it’s not clear how much of the sample had to be excluded because of this.

**Response:**

We appreciate the reviewer’s attention to data transparency. In the revised manuscript, we have explicitly stated in the Data Collection and Variable Extraction sections that all entries missing core socio-demographic variables—specifically age, education, or marital status—were excluded from the final analytical sample. This exclusion procedure affected 9,600 users, accounting for 41.5% of the initially processed dataset, and is now clearly reported in the descriptive summary and visualized in Figure 1 (line 104).

The implications of this exclusion are addressed at the end of Section 2.1.2, where we specify the number of omitted cases, explain the rationale for excluding records with unresolved demographic attributes, and note the inferential uncertainty associated with indirect variable extraction. These clarifications ensure that the scope and interpretive boundaries of the final dataset are transparent to the reader.

e limitations section is still very minimal. It currently only addresses the point I raised in
三、Presentation of findings
（1）The discussion is better than in the first version, and I am glad the authors have made an effort to provide citations and a more interesting narrative accompanying their findings. However, several issues remain including:
1. The authors seem to cherry-pick which results they discuss in the discussion. This is particularly striking given their largest effect was age and this isn’t discussed explicitly at all. I would suggest framing each part of the discussion first with a finding of interest and then elaborating on explanations/whether this aligns with previous findings. This will also eliminate some of the paragraphs that don’t seem to link to any findings at all e.g. lines 398-410.

**Response:**

We appreciate the reviewer’s comments on the improvement of the discussion and their helpful suggestions for further refinement. In response, we have restructured the discussion section to align more closely with the empirical results. Each thematic subsection now begins with a specific model finding—framed through odds ratios or TF-IDF salience scores—followed by interpretation, comparison to existing literature, and theoretical integration.

We have also addressed the reviewer’s concern regarding the omission of age. A new paragraph has been added explicitly discussing the magnitude and asymmetry of the age-related effects identified in the regression model.

Additionally, paragraphs that previously lacked clear empirical grounding have been either removed or integrated into adjacent, data-driven sections to ensure all arguments are directly supported by observed findings. These revisions aim to improve interpretive coherence and maintain alignment between results and discussion.

2. The authors present new findings in the discussion which does not follow writing conventions. If a finding appears in the discussion, it must have previously been mentioned in the results. For example, ‘In our sample, 36.8% of women indicated that their ideal age for childbearing is after 35 years old, or when they have achieved full financial independence, placing them in a difficult position”. This analysis (or the method through which it is derived) is not presented anywhere.

**Response:**

We appreciate the reviewer’s reminder regarding academic writing conventions. In the revised manuscript, we have carefully reviewed the discussion to ensure that no new findings are introduced at that stage. Specifically, the sentence referencing “36.8% of women indicating their ideal age for childbearing is after 35 years old or upon achieving financial independence” has been removed, as this specific statistic was not previously presented in the results.

We have cross-checked the entire discussion section to confirm that all referenced findings are traceable to the results section or associated tables and figures. These revisions ensure that the discussion remains strictly interpretive, rather than descriptive or data-generating.

（2）The discussion also has many unsupported conclusions. For example:
1. They say that ‘socioeconomic pressures [constitute] the paramount deterrent’ but provide no evidence from their analysis to support that conclusion.
2. Similarly they say ‘[the findings highlight] the synergistic effects of intersecting dimensions: economic barriers amplify career-related delays, which exacerbate psychological strain, all unfolding against a backdrop of shifting cultural paradigms.’ This is a completely unsupported conclusion – not only do the authors not provide any evidence from their analysis but I actually don’t think they could. Such a statement could only be supported through some kind of mediation analysis, not the current multivariate regression with no stepwise inclusion of variables.
3. “The psychological landscape reveals what we term “ambivalent agency”—a paradoxical empowerment through refusal.” Why have the authors decided to create this term that serves no greater purpose in the conclusions of the paper? It also doesn’t make sense. Why is empowerment through refusal paradoxical? The language in this part is also incredibly loaded, describing women as “weaponizing” reproductive agency. This point needs to be clarified and made much more neutral. A few sentences later they also say ‘educational attainment becomes both armor against traditional gender roles and prison limiting reproductive choices.’ What do the authors mean by prison limiting here? This seems dramatic unless there are actual criminal consequences for childbearing choices in China I am not familiar with.
4. “The psychological toll of these pressures cannot be overstated. Fertility anxiety, fueled by financial instability, career disruption fears, and societal expectations of "intensive mothering"39,40,41, permeates reproductive decision-making.” Again, what evidence from the analysis do the authors have to make this conclusion?

**Response:**

We appreciate the reviewer’s detailed critique regarding interpretive clarity and empirical boundaries. In the revised manuscript, we have carefully reviewed the discussion and made the following substantive revisions to ensure all statements remain analytically grounded:

1. The prior reference to “socioeconomic pressures as the paramount deterrent” has been removed. While economic variables (e.g., housing costs, childrearing expenses) exhibit strong associations with negative fertility attitudes, our analysis does not support the exclusive prioritization of these over other significant predictors such as psychological stress or sociocultural framing. The revised phrasing adopts a more balanced framing of multidimensional deterrents.
2. The phrase describing “synergistic effects of intersecting dimensions” has also been removed. As noted by the reviewer, our model does not test interaction or mediation effects; therefore, claims of directional amplification or causal interdependence exceed the scope of our empirical design. The revised version now treats these factors as independently associated influences.
3. Emotive or metaphorical expressions—such as “ambivalent agency,” “weaponizing agency,” and “education as both armor and prison”—have been either removed or rewritten in neutral academic language. These phrases introduced unnecessary conceptual ambiguity and lacked operational clarity within the scope of our analysis.
4. The sentence “the psychological toll cannot be overstated” has been revised to better reflect the statistical results. While psychological stressors were significantly associated with negative sentiment in both discourse and regression models, we agree that generalizations about fertility anxiety or motherhood norms should remain cautious and limited to model-supported domains.

These changes ensure that the discussion is consistent with our analytical evidence, avoids rhetorical overreach, and maintains precise, replicable language throughout.

四、Some new issues have also arisen as a result of the rewrite:
（1）I asked the authors to clarify their methods which they have done in their response letter. As a result, I can understand more about the analysis and its limitations. The classification of socio-demographic characteristics based on assumptions about names, pictures and stated facts has many limitations which the authors do not discuss. It also raises ethical questions about this approach and it makes the lack of ethical approval for this research concerning. The authors say in their ethics statement that ethics approval wasn’t needed because no personally identifiable information was collected. However, in the response to reviewers they say that usernames, gender, pronouns and IP addresses were all collected, which makes this a dataset with personal identifiers. This concern remains whether this data is publicly available or not.
（2）The authors have redone their figures, but the new versions are not appropriate. Sankey plots should be used to show movement from one group to another over time (figure 1), the radial plot adds no additional dimension beyond a normal bar chart (figure 2), and the two-way bar chart (figure 3) inexplicably groups neural/positive on one side and negative on the other. I would suggest converting all of these into conventional bar charts for easy interpretation by the reader. Figure 4 needs a label for the colour key as well.
（3）Table 1 has a footnote saying that some variables have weighting adjustments but I can’t see any variables with the symbol in subscript (sorry if an error with my software).
（4）How does one interpret the TF-IDF outputs in table 2? What do the numbers in the table mean? Without an explanation I cannot evaluate whether what the authors say in text is correct – they say things like ‘negative intentions were predominantly driven by housing cost (TF-IDF =2.34)’, but as I have no idea how this number was generated I can’t tell whether this is the correct conclusion. My lay understanding is it’s some kind of measure of personal importance to the individual? If that understanding is correct, is the above sentence right? Or is it that people had negative intentions rated the issue of the housing cost more strongly than those in other intentions groups who raised the same issue?
（5）Why is it that only some external factors are used as predictive variables in the regression and not others? The authors don’t provide a justification for this and it therefore appears cherry-picked to show the most convincing results. Have they grouped some of the reasons in the TF-IDF analysis together to form a thematic group? If so they need to explain how they did this and the robustness of their grouping choices.
（6）Table 4 is very difficult to interpret – if the independent variable is categorical, the authors need to list each variable in the first column and then each category in the second column (outlining which category is the reference). Without this I could not be sure how the authors have modelled some variables – for example it looks like age and year are modelled continuously whereas the authors talk about age as a categorical variable in text, and year could also arguably be modelled as categoric rather than discrete. Again, this raises concerns about the validity of the conclusions.
（7）It’s not hugely surprising the authors find limited statistical evidence of a link between external factors and positive intentions relative to neutral ones given so few of the sample have this outcome, making the analysis underpowered. The authors can say as such in text.

**Response:**

We thank the reviewer for the detailed and constructive observations regarding methodological clarity, figure design, variable specification, and the interpretation of statistical results. We have addressed each point systematically:

1. On socio-demographic inference and ethical approval: We appreciate the reviewer’s careful attention to ethical considerations. In our study, all socio-demographic variables were derived either from voluntarily disclosed statements in user-generated content (e.g., references to occupation, marital status, or age) or inferred through widely used linguistic cues (e.g., generational terms, geographic mentions). No usernames, profile images, or directly identifying metadata were collected, stored, or used for analysis. While IP information was used temporarily to validate province-level consistency, it was not linked to individual identifiers or retained beyond the initial filtering process. We have revised the Ethics Statement to clarify that all procedures complied with the Personal Information Protection Law (PIPL) of the People’s Republic of China, and that no personal data were handled in a manner that would require formal ethics approval under current national and institutional guidelines.
2. On figure design and visual clarity: We appreciate the reviewer’s professional suggestion. As advised, we have replaced the grouped bar chart with a standard format and adjusted all figures to include clear labels and units to enhance academic readability.
3. On Table 1 and weighting notation: We apologize for the confusion caused by the incorrect subscript annotation. In this study, only the data related to the three platform sources were adjusted using sample weights; all other variables are reported as raw values. The notation has been corrected in the revised table to avoid further misunderstanding.
4. On interpreting TF-IDF values in Table 2: TF-IDF values reflect the lexical prominence of a term within a sentiment category (positive/neutral/negative), adjusted for its frequency across the full corpus. Higher TF-IDF scores indicate that a term was disproportionately associated with a given sentiment relative to its background frequency. For example, a TF-IDF value of 2.34 for “housing cost” under negative sentiment means this term was highly concentrated in negative posts and minimally present in other sentiment contexts. This metric does not imply personal valuation by individual users, but rather corpus-level lexical salience. We have revised the main text and added clarification in a new footnote below Table 2.
5. On the selection of external factors for regression analysis: Thank you for raising this point. As clarified in the revised Methods section, all external factors were initially identified through TF-IDF scores, which reflect their weighted prominence in user discourse. Given the large number of extracted terms, we applied a clustering process to group semantically related items into broader thematic categories (e.g., “Socioeconomic Status,” “Socio-Cultural Factors”). These grouped themes—rather than individual terms—were then included in the regression model to ensure interpretability and avoid overfitting.
6. On regression model variable handling in Table 4: We appreciate the reviewer’s feedback on tabular clarity. The revised Table 4 now adopts a two-column format: the first lists each variable, and the second specifies each category and corresponding reference group (e.g., “Age: 20–30 [ref: <20]”). We confirm that age and year were modeled as categorical variables (not continuous), and the reference levels used in the regression have been explicitly defined in both the table and the text.
7. On low statistical power for the positive intention group: We fully agree with this assessment. The final dataset includes a small proportion of users classified under positive fertility attitudes. In the revised Discussion, we now acknowledge that the lack of strong associations between external variables and positive intentions may reflect limited statistical power, rather than an absence of meaningful relationships.

Together, these revisions strengthen the transparency and rigor of our methods, improve the readability of visualizations, and ensure appropriate interpretation of our findings within the empirical constraints of the study.

**Response to Reviewer 4:**

**The questions:**

This topic is interesting and relevant. However, I have some concerns

Introduction: Thank you for providing a detailed introduction which provides a comprehensive background and context for your scoping review. However, the author(s) should strengthen this section by:
1. Including the percentage of women who were TikTok users in Kuaishou, Xiaohongshu, Bilibili, and Weibo

Methodology: While the author(s) has provided some details on these sections, yet I believe these sections could have been improved as follow
Data Processing: It would be valuable to include a discussion of any socioeconomic factors considered during this stage, as they could provide additional context and strengthen the analysis.

**Response:**

1．Introduction — on platform demographics: We thank the reviewer for highlighting the importance of platform-specific user demographics. In the revised Introduction, we have incorporated the female user proportions of Douyin (69.2%), Kuaishou (44.8%), and Xiaohongshu (68%), along with their respective monthly active user (MAU) bases. These figures help contextualize the gendered landscape of user-generated fertility discourse and further justify our platform selection strategy. As noted in the manuscript, Weibo and Bilibili were excluded based on demographic and content alignment grounds, specifically their lower female user representation and limited engagement with sustained fertility narratives.
2．Methodology — on socioeconomic context in data processing: We thank the reviewer for this valuable suggestion. Due to the inherent limitations of user-generated content, obtaining precise socioeconomic variables—such as income or household status—was not feasible, similar to the challenge of reliably capturing parity information. As a result, we relied on indirect demographic proxies (e.g., education level, occupation, city tier) where available, which serve as reasonable correlates of socioeconomic status in this context. In future work, we aim to integrate platform-based user tagging systems, survey-linked accounts, or AI-enhanced profile classification to improve the resolution of socioeconomic inference and partially address this limitation.
